# Novel 4-thiophenyl-pyrazole, pyridine, and pyrimidine derivatives as potential antitumor candidates targeting both EGFR and VEGFR-2; design, synthesis, biological evaluations, and *in silico* studies†

**DOI:** 10.1039/d3ra00416c

**Published:** 2023-04-18

**Authors:** Samia M. Al-Muntaser, Ahmed A. Al-Karmalawy, Abeer M. El-Naggar, Ali Khalil Ali, Nour E. A. Abd El-Sattar, Eslam M. Abbass

**Affiliations:** a Department of Chemistry, Faculty of Science, Ain Shams University Abbassiya 11566 Cairo Egypt eslammorad@sci.asu.edu.eg; b Pharmaceutical Chemistry Department, Faculty of Pharmacy, Ahram Canadian University 6th of October City Giza 12566 Egypt akarmalawy@acu.edu.eg

## Abstract

In this article, we continued our previous effort to develop new selective anticancer candidates based on the basic pharmacophoric requirements of both EGFR and VEGFR-2 inhibitors. Therefore, twenty-two novel 4-thiophenyl-pyrazole, pyridine, and pyrimidine derivatives were designed and examined as dual EGFR/VEGFR-2 inhibitors. Besides, the previously reported antimicrobial activities of the aforementioned nuclei motivated us to screen their antibacterial and antifungal activities as well. First, the antitumor activities of the newly synthesized derivatives were evaluated against two cancer cell lines (HepG-2 and MCF-7). Notably, compounds 2a, 6a, 7a, 10b, 15a, and 18a exhibited superior anticancer activities against both HepG-2 and MCF-7 cancer cell lines. These candidates were selected to further evaluate their anti-EGFR and anti-VEGFR-2 potentialities which were found to be very promising compared to erlotinib and sorafenib, respectively. Both 10b and 2a derivatives achieved better dual EGFR/VEGFR-2 inhibition with IC_50_ values of 0.161 and 0.141 μM and 0.209 and 0.195 μM, respectively. Moreover, the most active 10b was selected to evaluate the exact phase of cell cycle arrest and to investigate the exact mechanism of cancer cell death whether it be due to apoptosis or necrosis. On the other hand, all the synthesized compounds were tested against Gram-positive bacteria such as *S. aureus* and *B. subtilis* as well as Gram-negative bacteria such as *E. coli* and *P. aeuroginosa*. Also, the antifungal activity was investigated against *C. albicans* and *A. flavus* strains. The findings of the antimicrobial tests revealed that most of the investigated compounds exhibited strong to moderate antibacterial and antifungal effects. Furthermore, to understand the pattern by which the investigated compounds bound to the active site, all the newly synthesized candidates were subjected to two different docking processes into the EGFR and VEGFR-2 binding sites. Besides, we tried to correlate compound 10b and the reference drugs (erlotinib and sorafenib) through DFT calculations. Finally, following the biological data of the new pyrazole, pyridine, and pyrimidine derivatives as anticancer and antimicrobial candidates, we concluded a very interesting SAR for further optimization.

## Abstract 2

Novel 4-thiophenyl-pyrazole, pyridine, and pyrimidine derivatives as potential dual EGFR/VEGFR-2 inhibitors with promising antimicrobial activity as well.

## Graphical abstract

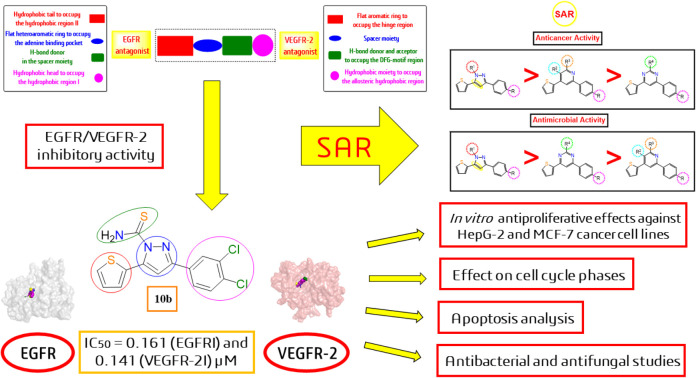

## 1. Introduction

One of the most dangerous diseases in the world that threatens human life is cancer. It is considered the second driving cause of death in the world.^1,2^ According to global statistics, cancer is considered one of the primary causes of death worldwide.^3,4^

Nowadays, medicinal chemists are requested to find out effective and specific chemotherapeutic agents.^5,6^ Such drugs are designed for inhibiting cancer cell growth by way of interacting with particular molecular targets resulting in substantial harm to the cancerous cells.^7,8^ However, some problems like resistance, delivery, and/or selectivity may diminish the process of drug development.^9,10^ Therefore, there is a great interest to understand the cellular and molecular pathways that contribute to the process of cancer initiation and/or spread.^11,12^

Protein kinases (PKs) constitute crucial protein families in the process of diverse disease propagation such as cancer, diabetes, and/or inflammation. Therefore, they constitute a very promising target for new drug discovery due to their prominent roles in several cellular functions like apoptosis, cell cycle, DNA damage/repair, and metabolism.^13–16^

**Fig. 1:**
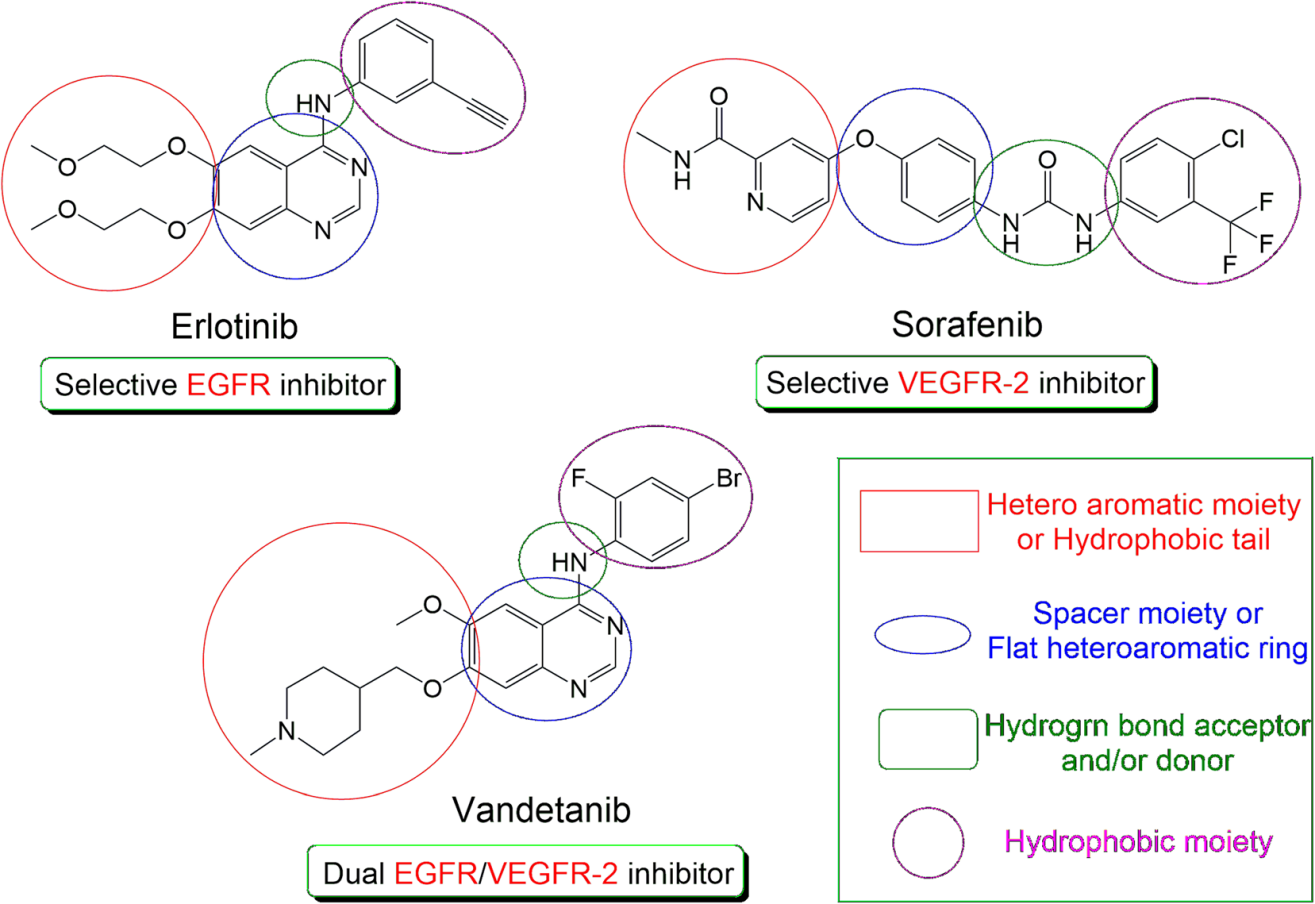
The common pharmacophoric features of the FDA-approved drugs (erlotinib, sorafenib, and vandetanib).

Many types of tumors such as colon, breast, and ovarian subtypes resulted from the overexpression of EGFR (epidermal growth factor receptor). EGFR is a transmembrane PK receptor that is responsible for cell proliferation and/or apoptosis through different signal transduction pathways. It was recorded to be involved in the process of angiogenesis which increases the proliferation of tumor cells, invasiveness, and metastasis.^17^ EGFR inhibitors (*e.g.* erlotinib)^18^ are one of the most important FDA-approved drugs for cancer treatment, Fig. 1.

Besides, EGFR stimulation results in increasing the expression of VEGF (vascular endothelial growth factor) which is the main factor that is responsible for tumor angiogenesis after its binding to VEGFR-2 (vascular endothelial growth factor receptor-2).^19^ Therefore, FDA-approved VEGFR-2 inhibitors (*e.g.* sorafenib)^20^ are considered potential anticancer drugs, Fig. 1.

Due to the close relationship between the two previously discussed pathways, we can confirm that EGFR blocking will result in decreasing the endothelial expression of VEGF. Also, VEGFR-2 inhibition will potentiate the anticancer activity of EGFR inhibitors.^21^ Based on the above, the dual inhibition of EGFR and VEGFR-2 represents a very promising protocol for cancer treatment.^22^ One of the most potent dual EGFR/VEGFR-2 inhibitors is vandetanib (Fig. 1) which is an FDA-approved drug against thyroid cancer.^23^

According to Fig. 1, we can observe the common and greatly similar pharmacophoric features of EGFR and/or VEGFR-2 inhibitors. Both types require the presence of four pharmacophores which are the hydrophobic head, H-bond donor (for EGFRI) or H-bond donor and acceptor (for VEGFR-2I), spacer flat heteroaromatic ring, and hydrophobic tail.^14,24,25^

Pyrazole, pyridine, and pyrimidine systems constitute very promising scaffolds for many anticancer agents. They were previously reported as potential EGFRIs,^26,27^ VEGFR-2Is,^28,29^ and/or dual EGFR/VEGFR-2 inhibitors.^30,31^ Based on the aforementioned facts, we aimed to develop novel 4-thiophenyl-pyrazole, pyridine, and pyrimidine derivatives as promising inhibitors against both EGFR and VEGFR-2. On the other hand, the previously mentioned moieties showed apparent antimicrobial activities^32,33^ which encourages us to screen the antibacterial and antifungal activities of the newly designed targets.

### 1.1. Rationale and work design

Based on the above, we designed and introduced novel 4-thiophenyl-pyrazole, pyridine, and pyrimidine derivatives as potential antitumor candidates targeting both EGFR and VEGFR-2 receptors.

**Fig. 2:**
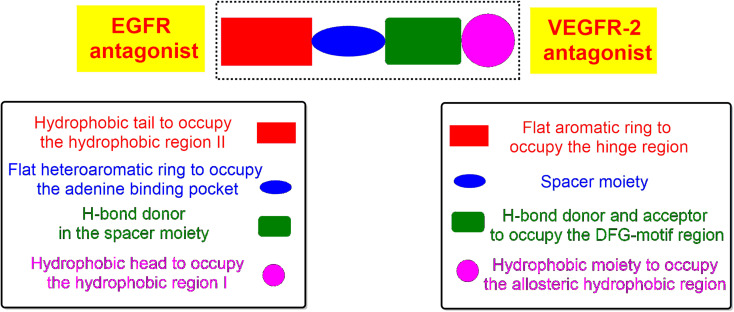
Pharmacophoric features of both EGFR and VEGFR-2 antagonists.

Briefly, the principal pharmacophoric features of EGFR inhibitors (Fig. 2) were identified as follows:^2^

(a) Hydrophobic head to bind the hydrophobic region I.

(b) H-bond donor in the spacer region.

(c) Flat heteroaromatic ring to bind the adenine binding pocket.

(d) Hydrophobic tail to bind the hydrophobic region I.

However, the pharmacophoric features of VEGFR-2 inhibitors (Fig. 2) were identified as:^34^

(a) Flat aromatic ring to occupy the hinge region.

(b) Spacer moiety.

(c) Both the H-bond donor and acceptor to bind the DFG motif.

(d) Hydrophobic moiety to bind the allosteric hydrophobic region.

Therefore, the new design is based on the introduction of the thiophene ring to act as a hydrophobic tail and bind the hydrophobic region II of EGFR or the hinge region of VEGFR-2. Besides, the pyridine or pyrazole moiety to bind the adenine binding pocket of EGFR or to act as a spacer for the VEGFR-2 receptor. Moreover, different functional groups were introduced as H-bond donors and/or acceptors to form H-bonds in the spacer region of EGFR or the DFG-motif region of VEGFR-2. Finally, a *p*-hydroxyphenyl ring was added to occupy the hydrophobic region I of EGFR or the allosteric hydrophobic region of VEGFR-2.

Accordingly, the main aim of this new design is to target both EGFR and VEGFR-2 receptors even if not all the pharmacophoric features are present typically in the new candidates. The atypical design of some new derivatives may give them adequate flexibility to bind both receptors which may be advantageous.

On the other hand, the previously reported antibacterial and/or antifungal activities of the pyrazole, pyridine, and pyrimidine derivatives motivated us to further screen the antibacterial and antifungal activities of the newly synthesized candidates.

## 2. Results and discussion

### 2.1. Chemistry

The α, β unsaturated ketone derivatives are highly reactive molecules that contain two reactive centers to be used as a bidentate reagent in the synthesis of different heterocyclic moieties.^34,35^ On the other hand, active methylene and cyclic active methylene compounds like dimedone, 1,3-indandione, and its derivatives were approved for their anticancer activities and were also used as key starting materials for the synthesis of biologically active heterocyclic compounds.^36,37^

**Scheme 1:**
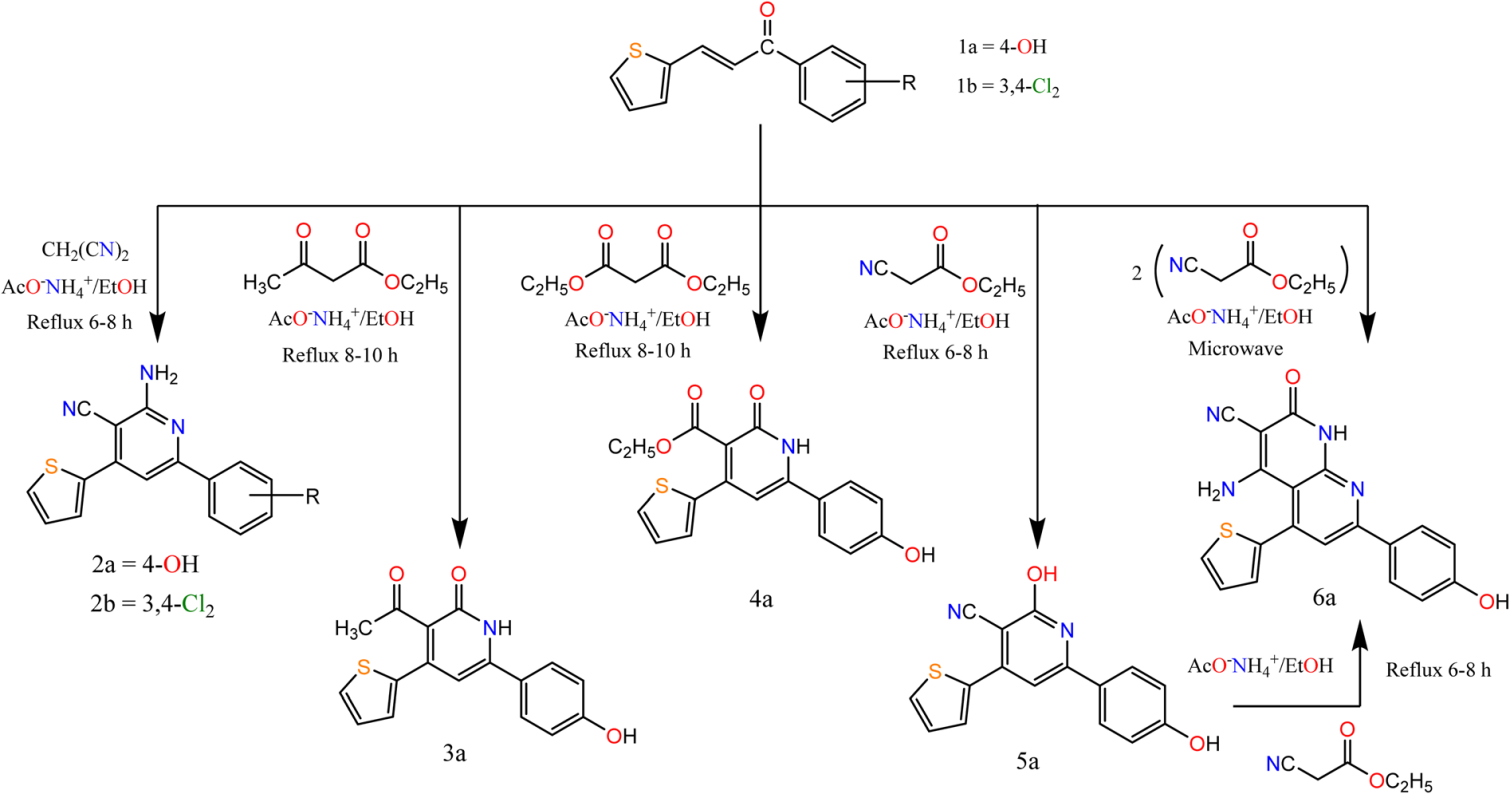
Synthesis of the target compounds (2a, b), 3a, 4a, 5a, and 6a.

Thus, we synthesized a polarized system as 1-(aryl)-3-(thiophen-2-yl)prop-2-en-1-one “chalcone”, and then accomplished by ring closure to afford novel heterocyclic compounds (pyridine and pyrazole). Claisen–Schmidt condensation^38,39^ of substituted ketone derivatives with thiophene-2-carboxaldehyde afforded chalcones 1a and 1b (Scheme 1).

According to the previously reported methods, the derivatives were prepared at a high yield. The chalcone derivatives and suitable nucleophile compounds were heated under reflux conditions and utilized for the synthesis of various pyrimidine and pyrazole derivatives. All the target compounds 1a–20a were characterized using spectral data such as ^1^H NMR, mass, and IR spectroscopy. Moreover, the purity of the previously mentioned derivatives was confirmed by elemental analyses as depicted in the ESI.†

The spectral data of chalcone 1b laid a strong foundation for a confirmation of the chemical structure and stereochemistry of 1b. The IR spectrum exhibited an absorption band at 1655 cm^−1^ characteristic of functionalities of C=O. Moreover, the ^1^H NMR spectrum of chalcone 1b indicated the presence of two doublets at 7.57 and 7.92 ppm corresponding to β and α protons of C=C, respectively, with *J* = 14.8 Hz.

In this paper, we study the behavior of chalcones 1a, 1b towards some C- and N-nucleophilic reagents. So, we synthesized new heterocyclic compounds by refluxing chalcone 1a and or 1b in ethanol in the presence of ammonium acetate with malononitrile, ethyl acetoacetate, and diethyl malonate afford the pyridine derivatives 2a–6a respectively. In the case of ethyl cyanoacetate addition of one molecule afforded compound 5a while the addition of two molecules afforded compound 6a. Their structures were confirmed by recording their spectral data. For compounds 2a and 2b. The IR spectrum showed bands at the range 3223–3362 and 2200–2213 cm^−1^ attributed to NH_2_ and CN groups respectively. The ^1^H NMR spectra of compounds 2a and 2b exhibited a singlet signal at *δ* 7.15 and 6.91 attributed to NH_2_ protons, respectively.

However, the IR spectrum of compound 3a showed bands at 3107 and 1695 cm^−1^ attributed to NH and CO groups respectively. The ^1^H NMR spectrum exhibited a singlet at *δ* 2.50 and 9.86 ppm, attributed to CH_3_–CO and OH protons, respectively. For compound 4a its IR spectra exhibited bands at 3170, 1725, and 1675 cm^−1^ attributed to NH and 2 CO groups. The ^1^H NMR spectrum exhibited signals at *δ* 1.11, 3.91, and 7.19 ppm, attributed to CH_3_, CH_2_, and NH proton, respectively. For compound 5a its IR spectra exhibited bands at 3301, 2216, and 1636 cm^−1^ attributed to OH, CN, and C=N groups, respectively. The ^1^H NMR spectrum exhibited a singlet at *δ* 10.22, and 12.48 ppm, attributed to two OH protons. The spectral data of compound 6a confirmed the expected structure. IR spectra exhibited bands at 3369, 2217, and 1683 cm^−1^ attributed to NH, CN, and CO groups. The ^1^H NMR spectrum exhibited a singlet at *δ* 6.47 and 9.81 ppm, attributed to NH_2_, and NH proton, respectively.

**Scheme 2:**
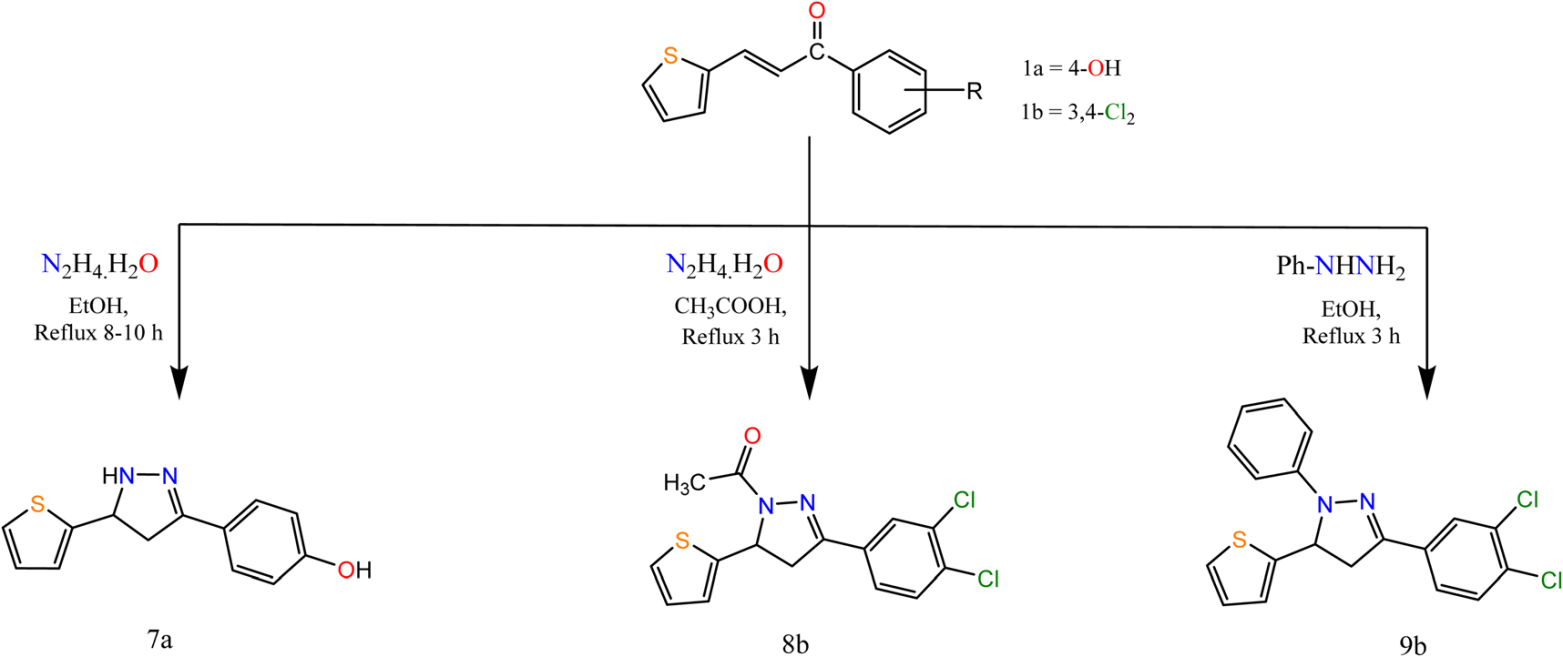
Synthesis of the target compounds 7a, 8b, and 9b.

Moreover, the reaction of α,β-unsaturated ketones with hydrazine hydrate and its derivatives (Scheme 2) for synthesis, pyrazolines is one of the most convenient methods. Pyrazoline derivatives 7a–9b were synthesized by the reaction of chalcones 1a and/or 1b with hydrazine hydrate in ethanol, in glacial acetic acid and phenylhydrazine, respectively *via* aza Michael addition of hydrazine on chalcone 1b, followed by a 5-*exo*-trig ring cyclization and dehydration. Elemental analyses, IR, and ^1^H NMR spectra supported the structure confirmation of compounds 7a–9b (Scheme 2).

The IR spectra of compounds 7a–9b showed bands at the range 1606–1636 cm^−1^ that account for the formation of the C=N bond. In addition, compound 8b showed a band at 1667 cm^−1^ attributed to CO. The appearance of CH_2_ proton at 2.82, 3.27, 3.24 ppm in the ^1^H NMR spectra of compounds 7a–9b respectively, confirmed the formation of the pyrazole ring, in addition to the appearance of signals at *δ* 5.05, 5.84 and 5.86 for compounds 7a–9b, respectively attributed to CH proton of pyrazole ring*.* Also, compound 8b showed a singlet signal at 2.48 ppm accounts for the CH_3_ protons.

**Scheme 3:**
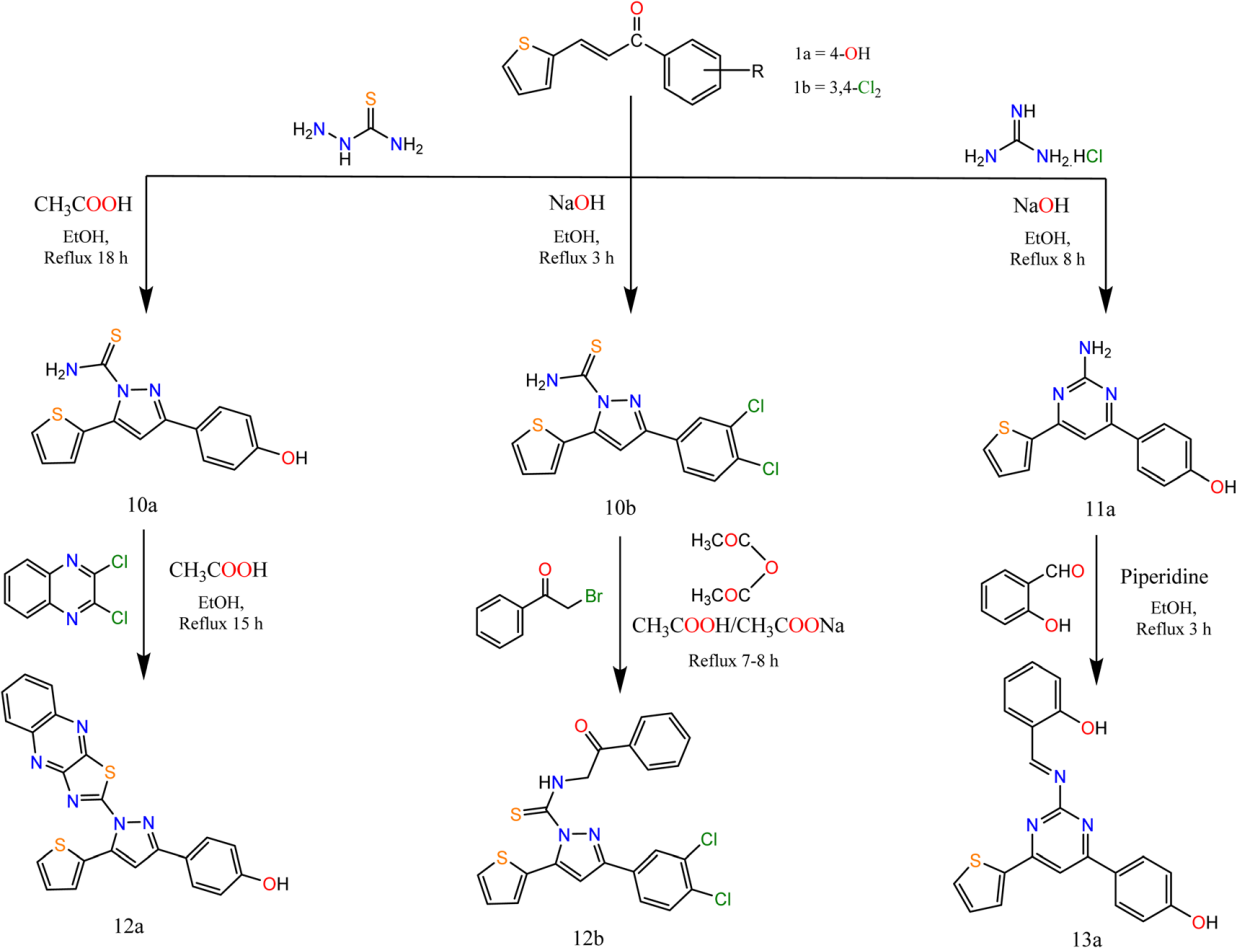
Synthesis of the target compounds 10a, 10b, 11a, 12a, 12b, and 13a.

Similarly, the reaction of chalcones 1a, b with thiosemicarbazide in two different conditions afforded pyrazole carbothioamide derivative, the first condition is refluxing of chalcone 1a in ethanolic solution in the presence of a catalytic amount of acetic acid afforded compound 10a the second condition is refluxing of chalcone 1b in ethanolic sodium hydroxide solution afforded compound 10b (Scheme 3).

Alkylation of both compounds 10a and 10b by 2,3-dichloroquinoxalin and phenacyl bromide afforded pyrazole derivatives 12a and 12b, respectively. Refluxing of chalcone 1a with guanidine hydrochloride in ethanolic sodium hydroxide solution afforded compound 11a which condensed with salicylaldehyde in ethanol with a catalytic amount of piperidine afforded pyrimidine derivative 13a (Scheme 3).

The IR spectra of compounds 10a, 10b, and 11a showed bands at the range 3145–3343 cm^−1^ that account for the presence of NH_2_ and showed bands at the range 1636–1639 cm^−1^ that account for the formation of C=N. In addition, compound 12b showed bands at 1668 and 2849 cm^−1^ attributed to CO and CH-aliphatic, respectively. The ^1^H NMR spectra of compounds 10a and 11a showed the appearance of singlet signals at 6.88, 6.99, and 7.65 ppm, attributed to CH protons of pyrazole and pyrimidine rings, respectively. In addition, compound 12b showed a singlet signal at 4.82 attributed to CH_2_ protons. Compound 13a showed singlet signals at 7.51 and 7.56 attributed to CH of pyrimidine and CH=N.

**Scheme 4:**
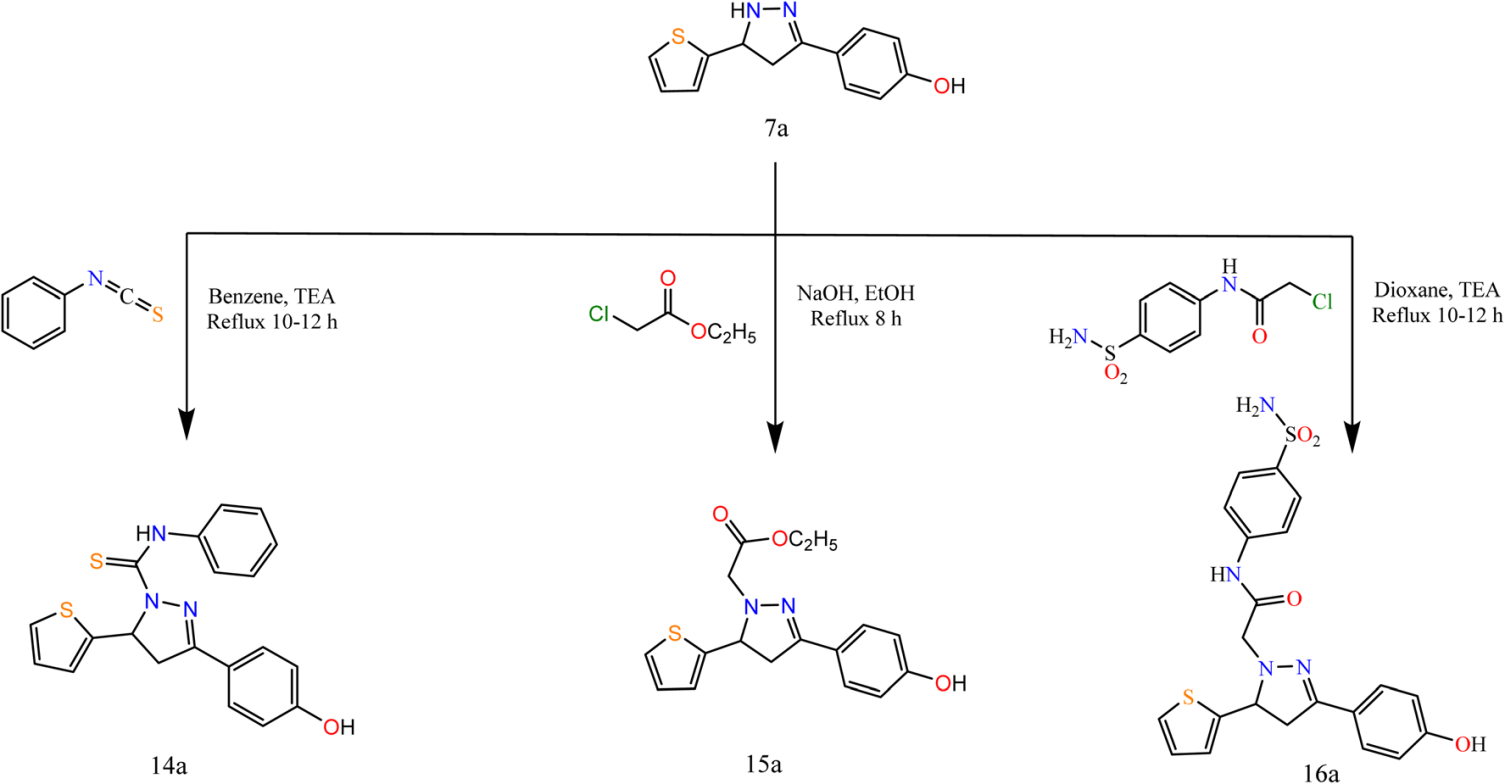
Synthesis of the target compounds 14a, 15a, and 16a.

Furthermore, to study the reactivity of pyrazole ring towards the electrophilic reagents as 7a with phenyl isothiocyanate, ethyl chloroacetate, and 2-chloro-*N*-(4-sulfamoylphenyl)acetamide in different conditions afforded pyrazole derivatives 14a–16a (Scheme 4).

The spectral data were in agreement with the predicted structure of the compounds. For compound 15a, the IR spectrum showed bands at 1731 cm^−1^ attributed to the CO group. Its ^1^H NMR exhibited triplet and quartet signals at 1.02 and 4.09 ppm attributed to CH_3_ and CH_2_ protons. For compound 16a, the IR spectrum showed bands at 1694 cm^−1^ attributed to the CO group. Its ^1^H NMR exhibited signals at 5.15 ppm attributed to CH_2_ protons of the acetamide group.

On the other hand, in extension to our previous work on the PTC alkylation of 1,3-disubstituted-2-pyrazolin-5-ones^40,41^ we have studied here the reactivity of pyrazole derivative towards PTC alkylation and acylation in the presence or absence of CS_2_.

**Scheme 5:**
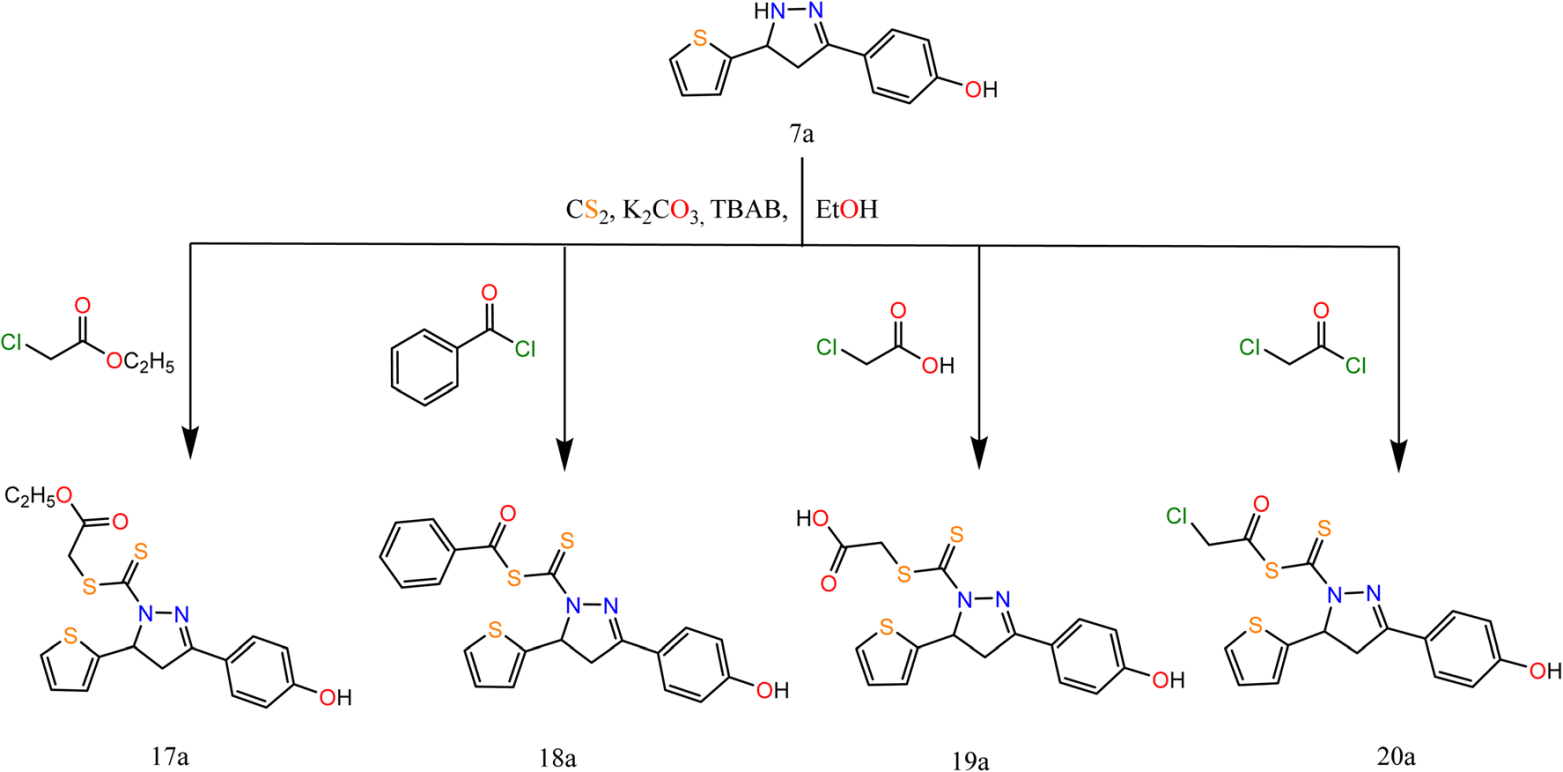
Synthesis of the target compounds 17a, 18a, 19a, and 20a.

Alkylation of pyrazole derivative (7a) by ethylchloroacetate, benzoyl chloride, chloroacetic acid, and chloroacetyl chloride in the presence of carbon disulfide under liquid/solid PTC reaction conditions afforded pyrazole-1-carbonothioyl 17a–20a (Scheme 5).

The IR spectra of 17a–20a showed bands at 1696, (1696, 1736), 1692, and (1746, 1694) cm^−1^ attributed to CO groups, respectively. For more conformation to the predicted structure. The ^1^H NMR of 17a exhibited signals at 4.06 ppm attributed to CH_2_ protons of the ester group. The CH_2_ group of acid present in compound 19a exhibited a singlet signal at 3.60 ppm. However, the CH_2_ of acetyl chloride in compound 20a exhibited a singlet signal at 4.35 ppm.

The reaction proceeds *via* the nucleophilic addition of pyrazole at N^−^ to CS_2_ followed by simultaneous *N*-alkylation of the intermediate thiocarbamic acid. The suggested mechanism is represented in the ESI.†

### 2.2. Biological evaluations

#### 2.2.1. Anti-proliferative activities

The antitumor activities of the newly synthesized derivatives against two cancer cell lines (HepG-2 and MCF-7), Table 1, based on their main nuclei (pyrazole-based, pyridine-based, and pyrimidine-based moieties) showed the following interesting results:

**Table 1:** Anti-proliferative activities of the newly designed 4-thiophenyl-pyrazole, pyridine, and pyrimidine derivatives against HepG-2 and MCF-7 cancer cell lines

Comp.	Cell lines (IC_50_ μM)	
	HepG-2	MCF-7
1a	87.56 ± 4.2	82.45 ± 4.2
1b	84.16 ± 4.2	>100
2a	8.42 ± 0.6	9.59 ± 0.8
2b	59.47 ± 3.1	55.46 ± 3.2
3a	53.21 ± 3.1	48.35 ± 2.7
4a	44.45 ± 2.6	41.22 ± 2.4
5a	43.03 ± 2.5	38.37 ± 2.5
6a	16.24 ± 1.3	19.12 ± 1.6
7a	8.13 ± 0.6	3.54 ± 0.2
8b	74.28 ± 3.5	72.64 ± 3.9
9b	48.11 ± 2.7	67.33 ± 3.7
10a	39.07 ± 2.4	23.72 ± 1.7
10b	13.81 ± 1.1	23.24 ± 1.8
11a	52.94 ± 2.9	61.08 ± 3.4
12a	74.73 ± 3.9	65.03 ± 3.6
12b	29.20 ± 2.1	35.95 ± 2.3
13a	34.76 ± 2.3	42.75 ± 2.6
14a	69.20 ± 3.6	54.81 ± 3.1
15a	21.38 ± 1.5	9.48 ± 0.7
16a	34.53 ± 2.2	29.06 ± 2.0
17a	62.41 ± 3.3	36.87 ± 2.2
18a	12.94 ± 1.0	6.92 ± 0.5
19a	28.20 ± 1.9	14.69 ± 1.2
20a	67.41 ± 3.5	56.19 ± 3.3
DOX	4.50 ± 0.2	4.17 ± 0.2

(a) Pyrazole-based moieties:

• Dihydropyrazole derivatives were more active than pyrazole ones against the two cancer cell lines. Dihydropyrazole candidates which do not contain *N*-substitution like 7a showed very strong activities against both HepG-2 and MCF-7 more than the *N*-substituted dihydropyrazoles.

• *N*-Substituted dihydropyrazole with CH_2_–CO-attracting group like 15a showed very strong activity against MCF-7 and moderate activity against HepG-2 while *N*-substituted dihydropyrazole with CH_2_–CO-donating group like 16a represented moderate activities against both cell lines.

• On the other hand, *N*-substituted dihydropyrazole with CS-S-G side chains as in compounds 17a–20a have higher activities on the MCF-7 than the HepG-2 cell line. They showed very strong to weak activities depending on the nature of the group (G) as follows:

(i) If G is an aliphatic acid derivative like 18a, it achieved very strong activity against MCF-7 and strong activity against HepG-2.

(ii) However, if G is a benzoyl derivative like 19a, it expressed strong activity towards MCF-7 and moderate activity towards HepG-2.

(iii) Besides, if G is an aliphatic ester derivative like 17a, it showed moderate activity against MCF-7 and weak activity with HepG-2.

(iv) While, if G is an acetyl derivative like 20a, it showed weak activity with both cell lines.

• Furthermore, the *N*-substituted pyrazole derivatives with CS-NH_2_ side chain like 10a and 10b showed strong to moderate activities depending on the substituted phenyl ring attached to pyrazole as follow:

(i) In the case of the activated ring like 4-hydroxy benzene in compound 10a, it showed moderate activities with both cell lines.

(ii) While, in the case of the deactivated ring like dichlorobenzene in compound 10b, it showed strong activity on HepG-2 and moderate activity on MCF-7.

(b) Pyridine-based moieties:

• Pyridine analogs containing the NH_2_ group in the alpha position to the attracting CN group like compounds 2a and 6a achieved very strong and strong activities against both cancer cell lines. The fused pyridopyridine derivative 6a showed lower activities than the pyridine derivative 2a.

• On the other hand, pyridines containing the OH group in the alpha position to the attracting groups (COCH_3_, COOEt, and CN) in 3a, 4a, and 5a compounds, respectively, showed moderate activities against the two cancer cell lines.

(c) Pyrimidine-based moieties:

• Pyrimidine derivatives (11a and 13a) showed moderate and weak anticancer activities depending on the substitution in position 2 of pyrimidine as follows:

(i) If position 2 contains a donating group like NH_2_ in compound 11a, it showed moderate antitumor activities on both cancer cell lines.

(ii) While, when NH_2_ functional group was condensed with the aromatic aldehyde like 13a, the activity decreased on both cell lines.

#### 2.2.2. EGFR and VEGFR-2 inhibitory activities

**Fig. 3:**
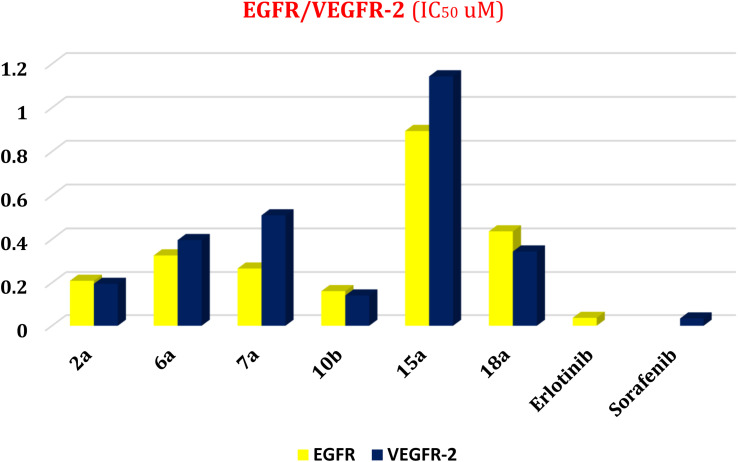
Effect of compounds (2a, 6a, 7a, 10b, 15a, and 18a) as dual EGFR/VEGFR-2 inhibitors.

The superior anticancer candidates (2a, 6a, 7a, 10b, 15a, and 18a) were selected to further evaluate their anti-EGFR and anti-VEGFR-2 potentialities (Fig. 3). All compounds showed very promising EGFR and VEGFR-2 μM inhibitory concentrations compared to erlotinib (0.037 μM) and sorafenib (0.034 μM), respectively. Notably, both 10b and 2a derivatives achieved the superior dual EGFR/VEGFR-2 inhibition with IC_50_ values of (0.161 and 0.141 μM) and (0.209 and 0.195 μM), respectively. Besides, compounds (6a, 7a, and 18a) scored strong dual EGFR/VEGFR-2 inhibition with IC_50_ values of (0.326 and 0.141 μM), (0.266 and 0.509 μM), and (0.436 and 0.344 μM), respectively. However, the least dual EGFR/VEGFR-2 inhibition was recorded for 15a candidate with IC_50_ values of 0.894 and 1.143 μM, respectively. Briefly, these μM inhibitory concentrations recommend greatly the proposed rationale design for the investigated compounds to act as promising dual EGFR/VEGFR-2 inhibitors.

#### 2.2.3. Cell cycle analysis

**Fig. 4:**
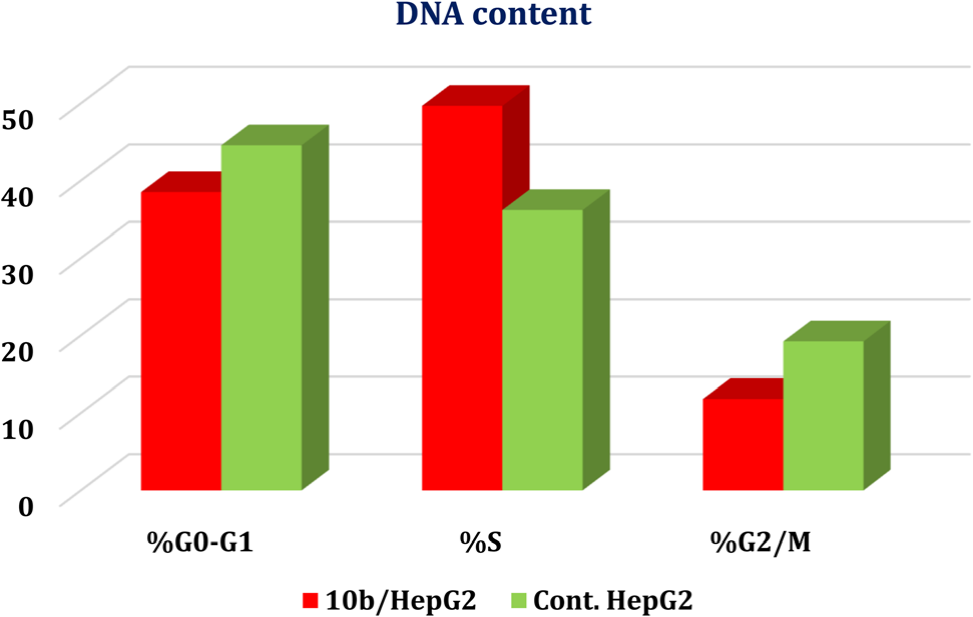
Cell cycle analysis of HepG-2 cells treated with 10b at a concentration of 13.81 μM.

The most active EGFR/VEGFR-2 inhibitor 10b was further selected to evaluate the exact phase of cell cycle arrest. Herein, the most sensitive cell line (HepG2) was treated with the IC_50_ value of 10b (13.81 μM) to record its impact on the different phases of cell growth (% G0–G1, % S, and % G2/M). Compound 10b showed a marked decline in cell populations at the S phase with 49.68% (1.37-fold) compared to that of the control (36.19%), Fig. 4.

#### 2.2.4. Apoptosis analysis

**Fig. 5:**
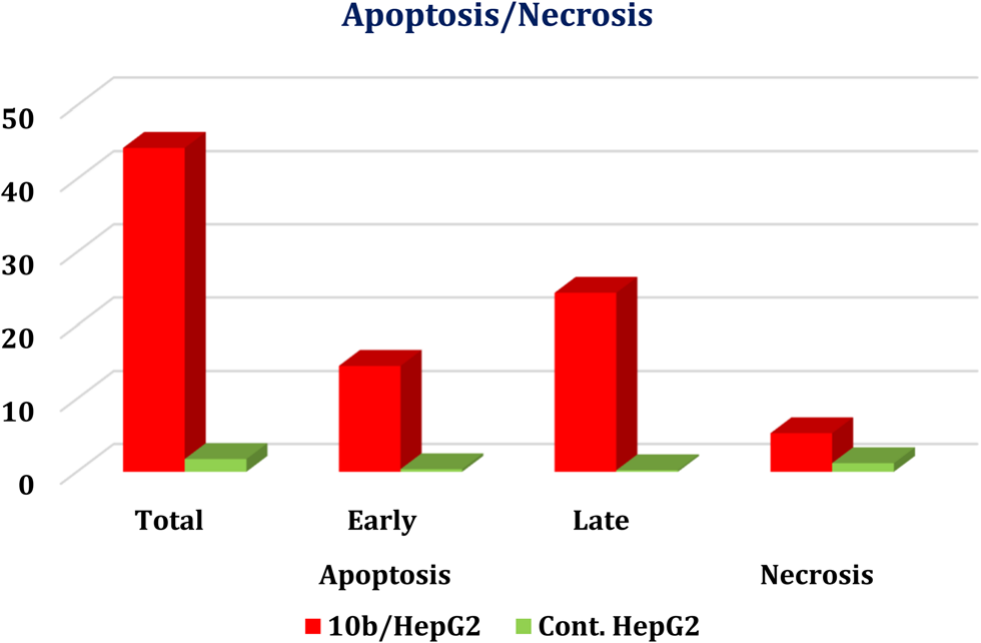
Induction of apoptosis in HepG-2 cells by 10b shows both apoptotic (total, early, and late) and necrotic cell death.

**Fig. 6:**
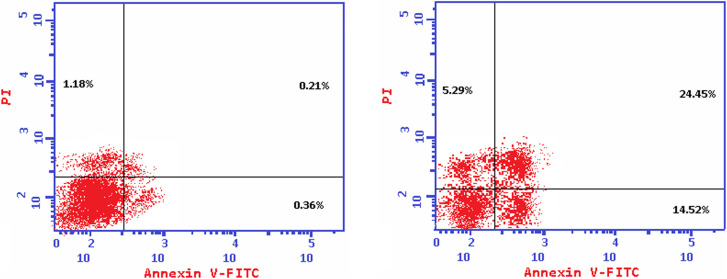
Histograms of annexin-V apoptosis assay for 10b in HepG-2 (lower right, upper right, lower left, and upper left represent early apoptotic, late apoptotic, viable, and necrotic phases, respectively).

Furthermore, the superior EGFR/VEGFR-2 inhibitor 10b was selected to investigate the exact mechanism of cancer cell death whether it be due to apoptosis (programmed cell death) or necrosis (uncontrolled cell death). Therefore, the treatment of the HepG-2 cancer cell line with the IC_50_ value of 10b (13.81 μM) revealed an apparent increase in the total apoptosis percentage (from 1.75 to 44.26), compared to the control. This was accompanied by significant elevations in the percentage of the AnxV-FITC apoptotic cells in both the early (from 0.36 to 14.52%) and late (from 0.21 to 24.45%) apoptotic phases, compared to the control (Fig. 5 and 6). Accordingly, we can confirm the exact mechanism of the anticancer activity of 10b to be due to the programmed apoptosis pathway.

#### 2.2.5. Antibacterial and antifungal activities

Using the inhibition zone approach and minimum inhibitory concentrations (MIC), all of the newly synthesized 4-thiophenyl-pyrazole, pyridine, and pyrimidine derivatives were tested for *in vitro* antibacterial and antifungal activities (Table 2). They were all tested against Gram-positive bacteria such as *Staphylococcus aureus* and *Bacillus subtilis* as well as Gram-negative bacteria such as *Escherichia coli* and *Pseudomonas aeuroginosa*. Also, the antifungal activity was investigated against *Candida albicans* and *Aspergillus flavus* strains. Gentamycin was used as the standard antibacterial drug whereas ketoconazole was regarded as the primary antifungal one.

**Table 2:** Antibacterial and antifungal activities of the newly designed 4-thiophenyl-pyrazole, pyridine, and pyrimidine derivatives

Comp.	Gram-positive bacteria				Gram-negative bacteria				Fungi			
	*S. aureus*		*B. subtilis*		*E. coli*		*P. aeuroginosa*		*C. albicans*		*A. flavus*	
	Diameter of inhibition zone (mm)	% Activity index	Diameter of inhibition zone (mm)	% Activity index	Diameter of inhibition zone (mm)	% Activity index	Diameter of inhibition zone (mm)	% Activity index	Diameter of inhibition zone (mm)	% Activity index	Diameter of inhibition zone (mm)	% Activity index
1a	NA	—	NA	—	NA	—	NA	—	3	10.7	NA	—
1b	NA	—	NA	—	NA	—	NA	—	NA	—	NA	—
2a	10	55.5	15	68.2	9	42.8	12	63.1	18	64.3	12	46.1
2b	9	50.0	14	63.6	8	38.1	11	57.9	17	60.7	10	38.5
3a	5	27.8	9	40.9	4	19.0	6	31.6	11	39.3	10	38.5
4a	6	33.3	10	45.4	5	23.8	8	42.1	13	46.4	11	42.3
5a	6	33.3	10	45.4	5	23.8	10	52.6	14	50.0	8	30.8
6a	5	27.8	9	40.9	4	19.0	7	36.8	12	42.8	8	30.8
7a	15	83.3	19	86.4	12	57.1	17	89.5	23	82.1	18	69.2
8b	3	16.7	7	31.8	NA	—	4	21.0	9	32.1	5	19.2
9b	NA	—	6	27.3	NA	—	NA	—	8	28.6	3	11.5
10a	9	50.0	13	59.1	8	38.1	12	63.1	13	46.4	11	42.3
10b	12	66.7	17	77.3	9	42.8	13	68.4	20	71.4	14	53.8
11a	7	38.9	10	45.4	6	90.5	11	57.9	11	39.3	7	26.9
12a	NA	—	5	22.7	NA	—	3	15.8	6	21.4	NA	—
12b	7	38.9	12	54.5	5	23.8	8	42.1	15	53.6	10	38.5
13a	14	77.8	18	81.8	13	61.9	15	78.9	21	75.0	16	61.5
14a	4	22.2	8	36.4	3	14.3	6	31.6	7	25.0	5	19.2
15a	10	55.5	15	68.2	9	42.8	14	73.7	18	64.3	15	57.7
16a	8	44.4	11	50.0	7	33.3	11	57.9	15	53.6	12	46.1
17a	6	33.3	11	50.0	7	33.3	9	47.4	10	35.7	8	30.8
18a	13	72.2	17	77.3	10	47.6	15	78.9	20	71.4	16	61.5
19a	10	55.5	14	63.6	9	42.8	12	63.1	16	57.1	13	50.0
20a	3	16.7	6	27.3	NA	—	4	21.0	9	32.1	7	26.9
Gentamicin	18	100	22	100	21	100	19	100	—	—	—	—
Ketoconazole	—	—	—	—	—	—	—	—	28	100	26	100

The findings of the antimicrobial test revealed that most of the investigated compounds exhibited strong to moderate antibacterial effects towards the Gram-positive and Gram-negative bacteria. With regards to the antifungal activity, all the synthesized compounds exhibited good activities against both *Candida albicans* and *Aspergillus fumigatus* except 1a, 1b, and 12a.

The antimicrobial activities based on the main nuclei (pyrazole-based, pyridine-based, and pyrimidine-based moieties) showed the following results:

(a) Pyrazole-based moieties

• Dihydropyrazole derivatives were more active than pyrazole derivatives. The NH-dihydropyrazole (7a) showed very strong activities with all six microbes.

• However, *N*-substituted dihydropyrazole showed activities from very strong to weak depending on the type of substituent as follows:

(i) *N*-Substitution with CS-S-benzoyl like 18a got moderate activity with *E. coli* and very strong activity with the other five tested microbes.

(ii) While *N*-substitution with CS-S-aliphatic acid like 19a achieved moderate activities with all six microbes.

(iii) But other *N*-substituted dihydropyrazoles with CS-S-ester and CS-S-acetyl derivatives like 17a and 20a respectively showed weak activities with all the six tested microbes.

(iv) Moreover, *N*-substitution with CH_2_–CO acquired strong to moderate activities based on the type of C=O; if *N*-substituted with CH_2_–CO (ester) like 15a, it showed strong activities with *B. subtilis*, *P. aeuroginosa*, and *C. albicans*, and showed moderate activities with other tested microbes.

(v) While *N*-substitution with CH_2_–CO (amide) like 16a showed weak activity with *E. coli* and moderate activities with the other five microbes.

• On the other hand, all pyrazole derivatives showed weak activities with all tested microbes except *N*-substituted pyrazole with CS-NH_2_ like 10a and 10b achieved strong to moderate activities depending on the type of benzene derivative linked to position 3 of pyrazole:

(i) In the case of the activated ring like 4-hydroxyphenyl as in 10a, it showed moderate activities with all tested microbes.

(ii) While, in the case of the deactivated ring like 3,4-dichlorophenyl as in 10b, it showed very strong activity with *S. aureus*, *B. subtilis*, *P. aeuroginosa*, and *C. albicans* and moderate activities with other tested microbes.

(b) Pyridine-based moieties

• Pyridine derivatives which contain NH_2_ in the alpha position to N-heteroatom-like compounds 2a and 2b showed strong activities against *B. subtilis*, *P. aeuroginosa*, and *C. albicans* and got moderate activities with the other tested microbes.

• However, pyridine derivatives that contain C=O in the alpha position to N-heteroatom as in compounds 3a–6a showed weak activities against all tested microbes.

(c) Pyrimidine-based moieties

• Pyrimidine derivatives like 11a and 13a showed very strong to moderate activities depending on the type of substituent in position 2 of the pyrimidine ring, where:

(i) If position 2 contains a donating group like NH_2_ as in 11a, it showed very strong activity with *E. coli*, weak activity with *A. flavus*, and moderate activity with the other microbes.

(ii) While, when NH_2_ condensed with aromatic aldehyde as in compound 13a, it achieved very strong activities with all tested microbes.

### 2.3. *In silico* studies

#### 2.3.1. Molecular docking studies

To understand the pattern by which the investigated compounds bound to the active site, all the newly synthesized candidates were subjected to two different docking processes into the EGFR and VEGFR-2 binding sites. The affinities of the most active newly synthesized ligands (2a, 6a, 7a, 10b, 15a, and 18a) toward the target proteins (Table 3) were compared according to the docking score values calculated using the MOE 2019.0102.^42^

**Table 3:** Docking scores and RMSD values of the most biologically active compounds (2a, 6a, 7a, 10b, 15a, and 18a) compared to HYZ and GIG inhibitors of EGFER and VEGFER-2 active sites, respectively

Compound	EGFR		VEGFR-2	
	Docking score (kcal mol^−1^)	RMSD	Docking score (kcal mol^−1^)	RMSD
2a	−6.20	1.44	−6.08	1.57
6a	−6.52	0.81	−6.01	1.47
7a	−5.88	1.15	−5.15	2.13
10b	−6.45	1.42	−6.44	1.44
15a	−6.96	1.98	−6.17	1.23
18a	−7.47	1.86	−6.86	1.62
HYZ	−7.81	0.99	—	—
GIG	—	—	−9.54	1.29

First, two validation processes were carried out to confirm the validity of the MOE program in getting accurate docking results. This was established by redocking each one of the two co-crystallized inhibitors (HYZ and GIG) of EGFR and VEGFR-2 receptors, respectively. The obtained low values of RMSD (0.99 and 1.29 Å, respectively) which represent the root mean square deviation between the native and redocked poses of the co-crystallized inhibitor ensured the valid performance.^43,44^ Besides, the most active dual EGFR/VEGFR-2 inhibitors (2a and 10b) were selected for further investigations compared to the native inhibitors (HYZ and GIG) of EGFR and VEGFR-2, respectively (Table 4).

**Table 4:** 2D and 3D pictures representing the interaction of compounds (2a and 10b) with EGFR and VEGFR-2 binding pockets, besides the docked co-crystallized inhibitors (HYZ and GIG)

Compound	Receptor	3D interactions	3D positioning
2a	EGFR	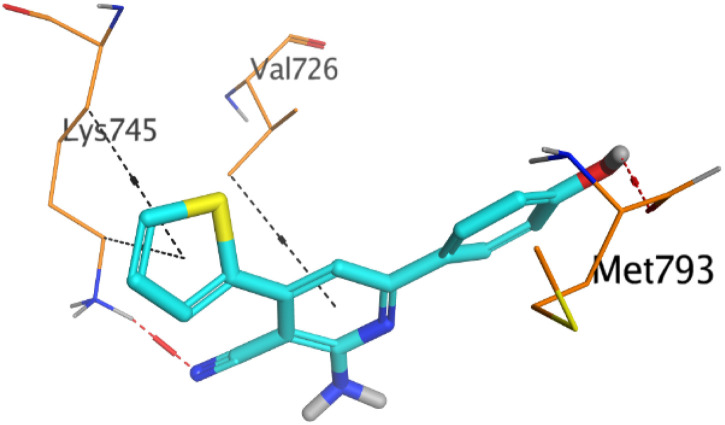	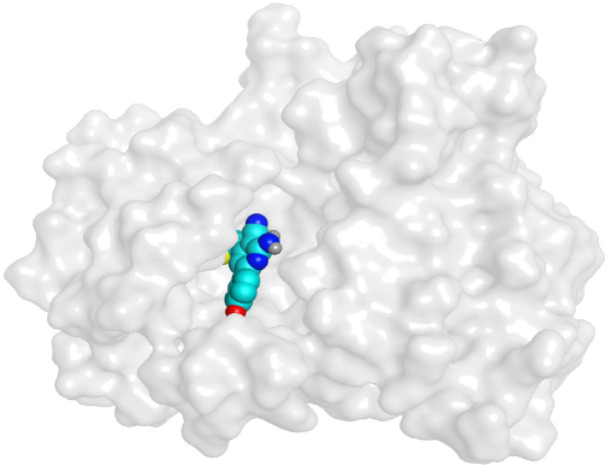
	VEGFR-2	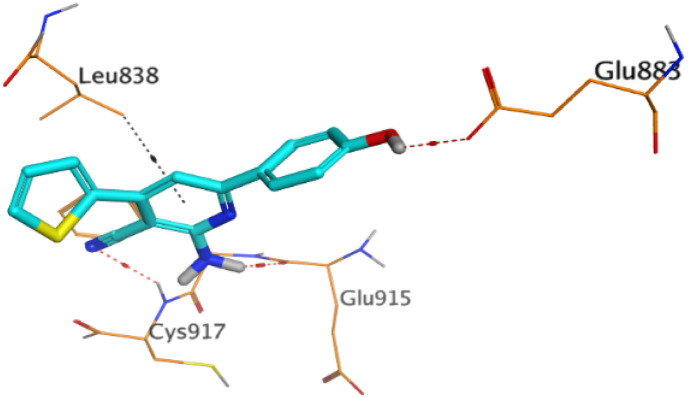	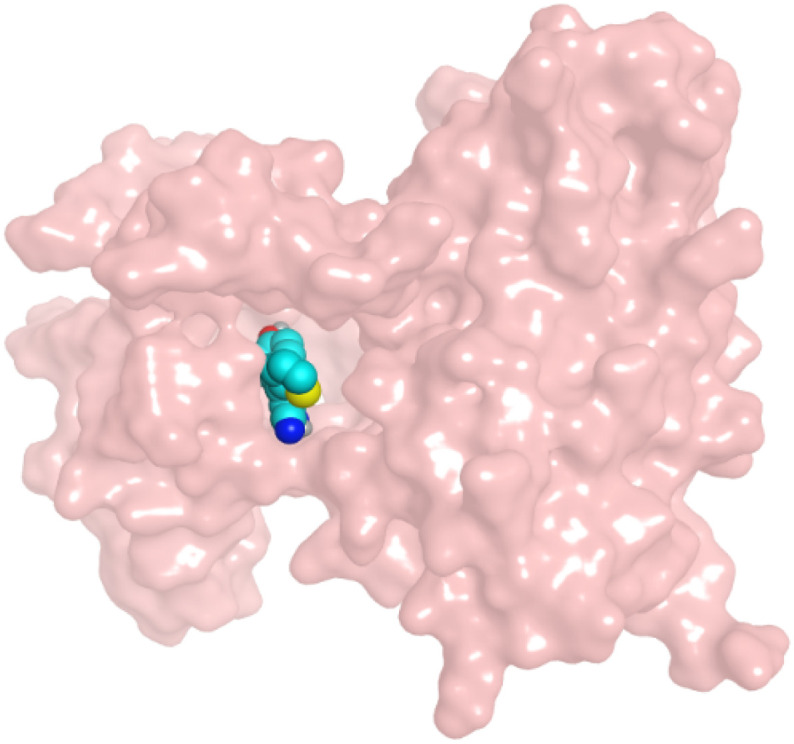
10b	EGFR	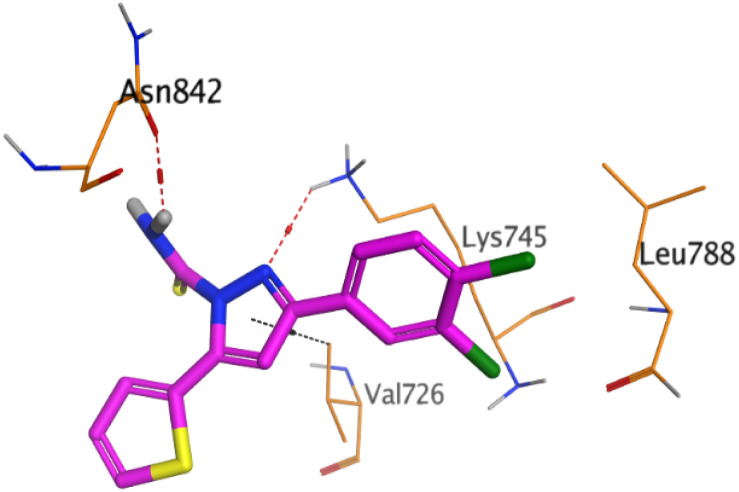	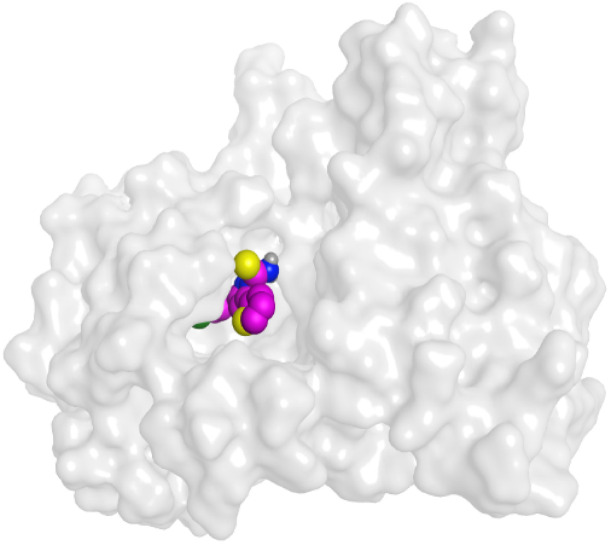
	VEGFR-2	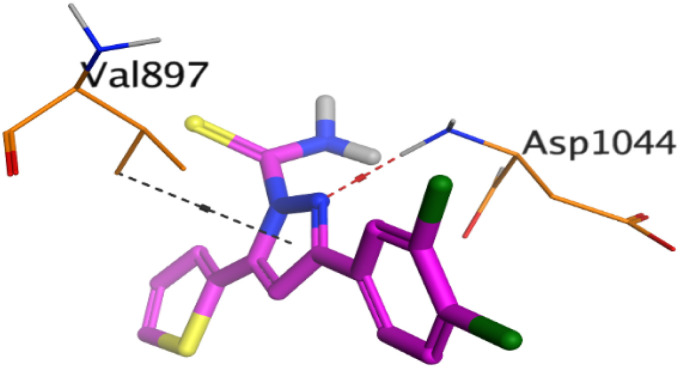	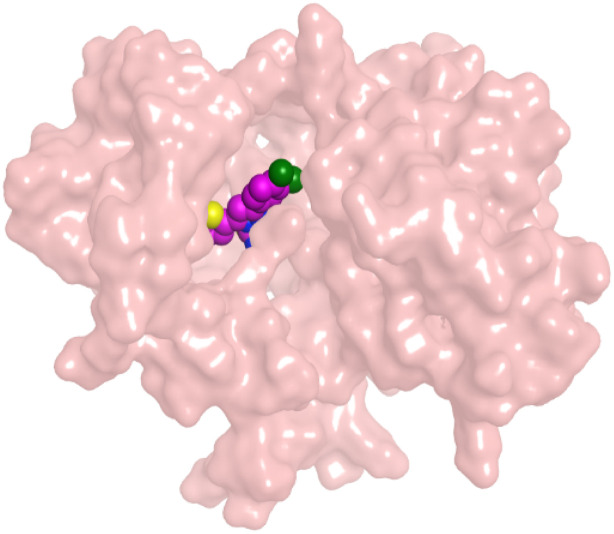
HYZ	EGFR	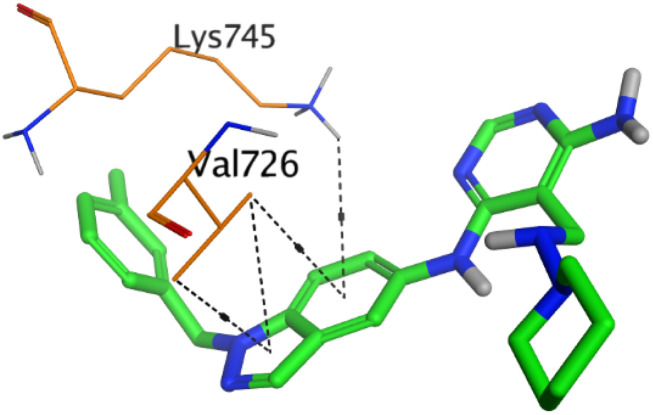	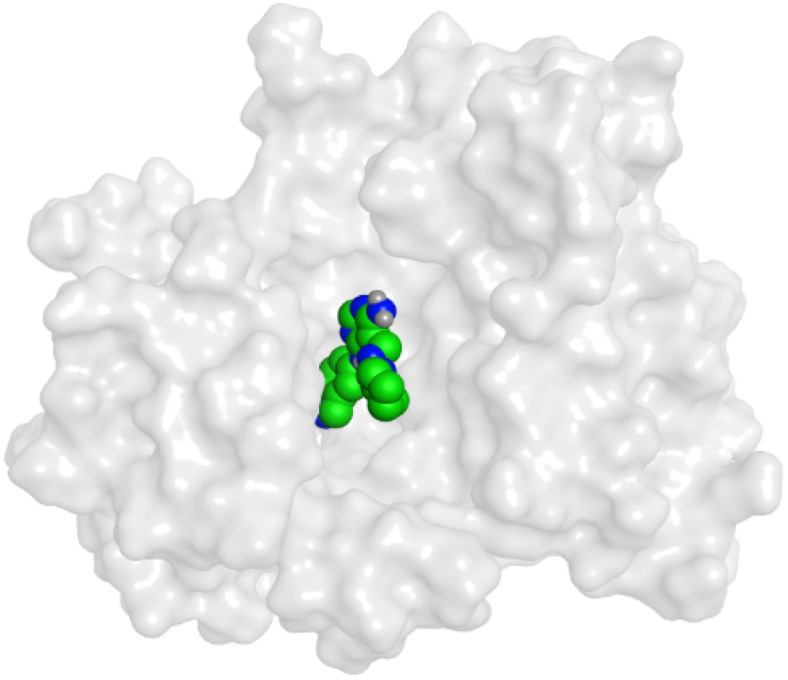
GIG	VEGFR-2	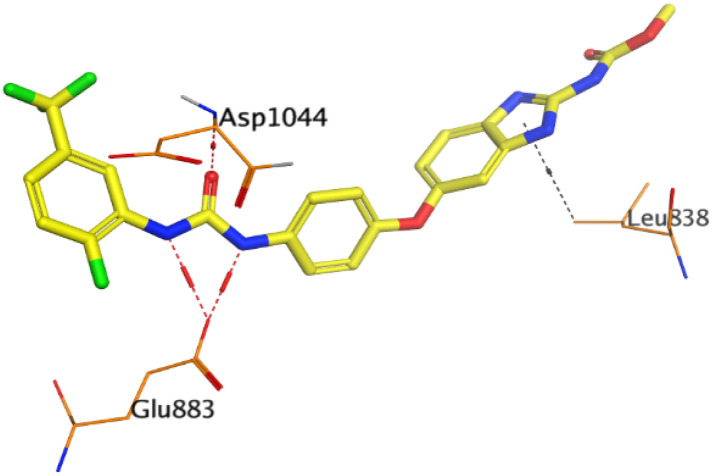	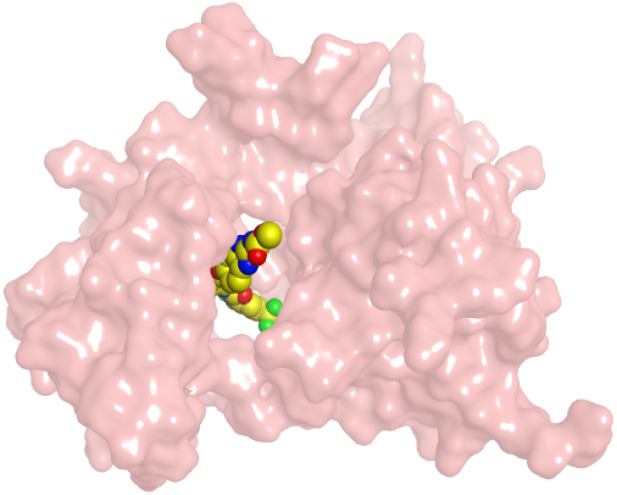

HYZ inside the binding pocket of the EGFR receptor (*S* = −7.80 kcal mol^−1^ and RMSD = 1.33 Å) was found to form one pi–cation bond with LYS745 at 4.16 Å and two pi–H bonds with VAL726 at 4.29 and 4.64 Å, respectively. However, GIG inside the binding pocket of the VEGFR-2 receptor (*S* = −9.54 kcal mol^−1^ and RMSD = 1.29 Å) formed two H-bonds with GLU833 at 2.90 and 2.79 Å, respectively, one H-bond with ASP1044 at 2.95 Å, and one pi–H bond with LEU838 at 4.04 Å.

However, compound 2a inside the binding pocket of the EGFR receptor (*S* = −6.20 kcal mol^−1^ and RMSD = 1.44 Å) was found to form two H-bonds with MET793 and LYS745 amino acids at 3.16 and 3.74 Å, respectively. Besides, it formed two pi–H bonds with LYS745 at 4.51 and 4.60 Å, and one pi–H bond with VAL726 at 3.77 Å. On the other hand, its binding score within the active site of the VEGFR-2 receptor was −6.08 kcal mol^−1^ (RMSD = 1.58 Å). It showed the formation of three H-bonds with GLU883, GLU915, and CYS917 at 3.04, 2.80, and 3.65 Å, respectively. It also bound LEU 838 with a pi–H bond at 3.65 Å.

On the other hand, compound 10b inside the binding pocket of the EGFR receptor (*S* = −6.45 kcal mol^−1^ and RMSD = 1.42 Å) formed three H-bonds with ASN842, LEU788, and LYS745 amino acids at 3.12, 3.22, and 3.52 Å, respectively. Moreover, it formed a pi–H bond with VAL726 at 3.82 Å. Furthermore, it showed the formation of one H-bond with ASP1044 at 2.92 Å and one pi–H bond with VAL896 at 3.73 Å, within the active site of the VEGFR-2 receptor (*S* = −6.44 kcal mol^−1^ and RMSD = 1.44 Å).

#### 2.3.2. DFT calculations

From the experimental biological activity, compound 10b showed very strong anti-proliferative activity against the two enzymes (EGFER and VEGFER-2) with IC_50_ values of 0.161 and 0.141 μM, respectively. The reference drug of EGFER (erlotinib) showed an IC_50_ of 0.037 μM while the reference drugs of VEGFER-2 (sorafenib) showed an IC_50_ value of 0.034 μM.

**Fig. 7:**
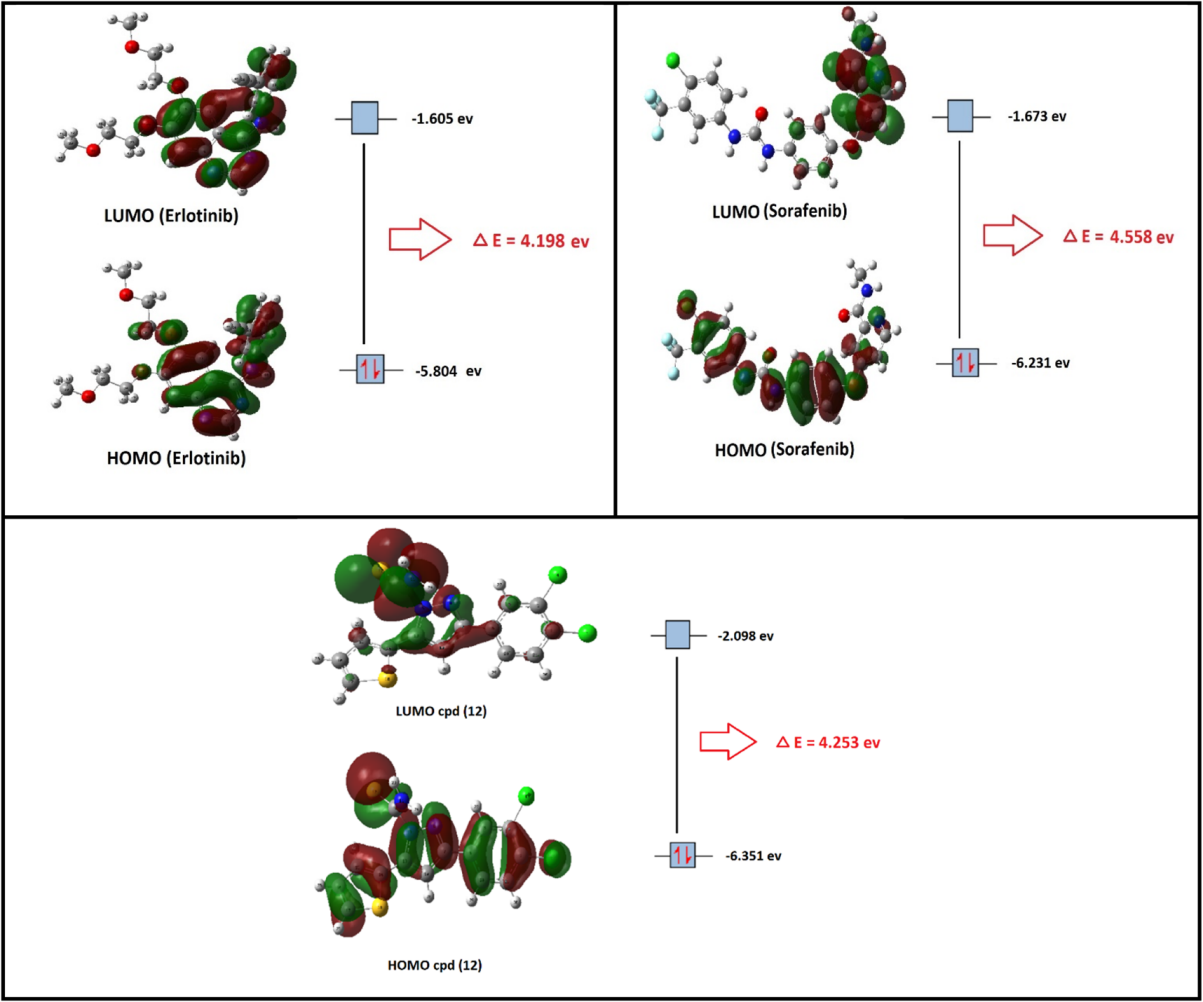
The optimized structures and HOMO–LUMO charge density maps of compound 10b and the reference drugs (erlotinib and sorafenib) were calculated at the B3LYP-6-31G+(d,p) level of theory.

In the present section, we tried to correlate compound 10b and the reference drugs, erlotinib, and sorafenib. Density functional theory (DFT) calculations were performed by using the Gaussian 09W program.^45^ The optimized structures of 10b, erlotinib, and sorafenib are represented in Fig. 7.

Moreover, the LUMO energy characterizes the sensitivity of the molecule to a nucleophilic attack, and the HOMO energy characterizes the susceptibility of a molecule to an electrophilic attack. Electronegativity (*χ*) is the parameter that reflects the ability of a molecule not to let out its electrons. Global softness (*S*) expresses the resistance of a system to the change in its number of electrons. The theoretical global parameters calculated at the same level of theory are listed in Table 5. The energy gap (*E*_g_) between HOMO and LUMO characterizes the molecular chemical stability and molecular electrical transport properties because it is a measure of electron conductivity.

**Table 5:** Total energy, the energy of HOMO and LUMO, energy gap, dipole moment, ionization potential (*I*, eV), electron affinity (*A*, eV), chemical hardness (*η*, eV), global softness (*S*, eV^−1^), chemical potential (*p*, eV^−1^), and electronegativity (*χ*, eV) of compound 10b and the reference drugs (erlotinib and sorafenib) computed at the B3LYP/6-311+G(d,p) level of theory

Property	Compound		
	10b	Erlotinib	Sorafenib
*E*_HOMO_ (eV)	−6.351	−5.804	−6.231
*E*_LUMO_ (eV)	−2.098	−1.605	−1.673
Energy gap = |*E*_HOMO_ − *E*_LUMO_| eV	4.253	4.198	4.558
Ionization potential (*I* = −*E*_HOMO_) eV	6.351	5.804	6.231
Electron affinity (*A* = −*E*_LUMO_) eV	2.098	1.605	1.673
Electronegativity *χ* = (*I* + *A*)/2 eV	4.224	3.704	3.952
Chemical potential *p* = −*χ* eV	−4.224	−3.704	−3.952
Chemical hardness (*η* = (*I* − *A*)/2) eV	2.127	2.099	2.279
Chemical softness (*S* = 1/2*η*) eV	0.235	0.238	0.219

From the data in Table 5, the order of increasing *E*_HOMO_ is 10b > sorafenib > erlotinib while the order of decreasing *E*_LUMO_ is erlotinib < sorafenib < 10b. From the order of *E*_HOMO_ and *E*_LUMO_, compound 10b has the highest nucleophilicity and electrophilicity.

Also, analyzing the calculated values of hardness (*η*) and softness (*S*) for the investigated compounds revealed the donor–acceptor behavior of the studied molecules. Back to the energies of HOMO and LUMO, the order of increasing the energy gap is sorafenib > 10b > erlotinib, so sorafenib is the hardest one (large energy gap) while erlotinib (small energy gap) represents the softest molecule (*c.f.*Fig. 7). Hence, the most polarizable (softest) with easier charge transfer and highest chemical reactivity molecule is erlotinib with *S* = 0.238 eV, while the least polarizable molecule (hardest) is sorafenib with *η* = 2.279 eV.

Moreover, the ionization potential (*I*) eV which is also related to the donating properties of the molecules (the higher the ionization potential value the lower the donation ability of the molecule) was used to evaluate the tendency of molecules to be oxidized. It showed that the order of increasing of the most probable molecule to act as a reducing agent is erlotinib < sorafenib < 10b.

Furthermore, the escaping tendency of an electron is measured by its chemical potential *p* (eV) and it is also related to its electronegativity. As *p* increases, the tendency of a molecule to lose an electron increases. Electronegativity (*χ*) represents the molecular ability to attract electrons, the (*χ*) values displayed in Table 5 show that 10b > sorafenib > erlotinib in electronegativity and erlotinib > sorafenib > 10b in chemical potential (*P*).

The chemical hardness and chemical potential are the main factors of the overall reactivity of the molecule and are the most fundamental descriptors of charge transfer during a chemical reaction.

### 2.4. Structure–activity relationship (SAR)

**Fig. 8:**
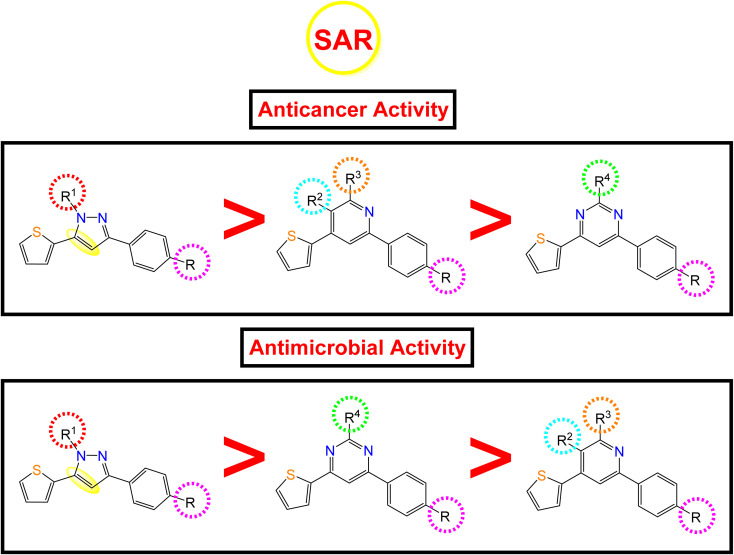
Anticancer and antimicrobial activities of the novel pyrazole, pyridine, and pyrimidine candidates related to their chemical structures.

Following the biological studies of the new pyrazole, pyridine, and pyrimidine derivatives as anticancer and antimicrobial candidates, we can conclude the following interesting results (Fig. 8):

Generally, the 4-OH substitution on the side phenyl ring was found to be superior to the 3,4-diCl substitution regarding both the anticancer and antimicrobial activities.

• Regarding the anticancer activity: the descending order of activity was found to be (dihydropyrazole > *N*-substituted dihydropyrazole > pyridine > pyrimidine derivatives).

(i) Compound 7a (R1 = –H) with dihydropyrazole nucleus was found to be the most active against both HepG-2 and MCF-7 cancer cell lines.

(ii) However, the *N*-substituted dihydropyrazole derivatives achieved very strong to moderate antitumor activities according to the R^1^ substitution as follows: –CH_2_COOC_2_H_5_ (15a) > –CH_2_CONHphSO_2_NH_2_ (16a) > –CSSCOph (18a) > –CSSCH_2_COOH (19a) > –CSSCH_2_COOC_2_H_5_ (17a) > –CSSCOCH_2_Cl (20a) > –CSNH_2_ (10a, 10b).

(iii) Besides, the compounds containing the pyridine nucleus exhibited lower activity than the dihydropyrazole and *N*-substituted dihydropyrazole nuclei. The pyridine candidates showed very strong to moderate anticancer activities according to both R^2^ and R^3^ substituents as follows: –CN and –NH_2_ (2a) > fused pyridopyridine (6a) > –COCH_3_ and –CO (3a) ≡ –COOC_2_H_5_ and CO (4a) ≡ –CN and –OH (5a), respectively.

(iv) Moreover, the pyrimidine moiety derivatives were the lowest anticancer with moderate to weak activities regarding the R^4^ substituent as follow: –NH_2_ (11a) > –NCHphOH (13a).

• Regarding the antimicrobial activity: the descending order of activity was noted to be (dihydropyrazole > *N*-substituted dihydropyrazole > pyrimidine > pyridine derivatives).

(i) Again, compound 7a (R1 = –H) with dihydropyrazole nucleus exhibited superior antibacterial and antifungal activities.

(ii) On the other hand, the *N*-substituted dihydropyrazole derivatives achieved strong to moderate antimicrobial activities according to the R^1^ substitution as follows: –CSSCOph (18a) > –CH_2_COOC_2_H_5_ (15a) > –CSSCH_2_COOH (19a) > –CSNH_2_ (2,3-diClph) (10b) > –CSNH_2_ (4-OHph) (10a) > –CH_2_CONHphSO_2_NH_2_ (16a) > –CSSCH_2_COOC_2_H_5_ (17a) > –CSSCOCH_2_Cl (20a).

(iii) Controversy, the pyrimidine moiety derivatives showed better antimicrobial activities than the pyridine ones. The order of decreasing the antimicrobial potentials was according to the R^4^ substituent as follows: –NCHphOH (13a) > –NH_2_ (11a).

(iv) Furthermore, the pyridine-containing compounds exhibited lower antimicrobial activities according to both R^2^ and R^3^ substituents as follows: –CN and –NH_2_ (4-OHph) (2a) > –CN and –NH_2_ (2,3-diClph) (2b) > –CN and –OH (5a) > –COOC_2_H_5_ and CO (4a) > fused pyridopyridine (6a) > –COCH_3_ and –CO (3a).

## 3. Conclusion

Guided by the basic pharmacophoric requirements of both EGFR and VEGFR-2 inhibitors, novel twenty-two 4-thiophenyl-pyrazole, pyridine, and pyrimidine candidates were designed as potential dual EGFR/VEGFR-2 inhibitors. On the other hand, their antibacterial and antifungal activities were screened. First, the SAR studies revealed that the descending order of anticancer activity was according to (dihydropyrazole > *N*-substituted dihydropyrazole > pyridine > pyrimidine derivatives). The anticancer activities of compounds 2a, 6a, 7a, 10b, 15a, and 18a were found to be very strong to strong against both HepG-2 and MCF-7. These candidates were further evaluated against both EGFR and VEGFR-2 targets, compared to erlotinib and sorafenib, respectively. Both 10b and 2a derivatives achieved better dual EGFR/VEGFR-2 inhibition with IC_50_ values of (0.161 and 0.141 μM) and (0.209 and 0.195 μM), respectively. Moreover, compound 10b showed a marked decline in cell populations at the S phase with 49.68% (1.37-fold) compared to that of the control (36.19%). Also, the treatment of HepG-2 with the IC_50_ value of 10b revealed significant elevations in the percentage of the AnxV-FITC apoptotic cells in both the early (from 0.36 to 14.52%) and late (from 0.21 to 24.45%) apoptotic phases, compared to the control. Accordingly, we can confirm the exact mechanism of the anticancer activity of 10b to be due to apoptosis. On the other hand, screening the new compounds against Gram-positive bacteria, Gram-negative bacteria, and fungi revealed that most of them exhibited strong to moderate antibacterial and antifungal potentials. SAR studies showed that the descending order of antimicrobial activity was noted to be (dihydropyrazole > *N*-substituted dihydropyrazole > pyrimidine > pyridine derivatives). Collectively, compound 7a with dihydropyrazole nucleus was found to be the most active against both HepG-2 and MCF-7 cancer cell lines and exhibited superior antibacterial and antifungal activities as well. Furthermore, both 2a and 10b showed greatly similar binding scores and modes regarding the co-crystallized inhibitors of EGFR and VEGFR-2 binding sites. Finally, the DFT calculations described that compound 10b has the highest nucleophilicity and electrophilicity and the order of increasing the energy gap is sorafenib > 10b > erlotinib, so sorafenib is the hardest one followed by 10b.

## 4. Experimental section

### 4.1. Chemistry

All melting points were measured on a Gallen Kamp melting point apparatus (Sanyo Gallen Kamp, UK) and were uncorrected. The microwave reactions were done by Microsynth instrument type MA143 (micro wave flux). The ultrasound-assisted reactions were performed in Digital Ultrasonic Cleaner CD-4830 (35 kHz, 310 W). The IR spectra were recorded on a Pye-Unicam SP-3-300 infrared spectrophotometer (KBr dicks) and expressed in wave number (cm^−1^). ^1^H NMR spectra were run at 300 and 400 MHz, on a Varian Mercury VX-300 and Bruker Avance III NMR spectrometer, respectively. TMS was used as an internal standard in deuterated dimethylsulphoxide (DMSO-d6). Chemical shifts (*δ*) are quoted in ppm. The abbreviations used are as follows: s, singlet; d, doublet; m, multiplet. All coupling constant (*J*) values are given in hertz. Elemental analyses were performed on the CHN analyzer and all compounds were within ±0.4 of the theoretical values. The reactions were monitored by thin-layer chromatography (TLC) using TLC sheets coated with UV fluorescent silica gel Merck 60 F254 plates and were visualized using a UV lamp and different solvents as mobile phases. All reagents and solvents were purified and dried by standard techniques. Compound 1a was prepared according to the previously reported methodology.^46^

#### 4.1.1. Formation of (*E*)-1-(4-hydroxyphenyl)-3-(thiophen-2-yl)prop-2-en-1-one (1a)

A mixture of 4-hydroxy acetophenone (1.36 g; 0.01 mol) and thiophene-2-carboxaldehyde (1.12 mL; 0.01 mol) in methanol (6 mL) and 10% sodium hydroxide solution (10 mL) was stirred at room temperature for 12 h. The reaction mixture was acidified with diluted hydrochloric acid the resulting solid was filtered out, dried, and recrystallized to produce compound 1a.

#### 4.1.2. Formation of (*E*)-1-(3,4-dichlorophenyl)-3-(thiophen-2-yl)prop-2-en-1-one (1b)

A solution of 3,4-dichloro acetophenone (1.89 g; 0.01 mol) and thiophene-2-carboxaldehyde (1.12 mL; 0.01 mol) in ethanolic sodium hydroxide solution (20 mL) was stirred in an ice bath for 3 h. The obtained solid was filtered off and collected after recrystallization to give compound 1b.

Yield 55%; as yellow crystal; mp 85 °C (EtOH); IR (cm^−1^) *ν*: 1655 (C=O); ^1^H-NMR: 7.21 (d, d, 1H, CH thiophene ring), 7.57 (d, 1H, CH-olefinic), 7.73 (d, 1H, CH of benzene ring), 7.81 (s, 1H, CH benzene ring), 7.82 (d, 1H, CH of benzene ring), 7.92 (d, 1H, CH olefinic), 8.04 (d, 1H, CH of thiophene ring), 8.07 (d, 1H, CH of thiophene ring); MS (*m*/*z*) (%): 283 (M+, 15), 117 (100). Anal. calcd for C_13_H_8_Cl_2_OS (283.17): C, 55.14; H, 2.85; found: C, 54.89; H, 2.74.

#### 4.1.3. General procedure for the synthesis of compounds (2a) and (2b)

A mixture of chalcone 1a (2.30 g; 0.01 mol) and/or chalcone (1b) (2.83 g; 0.01 mol), malononitrile (0.66 g; 0.01 mol) and ammonium acetate (2.31 g; 0.03 mol) was refluxed for 6–8 h in ethanol (20 mL). The reaction mixture was cooled and poured onto crushed ice, and the obtained solid was filtered off, dried, and recrystallized to give compounds 2a and 2b, respectively.

##### 4.1.3.1. 2-Amino-6-(4-hydroxyphenyl)-4-(thiophen-2-yl)nicotinonitrile (2a)

Yield 83%; orange crystal; mp 260 °C (EtOH); IR (cm^−1^) *ν*: 3417 (OH), 3357, 3223 (NH_2_), 2200 (CN), 1617 (C=N); ^1^H-NMR: 6.86–8.00 (m, 7H, Ar-H), 7.15 (s, 2H, NH_2_), 7.84 (s, 1H, CH of pyridine), 9.96 (s, 1H, OH). Anal. calcd for C_16_H_11_N_3_OS (293.34): C, 65.51; H, 3.78; N, 14.32; found: C, 65.36; H, 3.70; N, 14.21.

##### 4.1.3.2. 2-Amino-6-(3,4-dichlorophenyl)-4-(thiophen-2-yl)nicotinonitrile (2b)

Yield 63%; black crystal; mp 200 °C (EtOH); IR (cm^−1^) *ν*: 3362, 3238 (NH_2_), 2213 (CN), 1640 (C=N); ^1^H-NMR: 6.91 (s, 2H, NH_2_), 6.94 (s, 1H, CH of pyridine ring), 7.19–7.92 (m, 6H, Ar-H). Anal. calcd for C_16_H_9_Cl_2_N_3_S (346.23): C, 55.51; H, 2.62; N, 12.14; found: C, 55.34; H, 2.54; N, 12.21.

#### 4.1.4. General procedure for the synthesis of compounds (3a) and (4a)

A mixture of 1a (2.30 g; 0.01 mol) and/or ethyl acetoacetate (1.30 mL; 0.01 mol) and diethyl malonate (1.60 mL; 0.01 mol) in presence of ammonium acetate (2.31 g; 0.03 mol) was refluxed for 8–10 h in ethanol (20 mL). After cooling, the reaction mixture was poured over ice, the obtained precipitate was filtered out, dried, and recrystallized to give 3a and 4a, respectively.

##### 4.1.4.1. 3-Acetyl-6-(4-hydroxyphenyl)-4-(thiophen-2-yl)pyridin-2(1*H*)-one (3a)

Yield 75%; brown crystal; mp 220 °C (EtOH) IR (cm^−1^) *ν*: 3331 (OH), 3107 (NH), 1695 (C=O); ^1^H-NMR: 2.50 (s, 3H, CH_3_–CO), 6.87–8.03 (m, 7H, Ar-H), 7.77 (s, 1H, CH pyridine ring), 9.86 (s, 1H, OH); MS (*m*/*z*) (%): 311 (M+, 20), 289 (100). Anal. calcd for C_17_H_13_NO_3_S (311.36): C, 65.58; H, 4.21; N, 4.50; found: C, 65.45; H, 4.14; N, 4.42.

##### 4.1.4.2. Ethyl 6-(4-hydroxyphenyl)-2-oxo-4-(thiophen-2-yl)-1,2-dihydropyridine-3-carboxylate (4a)

Yield 83%; brown crystal; mp 125 °C (EtOH); IR (cm^−1^) *ν*: 3170 (NH), 1725 (CO), 1675 (CO); ^1^H-NMR: 1.11 (t, 3H, CH_3_), 3.91 (q, 2H, CH_2_), 6.79–8.01 (m, 7H, Ar-H), 7.19 (s, 1H, NH), 7.81 (s, 1H, CH pyridine ring), 9.95 (s, 1H, OH). Anal. calcd for C_18_H_15_NO_4_S (341.38): C, 63.33; H, 4.43; N, 4.10; found: C, 63.10; H, 4.32; N, 4.00.

#### 4.1.5. Formation of 2-hydroxy-6-(4-hydroxyphenyl)-4-(thiophen-2-yl)nicotinonitrile (5a)

A mixture of 1a (2.30 g; 0.01 mol) and ethyl cyanoacetate (1.13 mL; 0.01 mol) in presence of ammonium acetate (2.31 g; 0.03 mol) was refluxed for 6–8 h in ethanol (20 mL). The reaction mixture was cooled and poured onto crushed ice/dilute hydrochloric acid and the formed product was filtered off, dried, and recrystallized to afford compound 5a.

Yield 67%; yellow crystal; mp 200 °C (EtOH); IR (cm^−1^) *ν*: 3400 (OH), 2216 (CN), 1636 (C=N); ^1^H-NMR: 6.81–8.01 (m, 8H, Ar-H), 10.22 (s, 1H, OH of benzene ring), 12.48 (s, 1H, OH of pyridine ring). Anal. calcd for C_16_H_10_N_2_O_2_S (294.33): C, 65.29; H, 3.42; N, 9.52; found: C, 65.18; H, 3.48; N, 9.60.

#### 4.1.6. Formation of 4-amino-7-(4-hydroxyphenyl)-2-oxo-5-(thiophen-2-yl)-1,2-dihydro-1,8-naphthyridine-3-carbonitrile (6a)

##### 4.1.6.1. Conventional method

A mixture of chalcone 1a (2.30 g; 0.01 mol), ethyl cyanoacetate (1.13 mL; 0.01 mol), and ammonium acetate (2.31 g; 0.03 mol) in ethanol (20 mL) was refluxed for 6–8 h, followed by addition of ethyl cyanoacetate (1.13 mL; 0.01 mol) and ammonium acetate (2.31 g; 0.03 mol) in ethanol (20 mL) with 6–8 h of continuous refluxing. The reaction mixture was cooled and poured onto crushed ice/dilute hydrochloric acid and the formed product was filtered off, dried, and recrystallized to get compound 6a.

##### 4.1.6.2. Microwave method

A mixture of 1a (2.30 g; 0.01 mol), ethyl cyanoacetate (2.26 mL; 0.02 mol), and ammonium acetate (2.31 g; 0.03 mol) in DMF (20 mL) was synthesized under microwave irradiation at 400 W for 6 min. The reaction mixture was poured onto crushed ice. The formed solid was filtered off, dried, and recrystallized to afford compound 6a.

Yield 77%; yellow crystal; mp 260 °C (EtOH); IR (cm^−1^) *ν*: 3480 (OH), 3369 (NH), 2217 (CN), 1683 (C=O), 1649 (C=N); ^1^H-NMR: 6.47 (s, 2H, NH_2_), 6.84–8.05 (m, 7H, Ar-H), 7.07 (s, 1H, CH of pyridine ring), 9.81 (s, 1H, NH), 12.51 (s, 1H, OH). Anal. calcd for C_19_H_12_N_4_O_2_S (360.39): C, 63.32; H, 3.36; N, 15.55; found: C, 63.09; H, 3.40; N, 15.48.

#### 4.1.7. Formation of 4-(5-(thiophen-2-yl)-4,5-dihydro-1*H*-pyrazol-3-yl)phenol (7a)

A mixture of chalcone 1a (2.30 g; 0.01 mol) and hydrazine hydrate (2 mL) was refluxed for 8–10 h in ethanol (20 mL). After cooling, the reaction mixture was poured onto an ice bath, and the obtained precipitate was filtered out, dried, and recrystallized to get compound 7a.

Yield 83%; white crystal; mp 100–102 °C (EtOH); IR (cm^−1^) *ν*: 3290 (NH), 2879 (CH aliphatic), 1606 (C=N); ^1^H-NMR: 2.82 (d, d, 2H, CH_2_ of pyrazole ring), 5.05 (d, d, 1H, CH of pyrazole ring), 6.77–7.47 (m, 7H, Ar-H), 9.67 (s, 1H, NH). Anal. calcd for C_13_H_12_N_2_OS (244.31): C, 63.91; H, 4.95; N, 11.47; found: C, 63.72; H, 4.84; N, 11.36.

#### 4.1.8. Formation of 1-(3-(3,4-dichlorophenyl)-5-(thiophen-2-yl)-4,5-dihydro-1*H*-pyrazol-1-yl)ethan-1-one (8b)

A mixture of 1b (2.83 g; 0.01 mol) and hydrazine hydrate (2 mL) was refluxed for 3 h in presence of glacial acetic acid (15 mL). The obtained precipitate was cooled and poured onto crushed ice, filtered off, dried, and recrystallized to afford compound 8b.

Yield 75%; brown crystal; mp 160 °C (EtOH); IR (cm^−1^) *ν*: 2926 (CH-aliphatic), 1667 (CO), 1636 (C=N); ^1^H-NMR: 2.48 (s, 3H, CH_3_–CO), 3.27 (d, d, 2H, CH_2_ of pyrazole ring), 5.84 (d, d, 1H, CH of pyrazole ring), 6.91–7.99 (m, 6H, Ar-H). Anal. calcd for C_15_H_12_Cl_2_N_2_OS (339.23): C, 53.11; H, 3.57; N, 8.26; found: C, 52.89; H, 3.50; N, 8.35.

#### 4.1.9. Formation of 3-(3,4-dichlorophenyl)-1-phenyl-5-(thiophen-2-yl)-4,5-dihydro-1*H*-pyrazole (9b)

A mixture of 1b (2.83 g; 0.01 mol) and phenylhydrazine (1.08 g; 0.01 mol) was refluxed for 3 h in presence of ethanol (20 mL). Leave to cool, the solid obtained was filtered off, dried, and recrystallized from a suitable solvent to give compound 9b.

Yield 81%; beige crystal; mp 130 °C (EtOH); IR (cm^−1^) *ν*: 2867 (CH aliphatic), 1593 (C=N); NMR: 3.24 (d, d, 2H, CH_2_ of pyrazole ring), 5.86 (d, 1H, CH of pyrazole ring), 6.75–7.93 (m, 11H, Ar-H). Anal. calcd for C_19_H_14_Cl_2_N_2_S (373.30): C, 61.13; H, 3.78; N, 7.50; found: C, 60.87; H, 3.66; N, 7.43.

#### 4.1.10. Formation of 3-(4-hydroxyphenyl)-5-(thiophen-2-yl)-1*H*-pyrazole-1-carbothioamide (10a)

A mixture of 1a (2.30 g; 0.01 mol) and thiosemicarbazide (0.91 g; 0.01 mol) was refluxed for 18 h in presence of glacial acetic acid (5 mL) and ethanol (20 mL). The obtained precipitate was cooled and poured onto crushed ice, filtered off, dried, and recrystallized to get compound 10a.

Yield 73%; green crystal; mp 195–197 °C (EtOH); IR (cm^−1^) *ν*: 3406 (OH), 3343, 3268 (NH_2_), 2921 (CH aliphatic), 1636 (C=N); ^1^H-NMR: 7.37–8.51 (m, 7H, Ar-H), 6.88 (s, 1H, CH of pyrazole ring), 10.03 (s, 2H, NH_2_), 10.42 (s, 1H, OH). Anal. calcd for C_14_H_11_N_3_OS_2_ (301.38): C, 55.79; H, 3.68; N, 13.94; found: C, 55.58; H, 3.72; N, 14.05.

#### 4.1.11. Formation of 3-(3,4-dichlorophenyl)-5-(thiophen-2-yl)-1*H*-pyrazole-1-carbothioamide (10b)

A mixture of 1b (2.83 g; 0.01 mol) and thiosemicarbazide (0.91 g; 0.01 mol) in ethanolic sodium hydroxide solution (20 mL) was refluxed for 3 h. The reaction mixture was cooled, and acidified with diluted hydrochloric acid and the resulting solid was filtered out, dried, and recrystallized to afford compound 10b.

Yield 65%; bale yellow crystal; mp 260 °C (EtOH); IR (cm^−1^) *ν*: 3259, 3145 (NH_2_), 1639 (C=N), 1596 (C=C), 1297 (C=S); ^1^H-NMR: 6.22–8.17 (m, 6H, Ar-H), 6.99 (s, 1H, CH of pyrazole ring), 7.73 (s, 2H, NH_2_); MS (*m*/*z*) (%): 354 (M+, 10), 109 (100). Anal. calcd for C_14_H_9_Cl_2_N_3_S_2_ (354.27): C, 47.47; H, 2.56; N, 11.86; found: C, 47.30; H, 2.49; N, 11.76.

#### 4.1.12. Formation of 4-(2-amino-6-(thiophen-2-yl)pyrimidin-4-yl)phenol (11a)

A mixture of chalcone 1a (2.30 g; 0.01 mol) and guanidine hydrochloride (0.95 g; 0.01 mol) in ethanolic sodium hydroxide solution (20 mL) was refluxed for 8 h. The reaction mixture was cooled, and acidified with diluted hydrochloric acid and the resulting solid was filtered out, dried, and recrystallized to afford compound 11a.

Yield 76%; yellow crystal; mp 180 °C (EtOH); IR (cm^−1^) *ν*: 3290 (OH), 3200 (NH_2_), 1636 (C=N); ^1^H-NMR: 6.91 (s, 2H, NH_2_), 6.89–8.01 (m, 7H, Ar-H), 7.65 (s, 1H, CH of pyrimidine ring), 10.43 (s, 1H, OH); MS (*m*/*z*) (%): 269 (M+, 10), 74 (100). Anal. calcd for C_14_H_11_N_3_OS (269.32): C, 62.44; H, 4.12; N, 15.60; found: C, 62.23; H, 4.19; N, 15.51.

#### 4.1.13. Formation of 4-(1-(thiazolo[4,5-*b*]quinoxalin-2-yl)-5-(thiophen-2-yl)-1*H*-pyrazol-3-yl)phenol (12a)

A solution of 10a (3.03 g; 0.01 mol) and 2,3-dichloroquinoxaline (1.99 g; 0.01 mol) was refluxed for 15 h in presence of glacial acetic acid (5 mL) and absolute ethanol (20 mL). The obtained solid was filtered out, dried, and recrystallized to get compound 12a.

Yield 78%; brown crystal; mp 250–252 °C (EtOH); IR (cm^−1^) *ν*: 3444 (OH), 1664 (C=N); ^1^H-NMR: 6.90–7.98 (m, 11H, Ar-H), 7.17 (s, 1H, CH of pyrazole ring), 9.00 (s, 1H, OH). Anal. calcd for C_22_H_13_N_5_OS_2_ (427.50): C, 61.81; H, 3.07; N, 16.38; found: C, 61.69; H, 3.00; N, 16.26.

#### 4.1.14. Formation of compound 3-(3,4-dichlorophenyl)-*N*-(2-oxo-2-phenylethyl)-5-(thiophen-2-yl)-1*H*-pyrazole-1-carbothioamide (12b)

A solution of 10b (3.54 g; 0.01 mol) and phenacyl bromide (1.99 mL; 0.01 mol) was refluxed for 7–8 h in presence of acetic anhydride (20 mL), acetic acid (10 mL) and sodium acetate anhydrous (2 g). The obtained precipitate was cooled and poured onto crushed ice, filtered off, dried, and recrystallized to get compound 12b.

Yield 63%; green crystal; mp 160 °C (EtOH); IR (cm^−1^) *ν*: 3112 (NH), 2849 (CH aliphatic), 1668 (C=O), 1653 (C=N); ^1^H-NMR: 4.82 (s, 2H, CH_2_–C=O), 5.81 (s, 1H, NH), 6.85 (m, 11H, Ar-H), 6.86 (s, 1H, CH of pyrazole ring). Anal. calcd for C_22_H_15_Cl_2_N_3_OS_2_ (472.40): C, 55.94; H, 3.20; N, 8.90; found: C, 55.75; H, 3.12; N, 8.79.

#### 4.1.15. Formation of (*E*)-2-(((4-(4-hydroxyphenyl)-6-(thiophen-2-yl)pyrimidin-2-yl)imino)methyl)phenol (13a)

A mixture of 11a (2.69 g; 0.01 mol) and salicylaldehyde (1.22 mL; 0.01 mol) with drops of piperidine was refluxed for 3 h in absolute ethanol (20 mL). The reaction mixture was cooled, and acidified with diluted HCl and the resulting solid was filtered out, dried, and recrystallized to give compound 13a.

Yield 51%; brown crystal; mp 133–135 °C (EtOH); IR (cm^−1^) *ν*: 3417 (OH), 1639 (C=N); ^1^H-NMR: 6.84–8.02 (m, 11H, Ar-H), 7.51 (s, 1H, CH of pyrimidine ring), 7.56 (s, 1H, CH=N), 10.44 (s, 1H, OH), 10.44 (s, 1H, OH). Anal. calcd for C_21_H_15_N_3_O_2_S (373.43): C, 67.54; H, 4.05; N, 11.25; found: C, 67.39; H, 4.00; N, 11.34.

#### 4.1.16. Formation of 3-(4-hydroxyphenyl)-*N*-phenyl-5-(thiophen-2-yl)-4,5-dihydro-1*H*-pyrazole-1-carbothioamide (14a)

A mixture of 7a (2.44 g; 0.01 mol) and phenyl isothiocyanate (1.35 mL; 0.01 mol) was refluxed for 10–12 h in the presence of benzene (20 mL) and drops of triethylamine. The obtained solid was filtered out, dried, and recrystallized to get compound 14a.

Yield 81%; white crystal; mp 245–247 °C, EtOH; IR (cm^−1^) *ν*: 3323 (OH), 3182 (NH), 3028 (CH aliphatic), 1604 (C=N); ^1^H-NMR: 3.34 (d, d, 2H, CH of pyrazole), 3.83 (d, d, 1H, CH of pyrazole ring), 7.14–7.88 (m, 12H, Ar-H), 7.04 (s, 1H, NH), 10 (s, 1H, OH). Anal. calcd for C_20_H_17_N_3_OS_2_ (379.50): C, 63.30; H, 4.52; N, 11.07; found: C, 63.08; H, 4.59; N, 11.17.

#### 4.1.17. Formation of ethyl 2-(3-(4-hydroxyphenyl)-5-(thiophen-2-yl)-4,5-dihydro-1*H*-pyrazol-1-yl)acetate (15a)

A mixture of 7a (2.44 g; 0.01) and ethyl chloroacetate (1.22 mL; 0.01 mol) was refluxed for 8 h in ethanolic sodium hydroxide solution (20 mL). The reaction mixture was cooled, and acidified with diluted hydrochloric acid and the resulting solid was filtered out, dried, and recrystallized to give compound 15a.

Yield 75%; brown crystal; mp 65 °C, EtOH; IR (cm^−1^) *ν*: 3254 (OH), 1731 (C=O), 1604 (C=N); ^1^H-NMR: 1.02 (t, 3H, CH_3_), 2.91 (s, 2H, CH_2_), 3.59 (d, d, 2H, CH_2_ of pyrazole ring), 3.93 (d, d, 1H, CH of pyrazole ring), 4.09 (q, 2H, CH_2_), 6.77–7.99 (m, 7H, Ar-H), 10.44 (s, 1H, OH). Anal. calcd for C_17_H_18_N_2_O_3_S (330.40): C, 61.80; H, 5.49; N, 8.48; found: C, 61.65; H, 5.45; N, 8.57.

#### 4.1.18. Formation of 2-(3-(4-hydroxyphenyl)-5-(thiophen-2-yl)-4,5-dihydro-1*H*-pyrazol-1-yl)-*N*-(4-sulfamoylphenyl)acetamide (16a)

A mixture of 7a (2.44 g; 0.01 mol) and 2-chloro-*N*-(4-sulfamoylphenyl)acetamide (2.48 g; 0.01 mol) was refluxed for 10–12 h in dioxane (20 mL) with drops of triethylamine. The reaction mixture was poured over ice, and the obtained precipitate was filtered out, dried, and recrystallized to get compound 16a.

Yield 66%; brown crystal; mp 158–160 °C (EtOH); IR (cm^−1^) *ν*: 3400 (OH), 3335, 3255 (NH_2_), 3106 (NH), 2800 (CH aliphatic), 1694 (C=O), 1595 (C=N); ^1^H-NMR: 3.06 (d, d, 2H, CH_2_ of pyrazole), 3.90 (d, d, 1H, CH of pyrazole), 5.15 (s, 2H, CH_2_), 6.72 (s, 2H, NH_2_), 6.79–7.844 (m, 11H, H-Ar), 9.70 (s, 1H, NH), 10.80 (s, 1H, OH). Anal. calcd for C_21_H_20_N_4_O_4_S_2_ (456.54): C, 55.25; H, 4.42; N, 12.27; found: C, 55.09; H, 4.33; N, 12.38.

#### 4.1.19. General procedure for the synthesis of compounds (17a) (18a) (19a), and (20a)

A mixture of 7a (2.44 g; 0.01 mol) with anhydrous potassium carbonate (2.76 g; 0.02 mol), tetrabutylammonium bromide (0.96 g; 0.003 mol), and carbon disulfide (10 mL) in ethanol (20 mL) was stirred for 15 min, then different alkyl halide namely ethylchloroacetate and chloroacetic acid and/or different acid chloride namely benzoyl chloride and chloroacetylchloride (0.01 mol) was added with 3 h of continuous stirring. The resulting solid was filtered out, dried, and recrystallized to give compounds 17a, 19a, 18a, and 20a, respectively.

##### 4.1.19.1. Ethyl 2-((3-(4-hydroxyphenyl)-5-(thiophen-2-yl)-4,5-dihydro-1*H*-pyrazole-1-carbonothioyl)thio)acetate (17a)

Yield 87%; yellow crystal; mp 200 °C (EtOH); IR (cm^−1^) *ν*: 3258 (OH), 2974 (CH aliphatic), 1696 (C=O), 1607 (C=N); ^1^H-NMR: 1.16 (t, 3H, CH_3_), 3.14 (d, d, 2H, CH_2_ of pyrazole), 3.39 (d, d, 1H, CH of pyrazole), 4.06 (s, 2H, CH_2_), 4.08 (q, 2H, CH_2_), 6.29–7.69 (m, 7H, Ar-H). Anal. calcd for C_18_H_18_N_2_O_3_S_3_ (406.53): C, 53.18; H, 4.46; N, 6.89; found: C, 53.00; H, 4.38; N, 6.77.

##### 4.1.19.2. Benzoic 3-(4-hydroxyphenyl)-5-(thiophen-2-yl)-4,5-dihydro-1*H*-pyrazole-1-carbothioic thioanhydride (18a)

Yield 83%; yellow crystal; mp 240 °C EtOH; IR (cm^−1^) *ν*: 3395 (OH), 1736–1696 (C=O), 1630 (C=N); ^1^H-NMR: 3.14 (d, d, 2H, CH_2_ of pyrazole ring), 3.69 (d, d, 1H, CH of pyrazole ring), 3.92 (s, 1H, OH), 6.73–8.14 (m, 12H, Ar-H); MS (*m*/*z*) (%): 424 (M+, 32), 360 (100). Anal. calcd for C_21_H_16_N_2_O_2_S_3_ (424.55): C, 59.41; H, 3.80; N, 6.60; found: C, 59.24; H, 3.72; N, 6.71.

##### 4.1.19.3. 2-((3-(4-Hydroxyphenyl)-5-(thiophen-2-yl)-4,5-dihydro-1*H*-pyrazole-1-carbonothioyl)thio)acetic acid (19a)

Yield 81%; yellow crystal; mp 223–225 °C (EtOH); IR (cm^−1^) *ν*: 3388 (OH), 2959 (CH aliphatic), 1692 (C=O), 1629 (C=N); ^1^H-NMR: 3.14, 3.18 (d, d, 2H, CH_2_ of pyrazole ring), 3.32 (d, d, 1H, CH of pyrazole ring), 3.60 (s, 2H, CH_2_), 3.89 (s, 1H, OH), 6.84–7.67 (m, 7H, Ar-H), 8.80 (s, 1H, OH). Anal. calcd for C_16_H_14_N_2_O_3_S_3_ (378.48): C, 50.78; H, 3.73; N, 7.40; found: C, 50.39; H, 3.55; N, 7.31.

##### 4.1.19.4. 2-Chloroacetic-3-(4-hydroxyphenyl)-5-(thiophen-2-yl)-4,5-dihydro-1*H*-pyrazole-1-carbothioic thioanhydride (20a)

Yield 83%; yellow crystal; mp 265 °C (EtOH); IR (cm^−1^) *ν*: 3418 (OH), 2961 (CH aliphatic), 1746, 1694 (C=O), 1632 (C=N); ^1^H-NMR: 3.14 (d, d, 2H, CH_2_ of pyrazole ring), 3.60 (d, d, 1H, CH of pyrazole ring), 3.83 (s, 1H, OH), 4.35 (s, 2H, CH_2_ acetyl chloride), 6.88–7.71 (m, 7H, Ar-H). Anal. calcd for C_16_H_13_ClN_2_O_2_S_3_ (396.92): C, 48.42; H, 3.30; N, 7.06; found: C, 48.22; H, 3.22; N, 7.15.

### 4.2. Biological studies

#### 4.2.1. Anti-proliferative activities

The antitumor activities of the newly synthesized derivatives against two cancer cell lines (HepG-2 and MCF-7). Both cell lines were purchased from ATCC (American Type Culture Collection) *via* the holding company for biological products and vaccines (VACSERA, Cairo, Egypt). The cytotoxicity was assessed using the SRB colorimetric assay.^47^

#### 4.2.2. EGFR and VEGFR-2 inhibitory activities

The superior anticancer candidates (2a, 6a, 7a, 10b, 15a, and 18a) were selected to further evaluate their anti-EGFR and anti-VEGFR-2 potentialities based on a homogeneous luminescence release assay, as mentioned before.^34^

#### 4.2.3. Cell cycle analysis

The most active compound 10b was further selected to evaluate the exact phase of cell cycle arrest. Also, the most sensitive cell line (HepG2) was treated with the IC_50_ value of 10b (13.81 μM) to record its impact on the different phases of cell growth (% G0–G1, % S, and % G2/M) using BD FACS Calibur flow cytometer.^48^

#### 4.2.4. Apoptosis analysis

The superior compound 10b was selected to investigate the exact mechanism of cancer cell death whether it be due to apoptosis (programmed cell death) or necrosis (uncontrolled cell death). This was done using the FITC Annexin-V/PI kit.^49^

#### 4.2.5. Antibacterial and antifungal activities

Using the inhibition zone approach and minimum inhibitory concentrations (MIC), all of the newly synthesized 4-thiophenyl-pyrazole, pyridine, and pyrimidine derivatives were tested for *in vitro* antibacterial and antifungal activities.^50^ They were all tested against Gram-positive bacteria such as *S. aureus* and *B. subtilis* as well as Gram-negative bacteria such as *E. coli* and *P. aeuroginosa*. Also, the antifungal activity was investigated against *C. albicans* and *A. flavus* subtypes. Gentamycin was used as the standard antibacterial drug whereas ketoconazole was regarded as the primary antifungal one.

### 4.3. *In silico* studies

#### 4.3.1. Molecular docking studies

Molecular docking studies are considered an influential method for the interpretation of molecular interactions between the synthesized compounds and the main amino acid residues at the specific binding site of the target receptor.^51,52^ The affinities of the newly synthesized ligands toward the target proteins (EGFR and VEGFR-2) were compared according to the docking score values calculated using the MOE 2019.0102.^42^

All the newly synthesized 4-thiophenyl-pyrazole, pyridine, and pyrimidine derivatives were sketched using the ChemDraw and transferred to the MOE window, optimized for partial charges, and energy minimized as previously reported.^53^ Then, the target two proteins (EGFR and VEGFR-2) were extracted from the PDB (IDs: 2RGP^54^ and 2OH4 (ref. 55)), respectively. Each protein was corrected, 3D hydrogenated, and energy minimized as before.^56^ Finally, two different docking processes were carried out to evaluate the binding affinities of the new candidates toward both EGFR and VEGFR-2 receptor pockets, respectively. The co-crystallized inhibitors were inserted as reference standards and the program specifications were adjusted as previously mentioned.^57^

#### 4.3.2. DFT calculations

Density functional theory (DFT) calculations were performed by using the Gaussian 09W program.^45^ DFT calculations were carried out at the B3LYP level which is a combination with Becke's three-parameter (local, non-local, and Hartree–Fock) hybrid exchange functional with Lee–Yang–Parr correlation functional.^58,59^

Full geometry optimization was performed using 6-31+G(d,p) as a basis set to generate the optimized structures and ground state properties of the studied compounds. The basis set 6-31+G(d,p) was augmented by ‘d’ polarization functions on heavy atoms and ‘p’ polarization functions on hydrogen atoms. Also, the diffuse functions for both hydrogen and heavy atoms were used.

To predict the chemical reactivity, some theoretical descriptors related to conceptual DFT have been determined for compound 10b and reference drugs. The lowest unoccupied (vacant) molecular orbital (LUMO) energy (*E*_LUMO_), the highest occupied molecular orbital (HOMO) energy (*E*_HOMO_), the electronegativity (*χ*), the global softness (*S*), and hardness (*η*) descriptors were all determined from the optimized molecules. It should be noted that the descriptors related to frontier molecular orbitals FMO have been calculated in a very simple way in the context of the Koopmans approximation.^60^

## Author contributions

Conceptualization: A. A. A.-K., A. M. E.-N., and E. M. A.; data curation: S. M. A.-M., A. M. E.-N., A. K. A., N. E. A. A.-E.-S., and E. M. A.; visualization: A. A. A.-K. and E. M. A.; methodology: S. M. A.-M., A. A. A.-K., A. M. E.-N., and E. M. A.; validation: A. A. A.-K. and E. M. A.; supervision: A. A. A.-K., A. M. E.-N., and E. M. A.; writing – review & editing: S. M. A.-M., A. A. A.-K., A. M. E.-N., A. K. A., N. E. A. A.-E.-S., and E. M. A. Finally, all authors revised and approved the final submitted manuscript.

## Conflicts of interest

The authors declared no conflict of interest.

## Supplementary Material

RA-013-D3RA00416C-s001
