# Supplementary material for: Novel 4-thiophenyl-pyrazole, pyridine, and pyrimidine derivatives as potential antitumor candidates targeting both EGFR and VEGFR-2; design, synthesis, biological evaluations, and in silico studies
Source: RSC Adv. 2023 Apr 18;13(18):12184–203. doi: 10.1039/d3ra00416c (PMC10112504; doi:10.1039/d3ra00416c)
Supplement: RA-013-D3RA00416C-s001 [file RA-013-D3RA00416C-s001.pdf]

Electronic Supplementary Material (ESI) for RSC Advances.
This journal is © The Royal Society of Chemistry 2023

Supporting Information

Novel 4-Thiophenyl- Pyrazole, Pyridine, and Pyrimidine Derivatives as Potential Antitumor Candidates Targeting Both EGFR and VEGFR-2; Design, Synthesis, Biological Evaluations, and *In Silico* Studies

Samia M. Al-Muntaser[a,ψ], Ahmed A. Al-Karmalawy[b,ψ,*], Abeer M. El-Naggar[a], Ali Khalil Ali[a], Nour E. A. Abd El-Sattar[a], and Eslam M. Abbass[a,*]

[a] Department of Chemistry, Faculty of Science, Ain Shams University, Abbassiya 11566, Cairo, Egypt.

[b] Pharmaceutical Chemistry Department, Faculty of Pharmacy, Ahram Canadian University, 6th of October City, Giza 12566, Egypt.

* Corresponding authors:

Ahmed A. Al-Karmalawy: Email: akarmalawy@acu.edu.eg

ORCID: 0000-0002-8173-6073

Eslam M. Abbass: Email: eslammorad@sci.asu.edu.eg

ORCID: 0000-0002-3245-1653

ψ: Equal contribution.

Table of Contents

Title	Page
**Yields and elemental analysis**	**S4**
**Table S1.** elemental analysis of target compounds (**1b-20a)**	**S4**
**Figure S1.** Elemental analysis of fifteen compounds	**S5**
**Figure S2.** Elemental analysis of eight compounds	**S6**
**IR spectral data of the target compounds (1b-20a)**	**S7**
**Figure S3. IR** spectrum of compound **1b**	**S7**
**Figure S4. IR** spectrum of compound **2a**	**S7**
**Figure S5. IR** spectrum of compound **2b**	**S8**
**Figure S6. IR** spectrum of compound **3a**	**S8**
**Figure S7. IR** spectrum of compound **4a**	**S9**
**Figure S8. IR** spectrum of compound **5a**	**S9**
**Figure S9. IR** spectrum of compound **6a**	**S10**
**Figure S10. IR** spectrum of compound **7a**	**S10**
**Figure S11. IR** spectrum of compound **8b**	**S11**
**Figure S12. IR** spectrum of compound **9b**	**S11**
**Figure S13. IR** spectrum of compound **10a**	**S12**
**Figure S14. IR** spectrum of compound **10b**	**S12**
**Figure S15. IR** spectrum of compound **11a**	**S13**
**Figure S16. IR** spectrum of compound **12a**	**S13**
**Figure S17. IR** spectrum of compound **12b**	**S14**
**Figure S18. IR** spectrum of compound **13a**	**S14**
**Figure S19. IR** spectrum of compound **14a**	**S15**
**Figure S20. IR** spectrum of compound **15a**	**S15**
**Figure S21. IR** spectrum of compound **16a**	**S16**
**Figure S22. IR** spectrum of compound **17a**	**S16**
**Figure S23. IR** spectrum of compound **18a**	**S17**
**Figure S24. IR** spectrum of compound **19a**	**S17**
**Figure S25. IR** spectrum of compound **20a**	**S18**
**^{1}H NMR spectral data of the target compounds (1b-20a)**	**S19**
**Figure S26.** ^{1}H NMR (400 MHz, DMSO-d_6) spectrum of compound **1b**	**S19**
**Figure S27.** ^{1}H NMR (100 MHz, DMSO-d_6) spectrum of compound **2a**	**S19**

**Figure S28.** ^{1}H NMR (400 MHz, DMSO-d_6) spectrum of compound **2b**	**S20**
**Figure S29.** ^{1}H NMR (100 MHz, DMSO-d_6) spectrum of compound **3a**	**S20**
**Figure S30.** ^{1}H NMR (400 MHz, DMSO-d_6) spectrum of compound **4a**	**S21**
**Figure S31.** ^{1}H NMR (100 MHz, DMSO-d_6) spectrum of compound **5a**	**S21**
**Figure S32.** ^{1}H NMR (400 MHz, DMSO-d_6) spectrum of compound **6a**	**S22**
**Figure S33.** ^{1}H NMR (100 MHz, DMSO-d_6) spectrum of compound **7a**	**S22**
**Figure S34.** ^{1}H NMR (400 MHz, DMSO-d_6) spectrum of compound **8b**	**S23**
**Figure S35.** ^{1}H NMR (100 MHz, DMSO-d_6) spectrum of compound **9b**	**S23**
**Figure S36.** ^{1}H NMR (400 MHz, DMSO-d_6) spectrum of compound **10a**	**S24**
**Figure S37.** ^{1}H NMR (100 MHz, DMSO-d_6) spectrum of compound **10b**	**S24**
**Figure S38.** ^{1}H NMR (400 MHz, DMSO-d_6) spectrum of compound **11a**	**S25**
**Figure S39.** ^{1}H NMR (100 MHz, DMSO-d_6) spectrum of compound **12a**	**S25**
**Figure S40.** ^{1}H NMR (400 MHz, DMSO-d_6) spectrum of compound **12b**	**S26**
**Figure S41.** ^{1}H NMR (100 MHz, DMSO-d_6) spectrum of compound **13a**	**S26**
**Figure S42.** ^{1}H NMR (400 MHz, DMSO-d_6) spectrum of compound **14a**	**S27**
**Figure S43.** ^{1}H NMR (400 MHz, DMSO-d_6) spectrum of compound **16a**	**S27**
**Figure S44.** ^{1}H NMR (100 MHz, DMSO-d_6) spectrum of compound **17a**	**S28**
**Figure S45.** ^{1}H NMR (400 MHz, DMSO-d_6) spectrum of compound **18a**	**S28**
**Figure S46.** ^{1}H NMR (100 MHz, DMSO-d_6) spectrum of compound **19a**	**S29**
**Figure S47.** ^{1}H NMR (400 MHz, DMSO-d_6) spectrum of compound **20a**	**S29**
**Mass spectral data of the target compounds (1b, 3a, 10b, 11a, and 18a)**	**S31**
**Figure S49**. Mass spectrum of compound **1b**	**S31**
**Figure S50**. Mass spectrum of compound **3a**	**S32**
**Figure S51**. Mass spectrum of compound **10b**	**S33**
**Figure S52**. Mass spectrum of compound **11a**	**S34**
**Figure S53**. Mass spectrum of compound **18a**	**S35**
**Schemes**	
**Scheme S1.** Mechanism for the formation of target compounds **17a, 18a, 19a,** and **20a**.	**S36**

Yields and elemental analysis

Table S1. Yields and elemental analysis of target compounds (**1b-20a**).

Code	Yield (%)	Elemental analysis					
		Calculated			Found		
		C	H	N	C	H	N
**1b**	55	55.14	2.85		54.89	2.74	
**2a**	83	65.51	3.78	14.32	65.36	3.70	14.12
**2b**	63	55.51	2.62	12.14	55.34	3.54	12.21
**3a**	75	65.58	4.21	10.49	65.45	4.14	4.42
**4a**	83	63.33	4.43	4.10	63.10	4.32	4.00
**5a**	67	65.29	3.42	9.52	65.18	3.48	9.60
**6a**	77	63.32	3.36	15.55	63.09	3.40	15.48
**7a**	83	63.91	4.95	11.47	63.72	4.84	11.36
**8b**	75	53.11	3.57	8.26	52.89	3.50	8.35
**9b**	81	61.13	3.78	7.50	60.87	3.66	7.43
**10a**	73	55.79	3.68	13.94	55.58	3.72	14.05
**10b**	65	47.47	2.56	11.86	47.30	2.49	11.76
**11a**	76	62.44	4.12	15.60	62.23	4.19	15.51
**12a**	78	61.81	3.07	16.38	61.69	3.00	16.26
**12b**	63	55.94	3.20	8.90	55.75	3.12	8.79
**13a**	51	67.54	4.05	11.35	67.39	4.00	11.34
**14a**	81	63.30	4.52	11.07	63.08	4.59	11.17
**15a**	75	61.80	5.49	8.48	61.65	5.45	8.57
**16a**	66	55.25	4.42	12.27	55.09	4.33	12.38
**17a**	87	53.18	4.46	6.89	53.00	4.38	6.77
**18a**	83	59.41	3.80	6.60	59.24	3.72	6.71
**19a**	81	50.78	3.73	7.40	50.39	3.55	7.31
**20a**	83	48.42	3.30	7.06	48.22	3.22	7.15

Al-Azhar University

The Regional Center for Mycology and Biotechnology

Requester Data:

Name: Dr. Ayman Mohamed Mohamed

Authority: Faculty of Science, Ain Shams University

Sample Data:

Fifteen samples had been submitted for elemental analysis.

Analysis Report:

Sample Code	C%	H%	N%
SB1	65.36	3.70	14.21
Sa	61.69	3.00	16.26
S2	54.89	2.74	0
S5	55.34	2.54	12.21
SB2	65.18	3.48	9.60
SBA	65.45	4.14	4.42
SB10	63.10	4.32	8.00
SB7	63.09	3.40	5.48
SBN	63.72	4.84	1.36
S15	52.89	3.50	5.35
S18	60.87	3.66	7.43
S7	47.30	2.49	1.76
SB5	55.58	3.72	4.05
Sa	55.75	3.12	5.79
SB3	62.23	4.19	5.51

INVESTIGATOR

DIRECTOR

Al-Azhar University Campus - Nasr City, Cairo, Egypt.
Tel: 0202 22620373 Fax : 0202 22620373
E-mail: [illegible]
Website: [illegible]
Facebook : RCMB AZHAR P.O. box mail : 11751 Nasr City, Cairo, Egypt.

Figure S1. Elemental analysis of fifteen compounds.

Al-Azhar University
The Regional Center for Mycology and Biotechnology

Requester Data:

Name: Dr. Mahmoud Khaled Farouk
Authority: Faculty of Science, Ain Shams University

Sample Data:

Eight samples had been submitted for elemental analysis.

Analysis Report:

Sample Code	C%	H%	N%
B3C	67.39	4.00	11.34
N1	61.65	5.45	8.57
N8	63.08	4.59	11.17
F22	55.09	4.33	12.38
PTC	53.00	4.38	6.77
PTC2	59.24	3.72	6.71
PTC3	50.39	3.55	7.31
PTC4	48.22	3.22	7.15

INVESTIGATOR

DIRECTOR

Al-Azhar University Campus - Nasr City, Cairo, Egypt.
Tel: 0202 22620373 Fax : 0202 22620373
E.mail:[illegible]
Website: [illegible] * [illegible]
Facebook : RCMB AZHAR P.O. box mail : 11751 Nasr City Cairo, Egypt.

Figure S2. Elemental analysis of eight compounds.

IR spectral data of the target compounds (1b-20a)

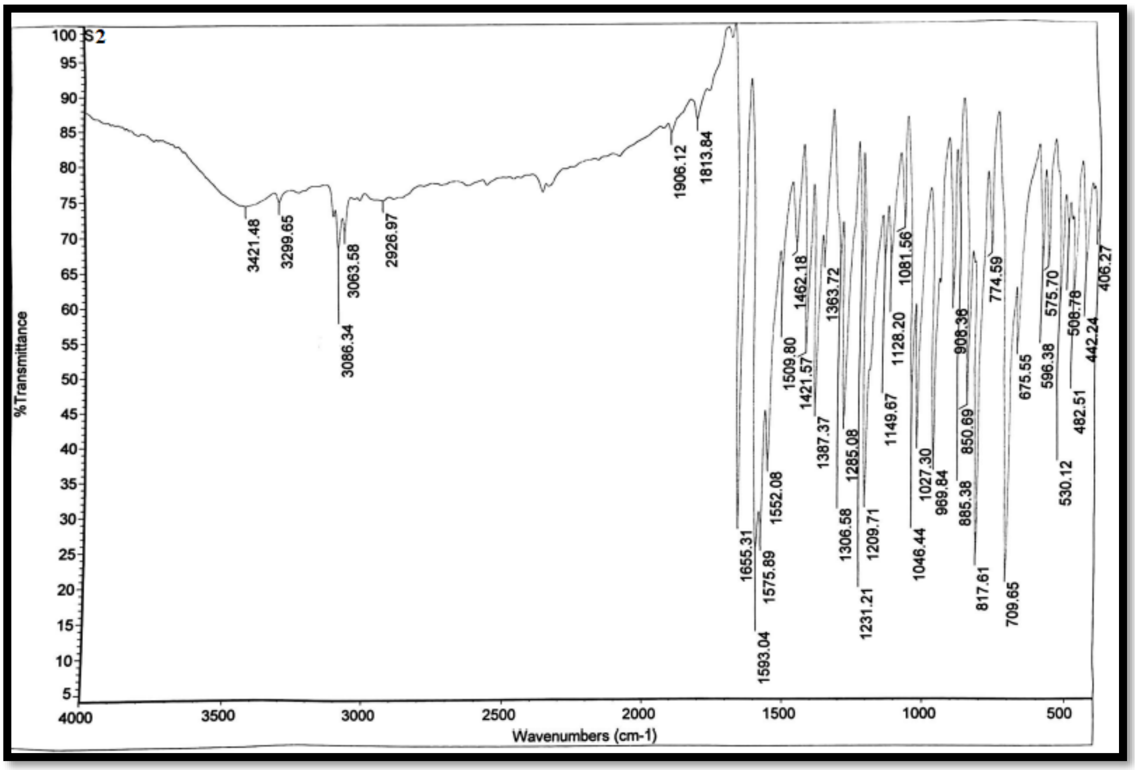

Figure S3. IR spectrum of compound **1b**

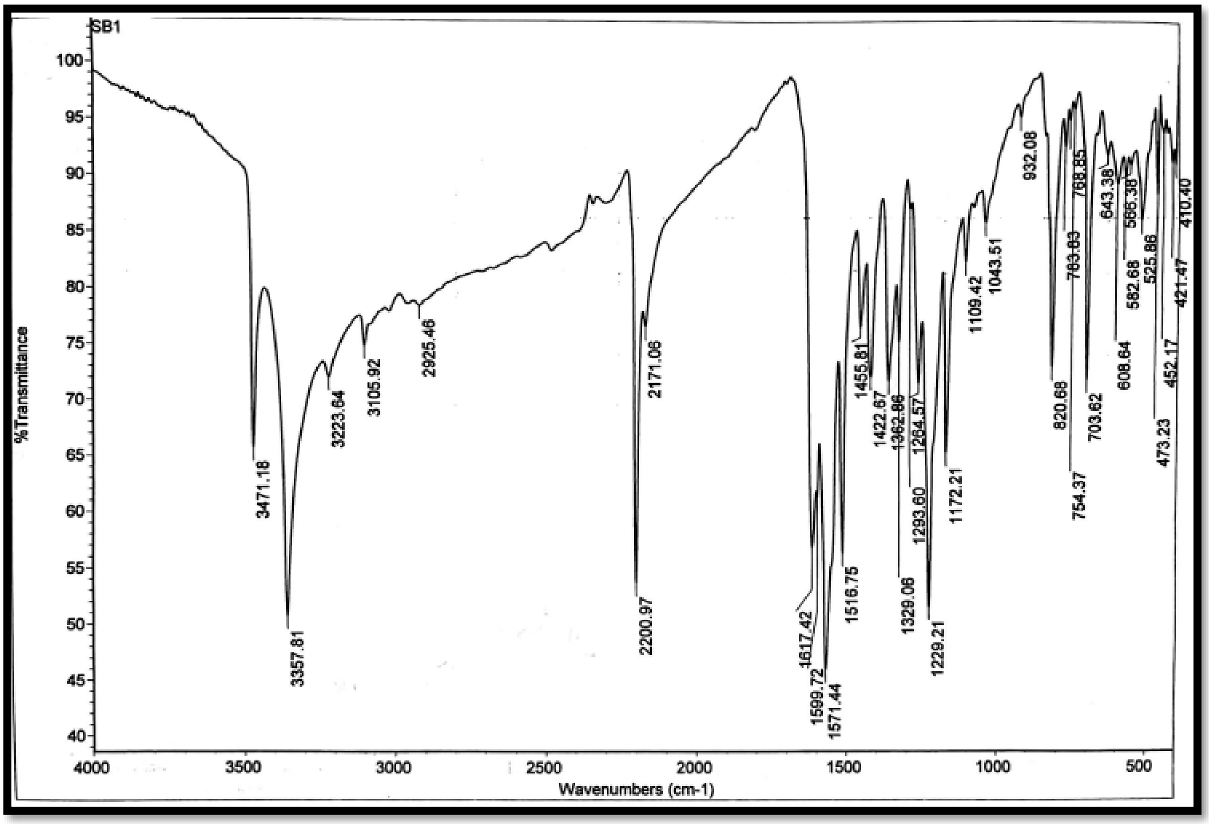

Figure S4. IR spectrum of compound **2a**

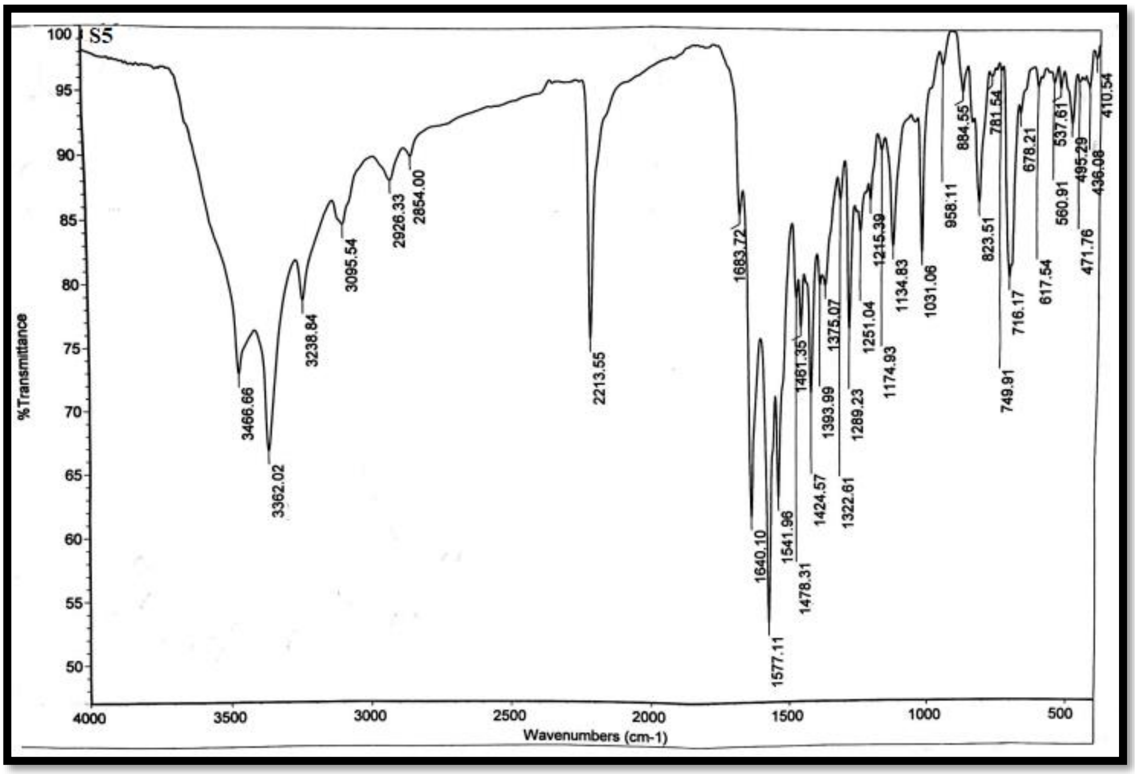

Figure S5. IR spectrum of compound **2b**

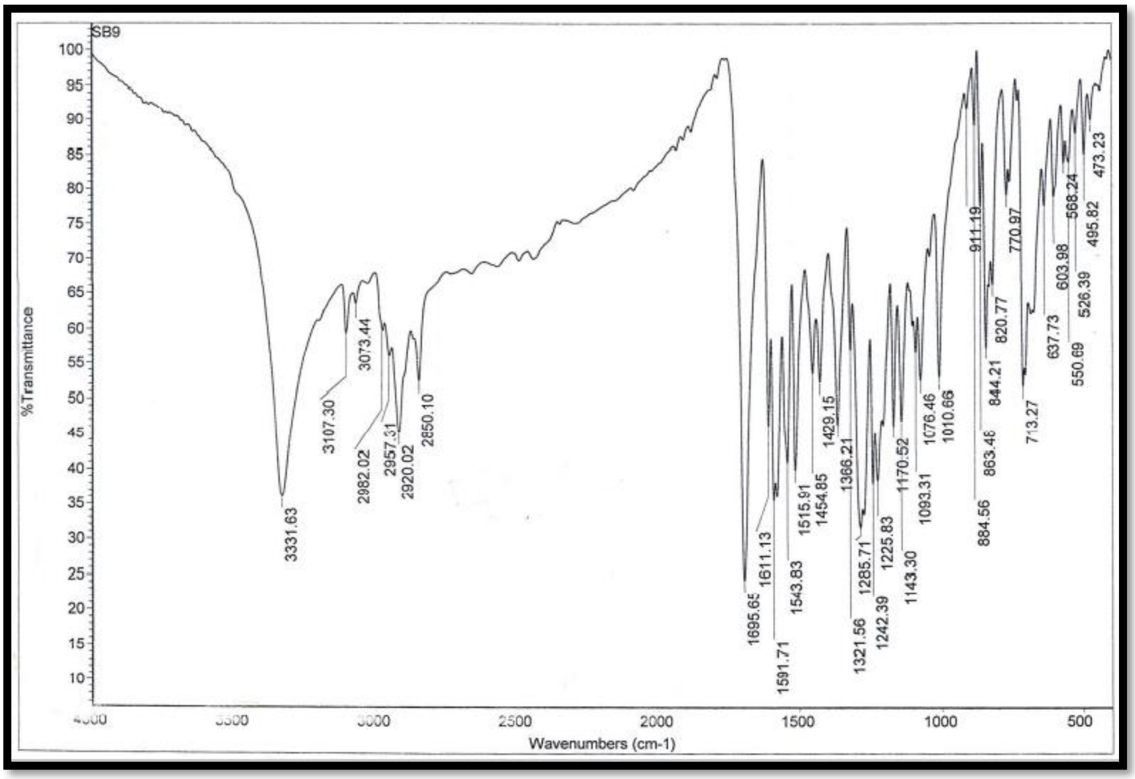

Figure S6. IR spectrum of compound **3a**

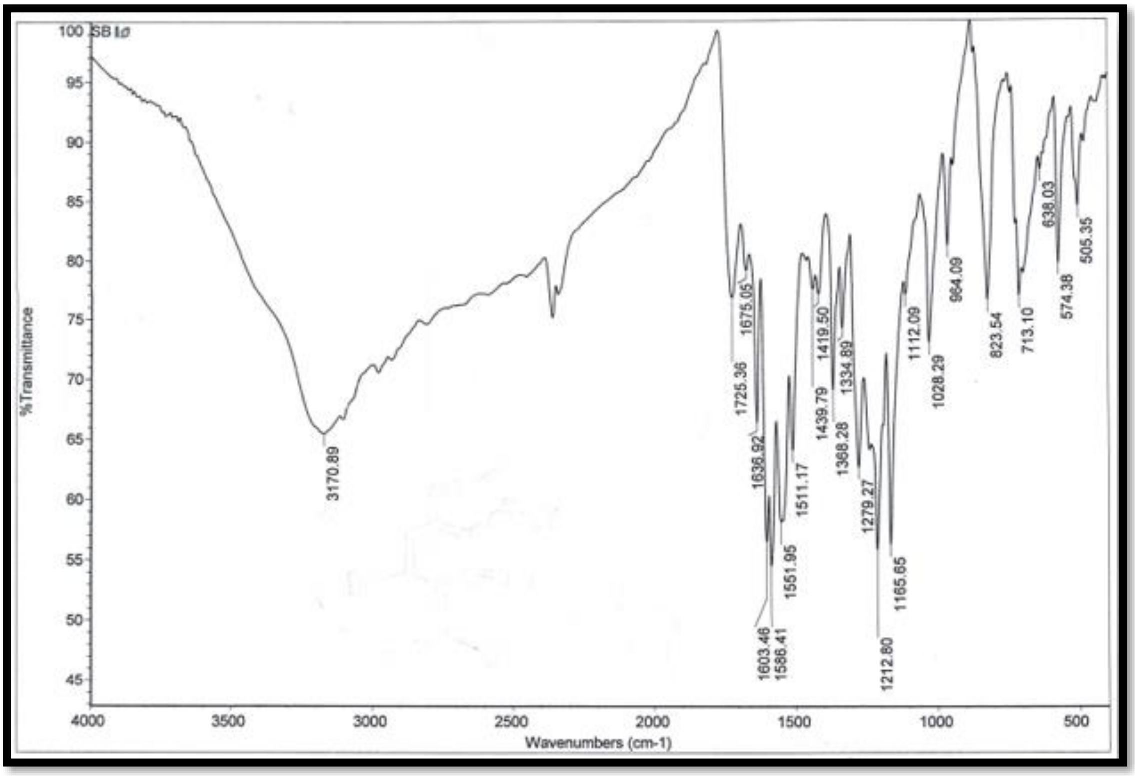

Figure S7. IR spectrum of compound **4a**

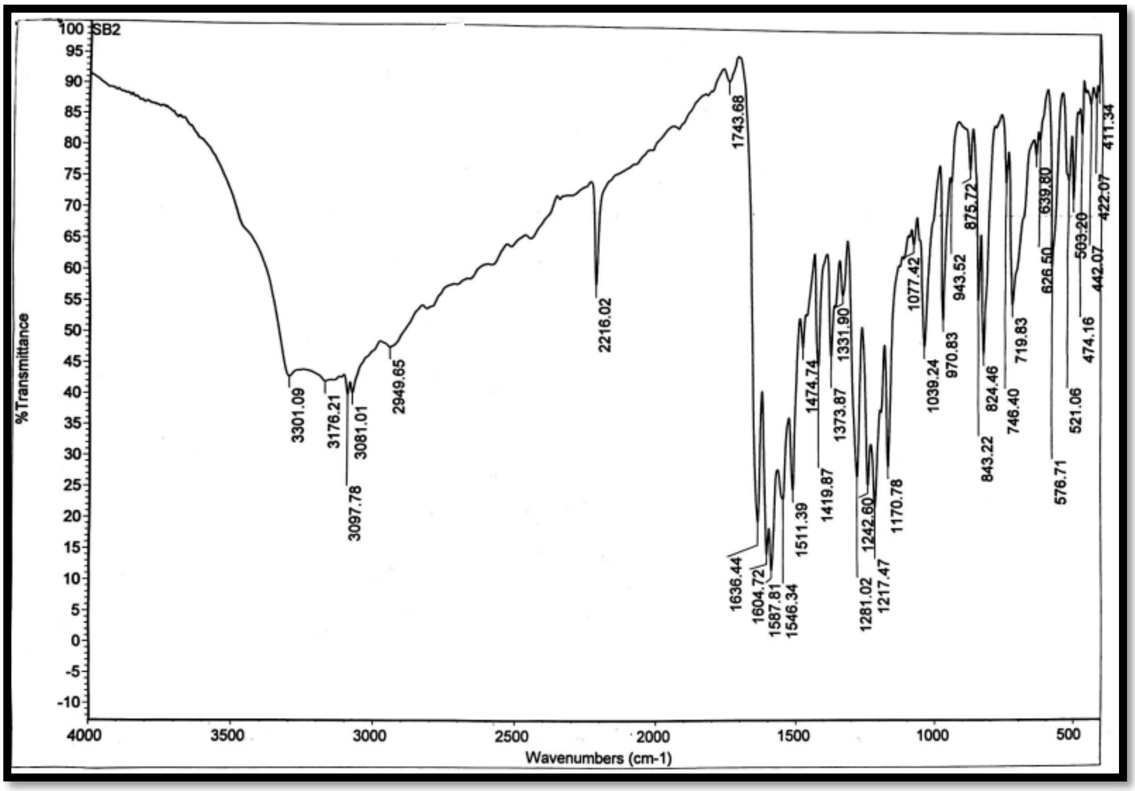

Figure S8. IR spectrum of compound **5a**

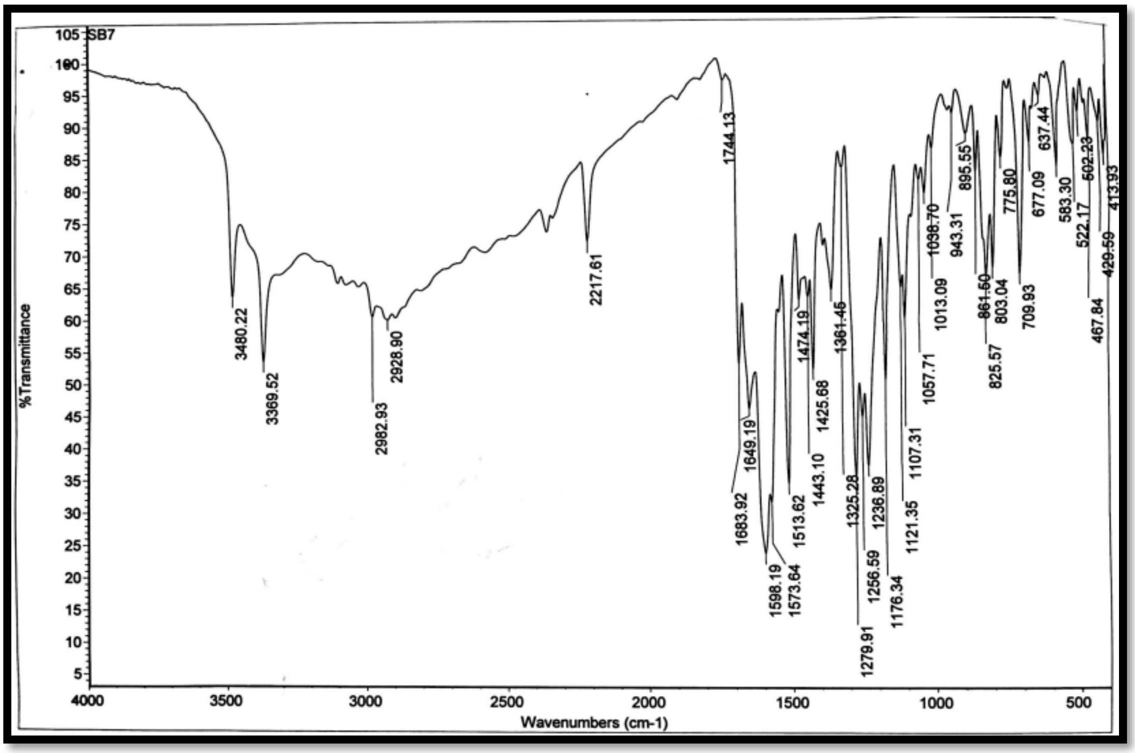

Figure S9. IR spectrum of compound **6a**

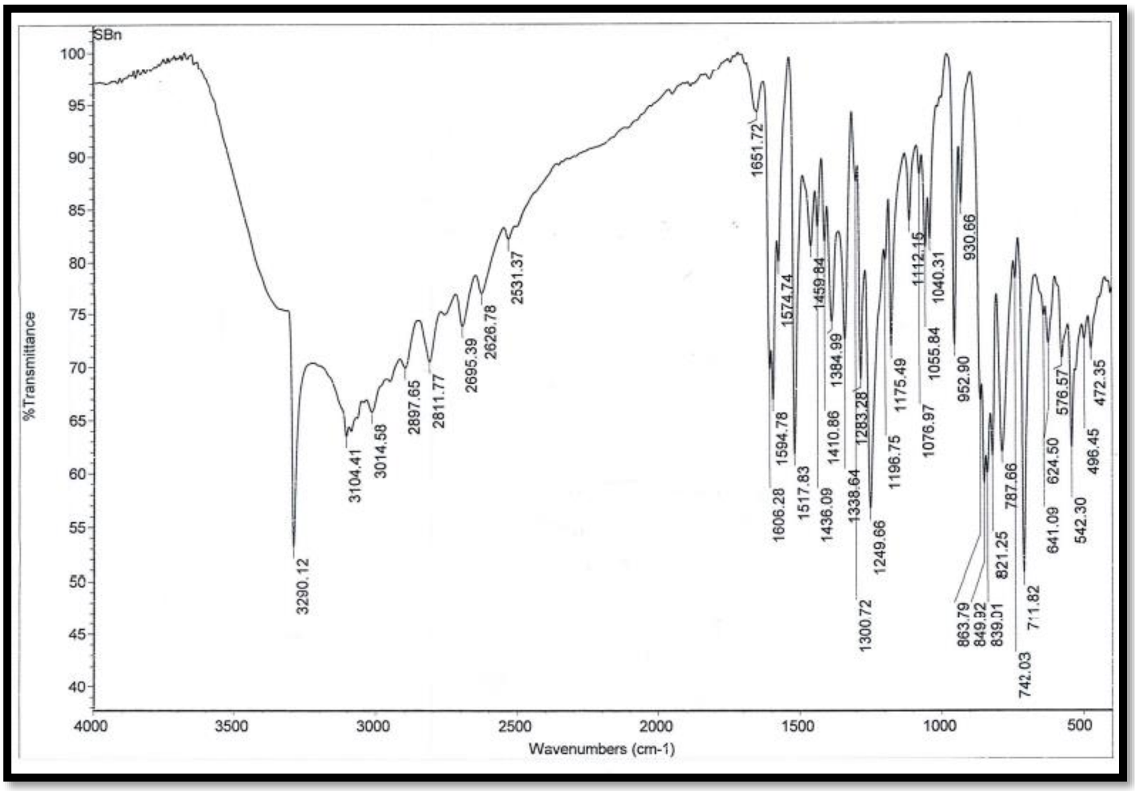

Figure S10. IR spectrum of compound **7a**

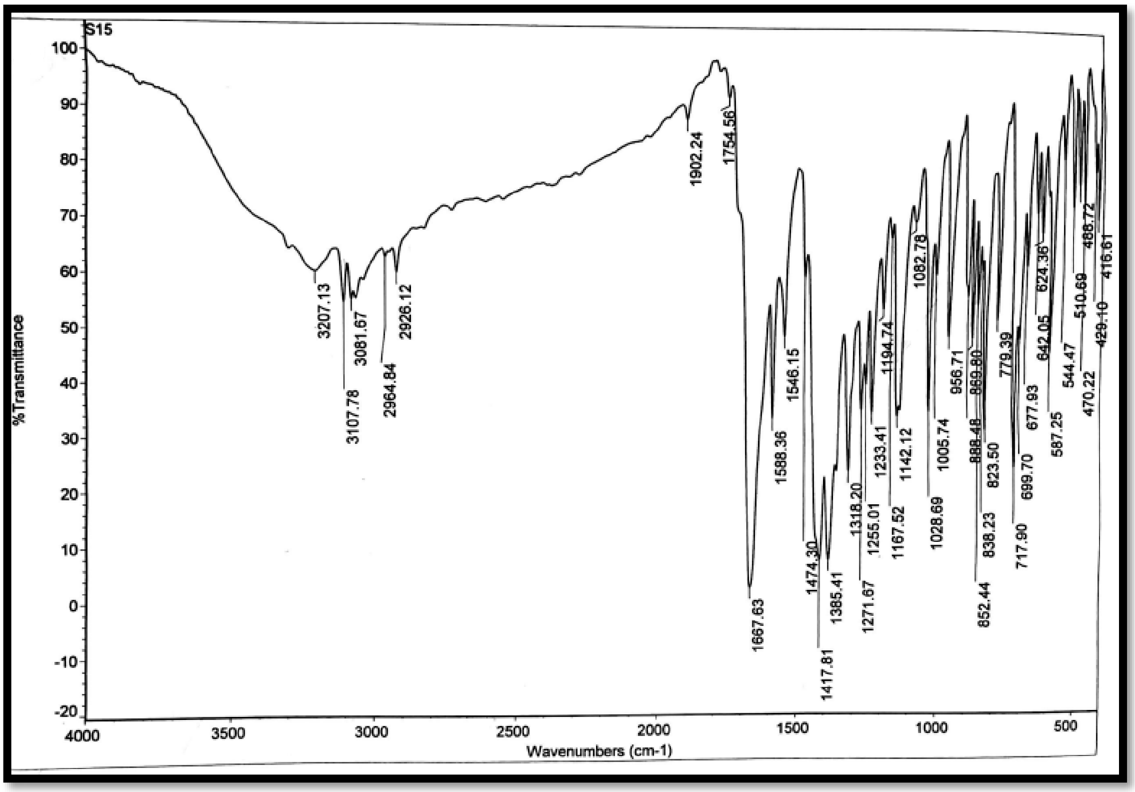

Figure S11. IR spectrum of compound **8b**

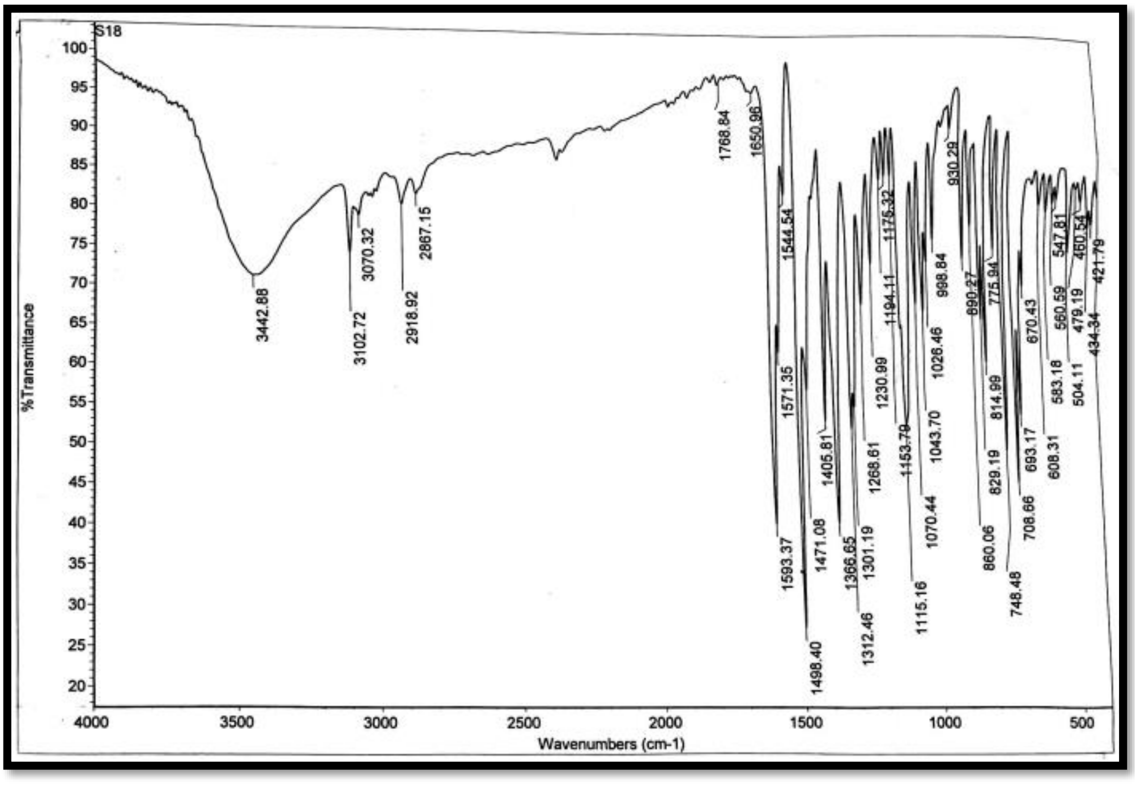

Figure S12. IR spectrum of compound **9b**

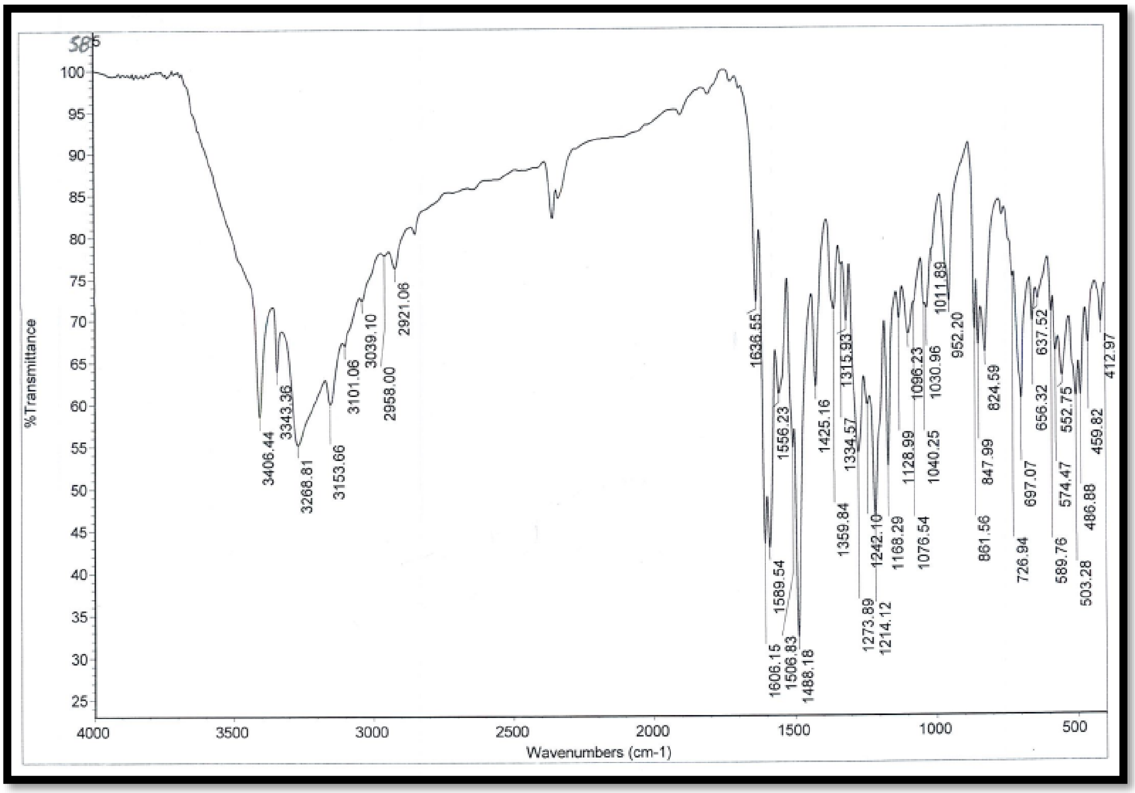

Figure S13. IR spectrum of compound **10a**

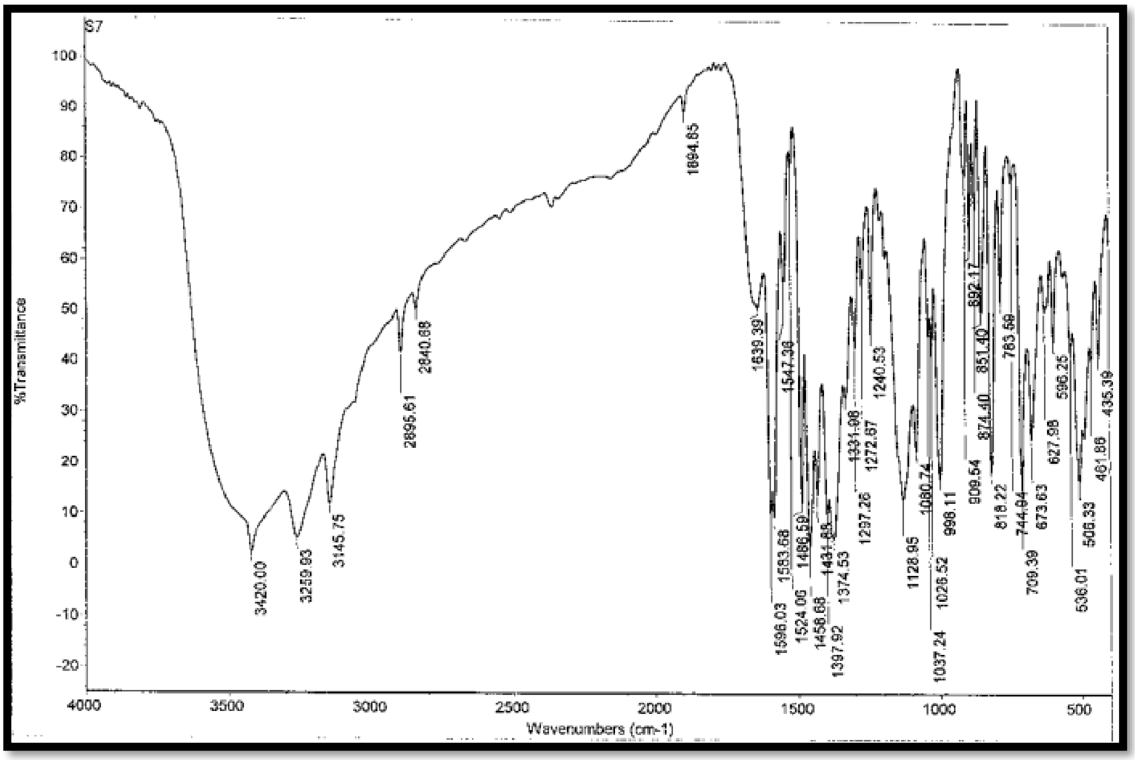

Figure S14. IR spectrum of compound **10b**

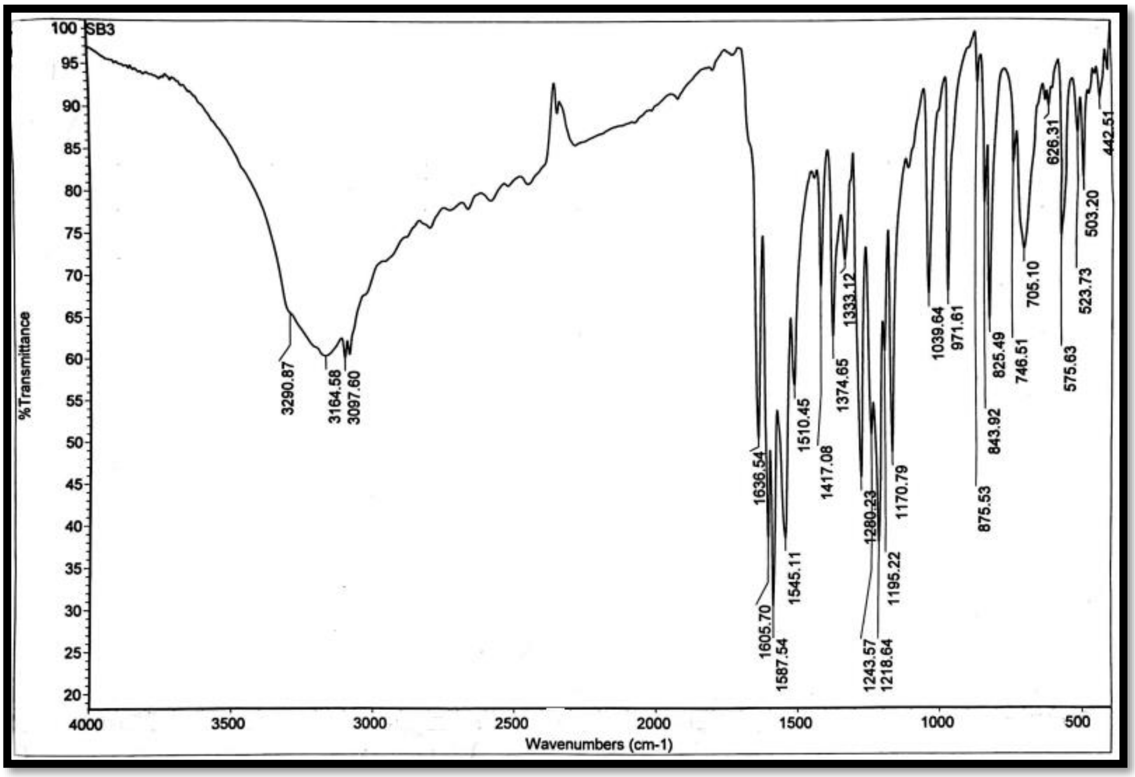

Figure S15. IR spectrum of compound **11a**

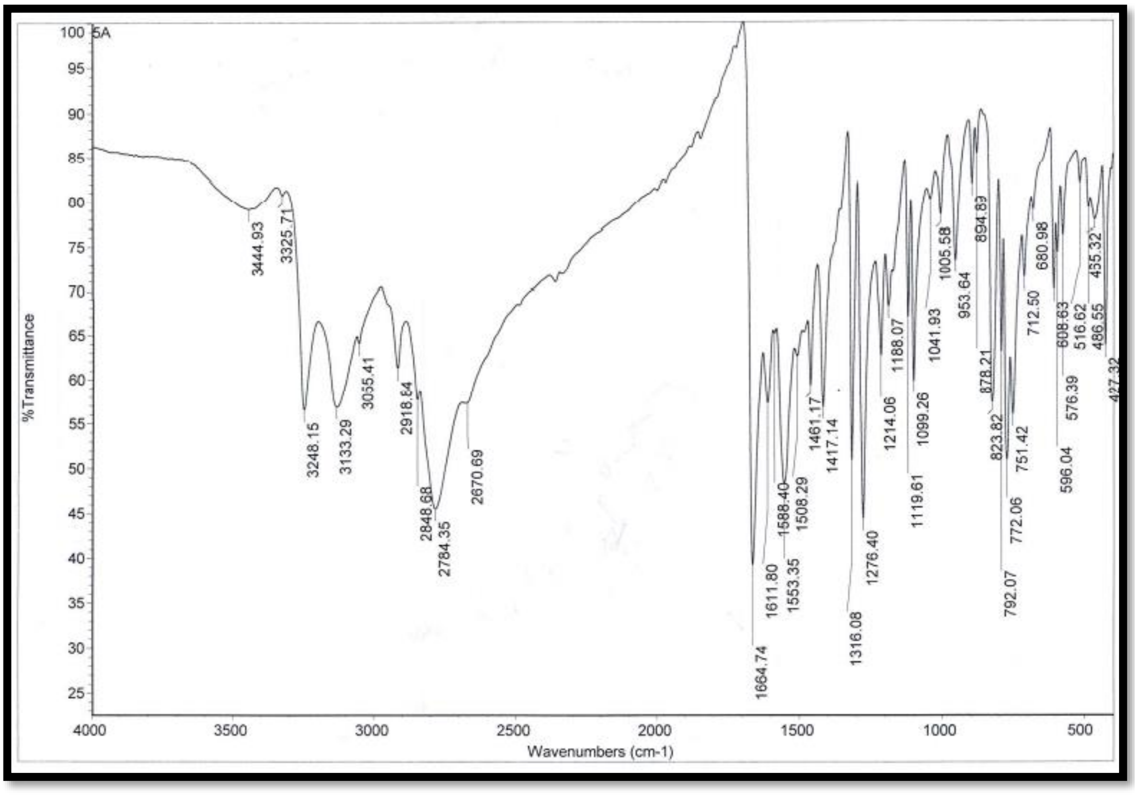

Figure S16. IR spectrum of compound **12a**

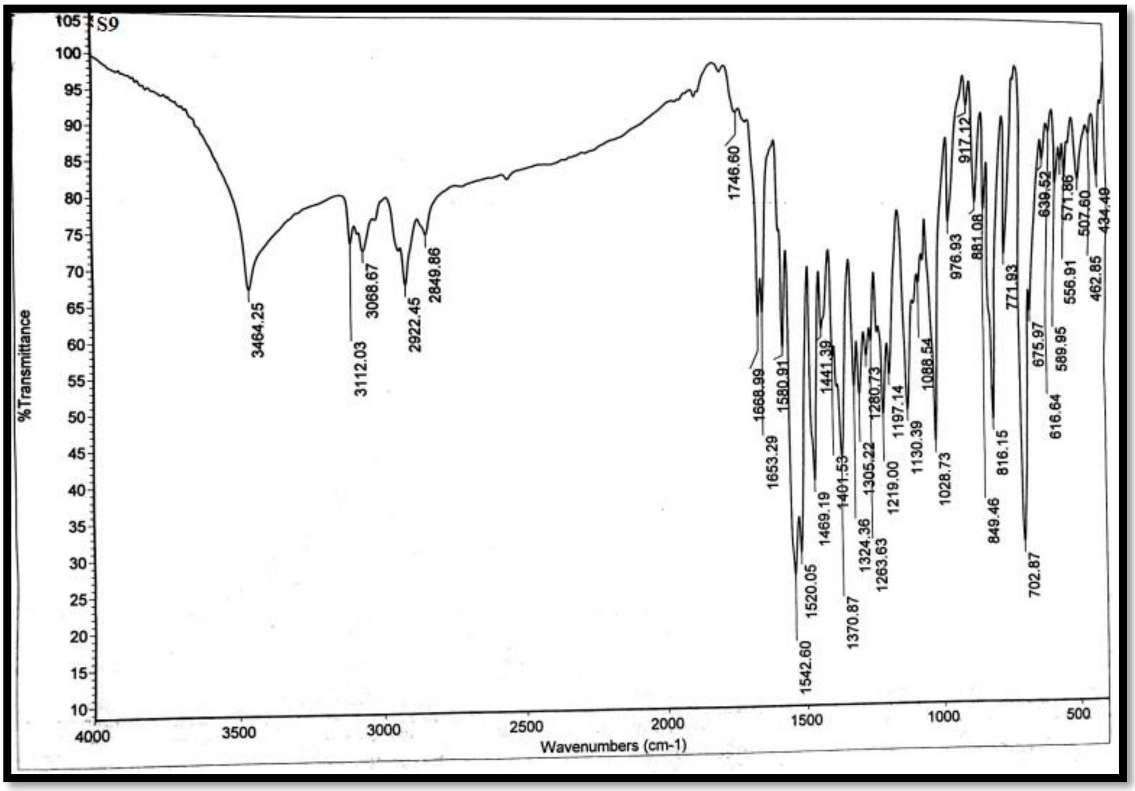

Figure S17. IR spectrum of compound **12b**

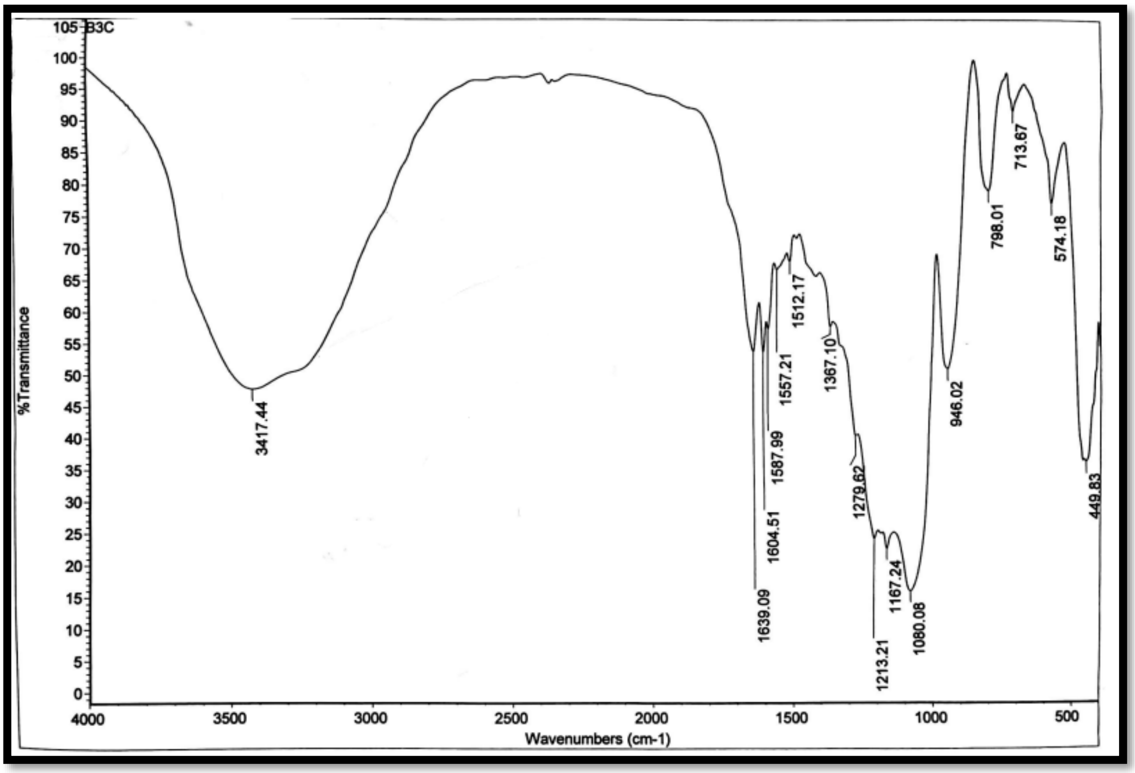

Figure S18. IR spectrum of compound **13a**

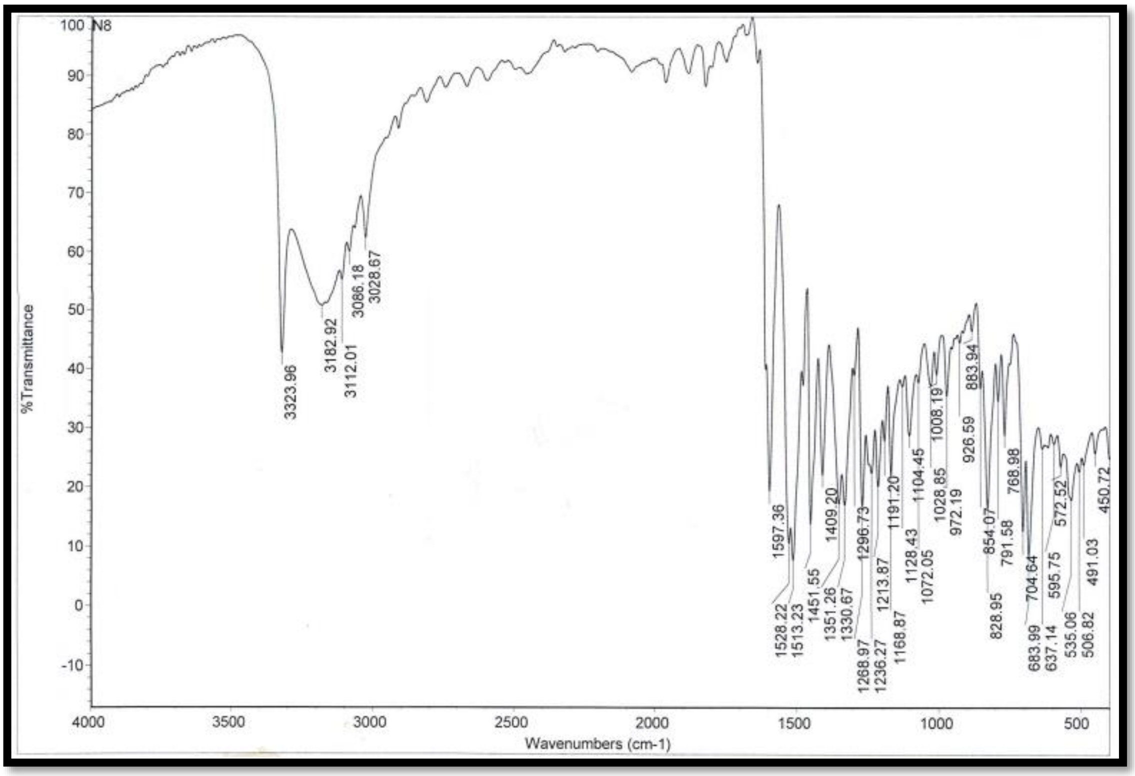

Figure S19. IR spectrum of compound **14a**

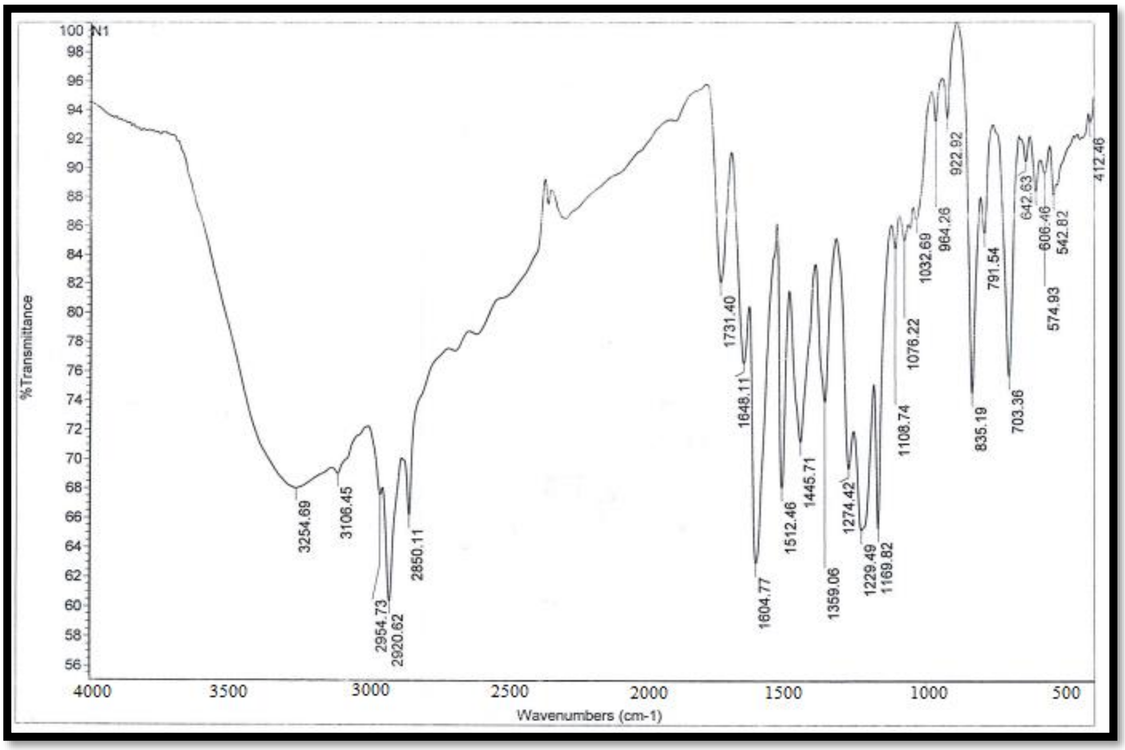

Figure S20. IR spectrum of compound **15a**

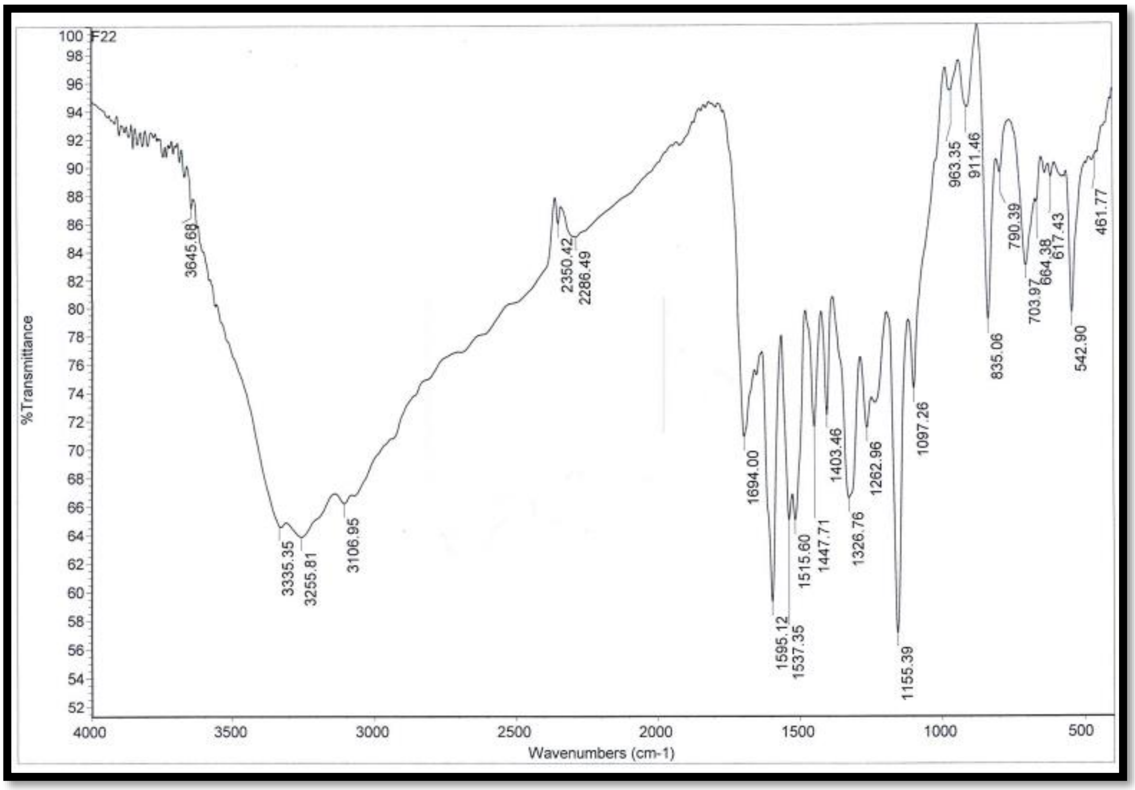

Figure S21. IR spectrum of compound **16a**

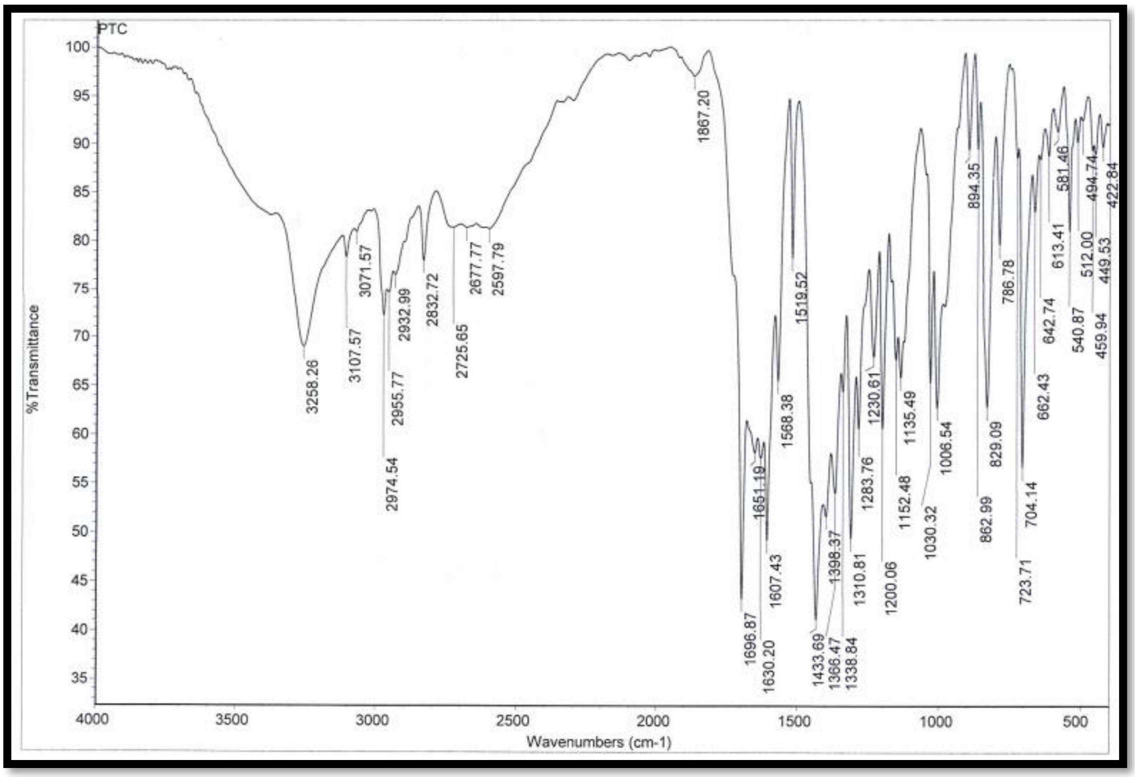

Figure S22. IR spectrum of compound **17a**

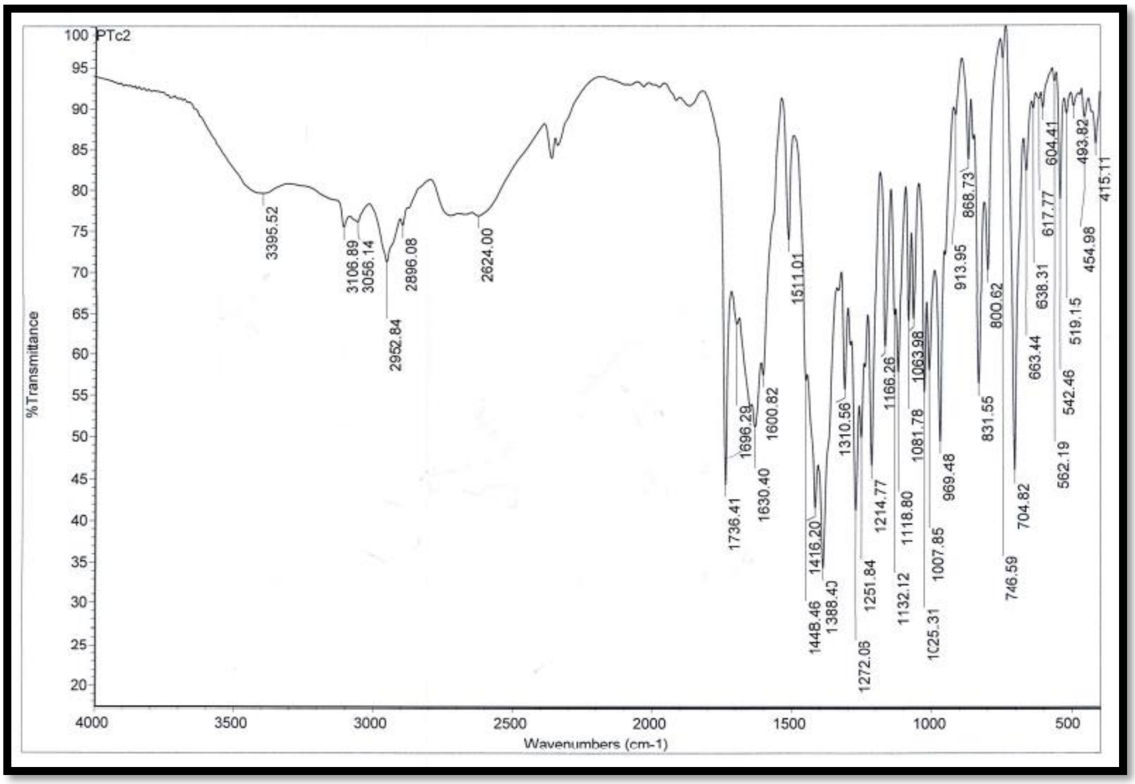

Figure S23. IR spectrum of compound **18a**

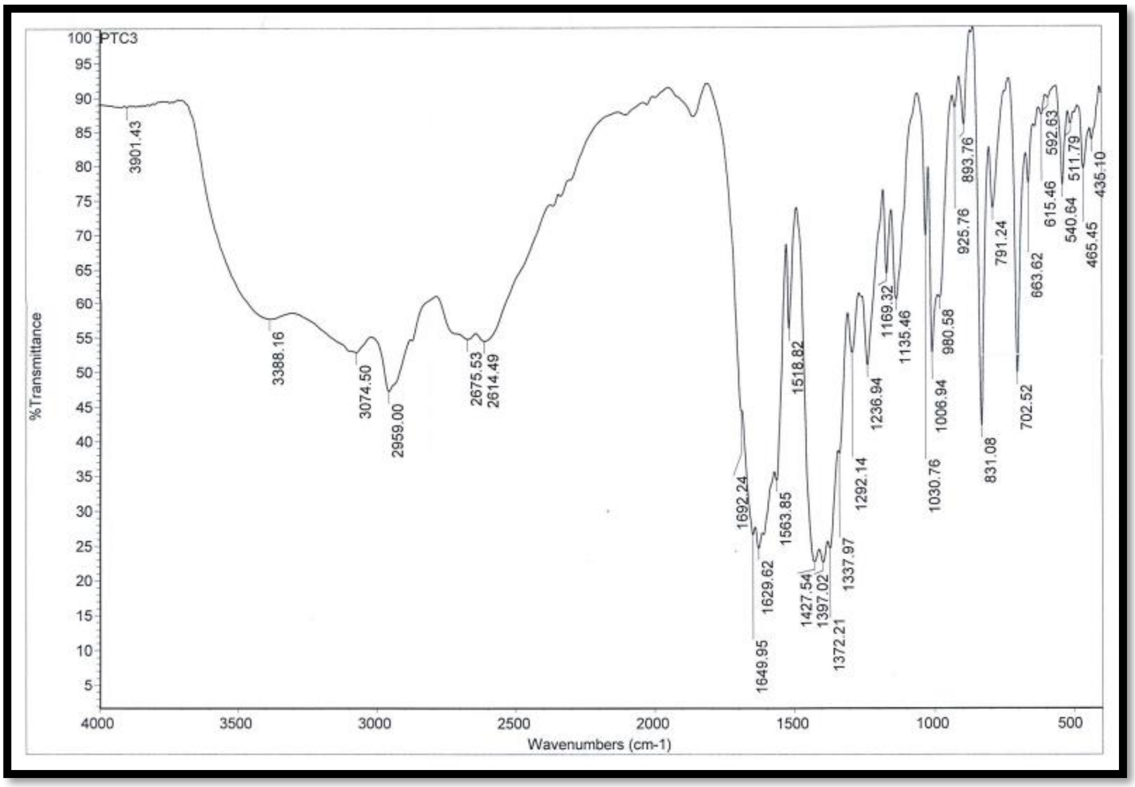

Figure S24. IR spectrum of compound **19a**

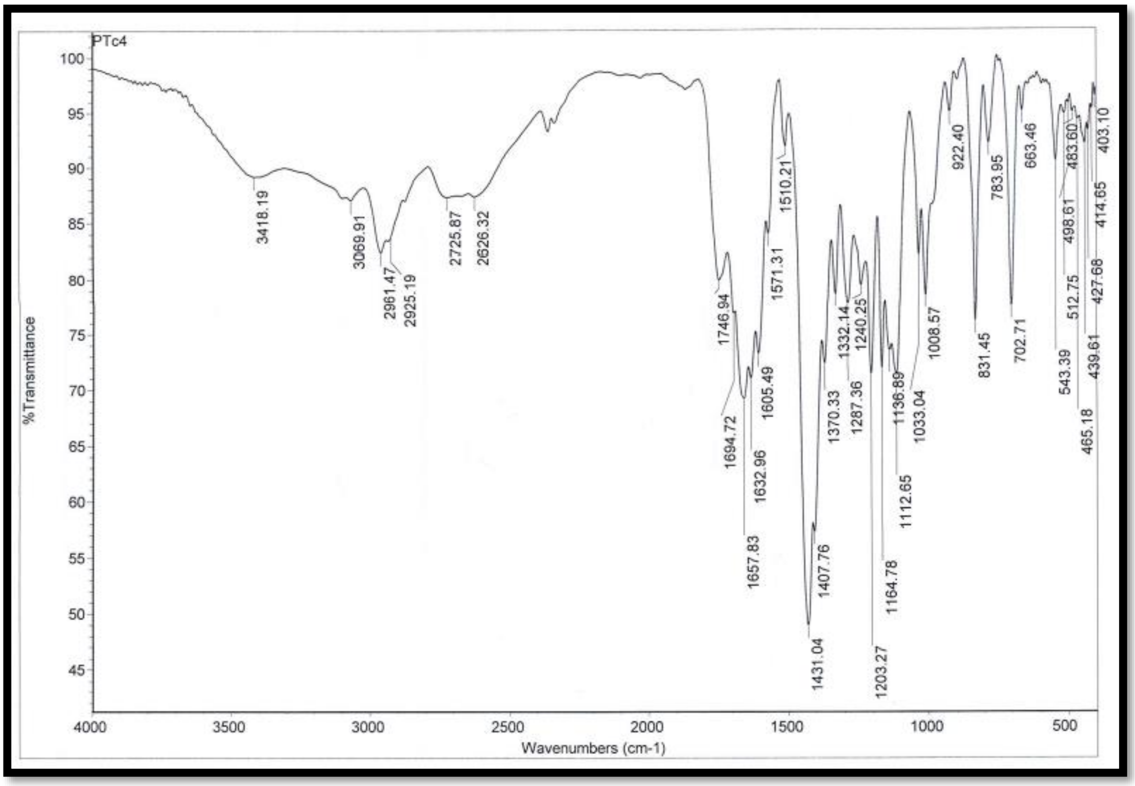

Figure S25. IR spectrum of compound **20a**

^{1}H NMR spectral data of the target compounds (1b-20a)

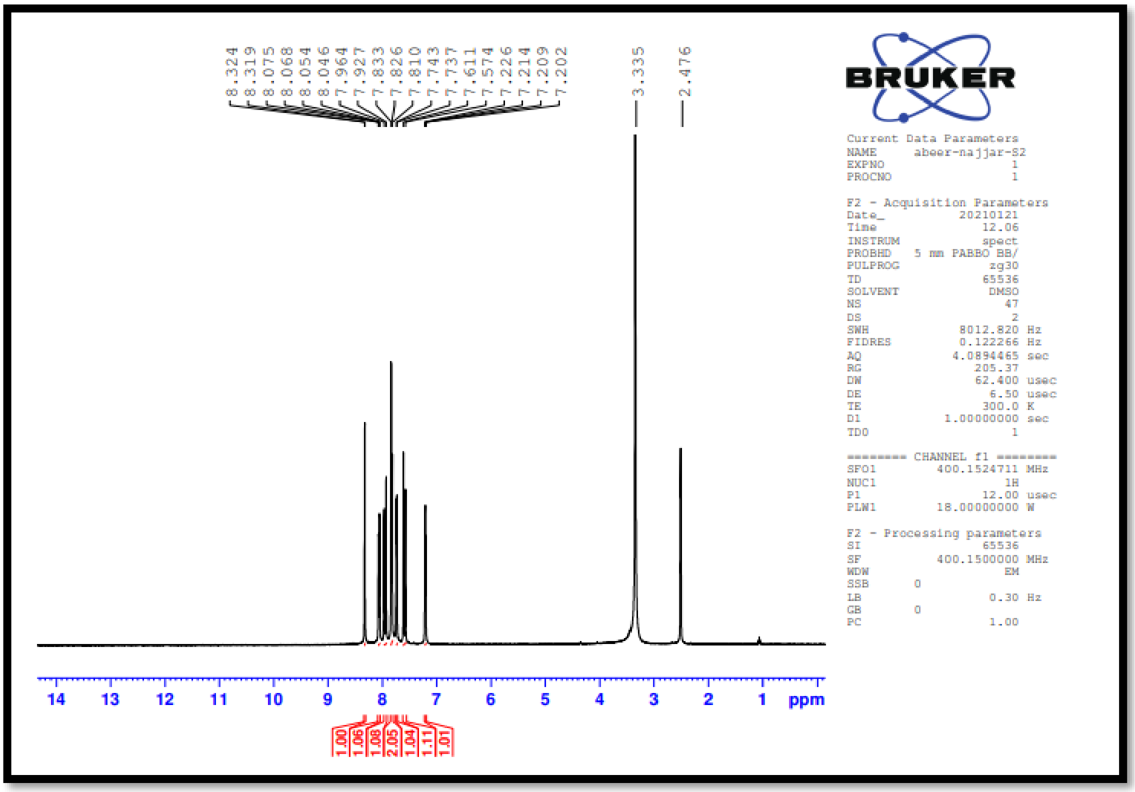

Figure S26. ^{1}H NMR (400 MHz, DMSO-d_6) spectrum of compound **1b**

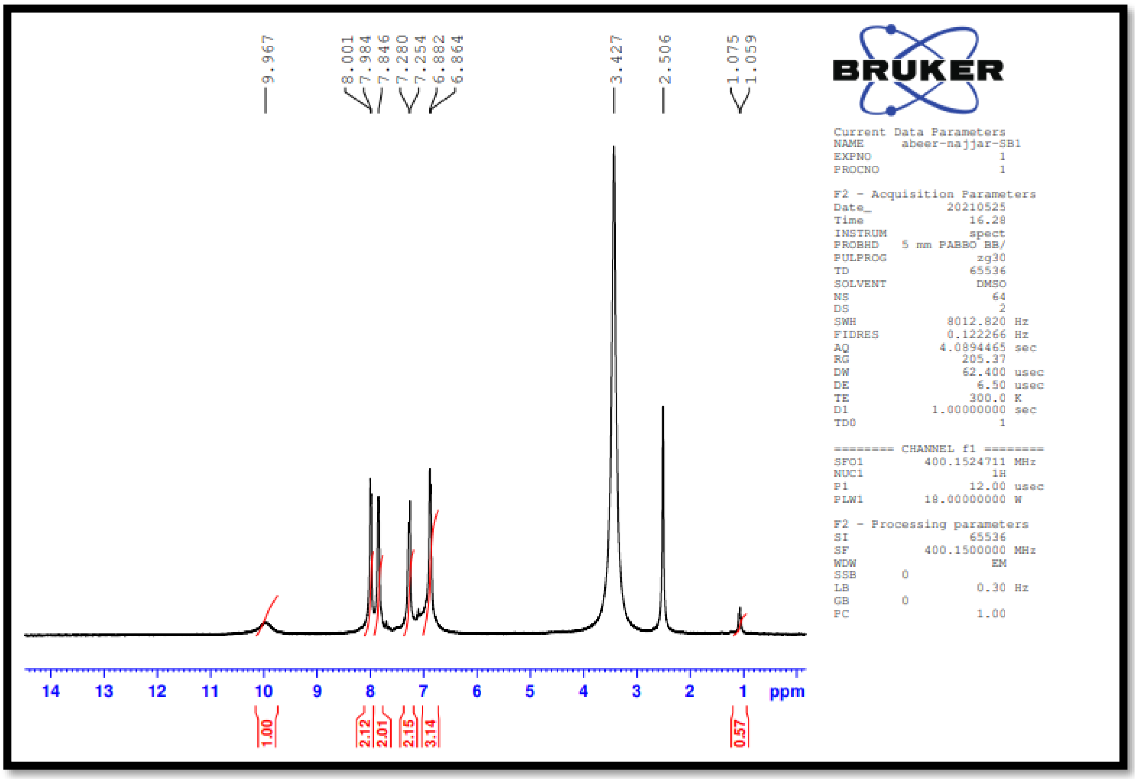

Figure S27. ^{1}H NMR (400 MHz, DMSO-d_6) spectrum of compound **2a**

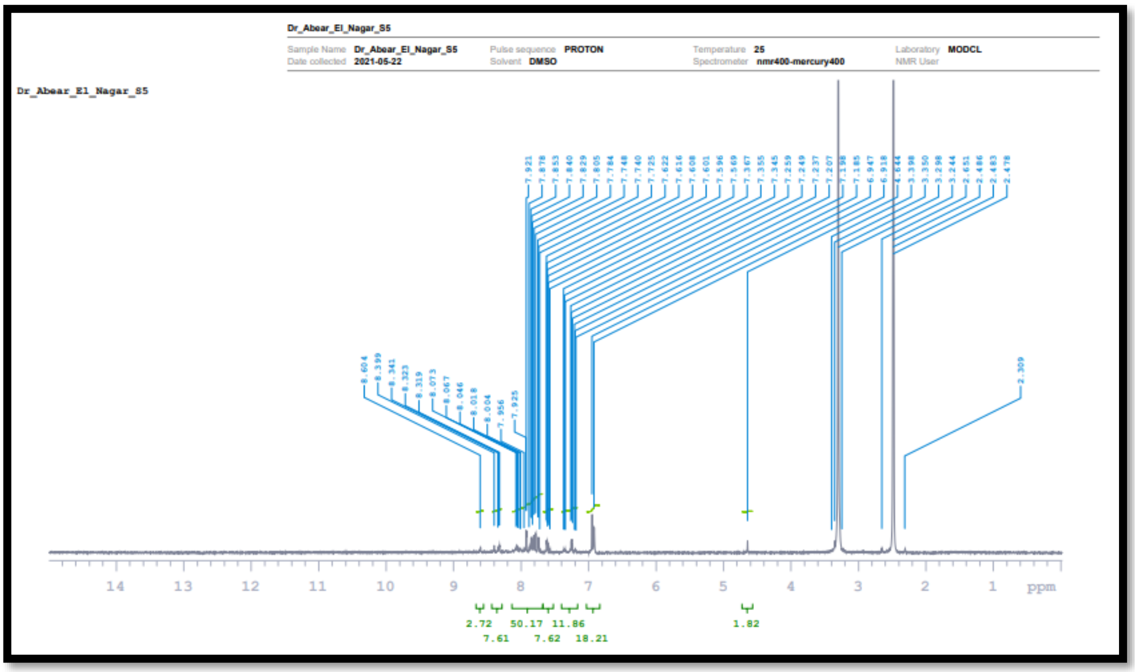

Figure S28. ^{1}H NMR (400 MHz, DMSO-d_6) spectrum of compound **2b**

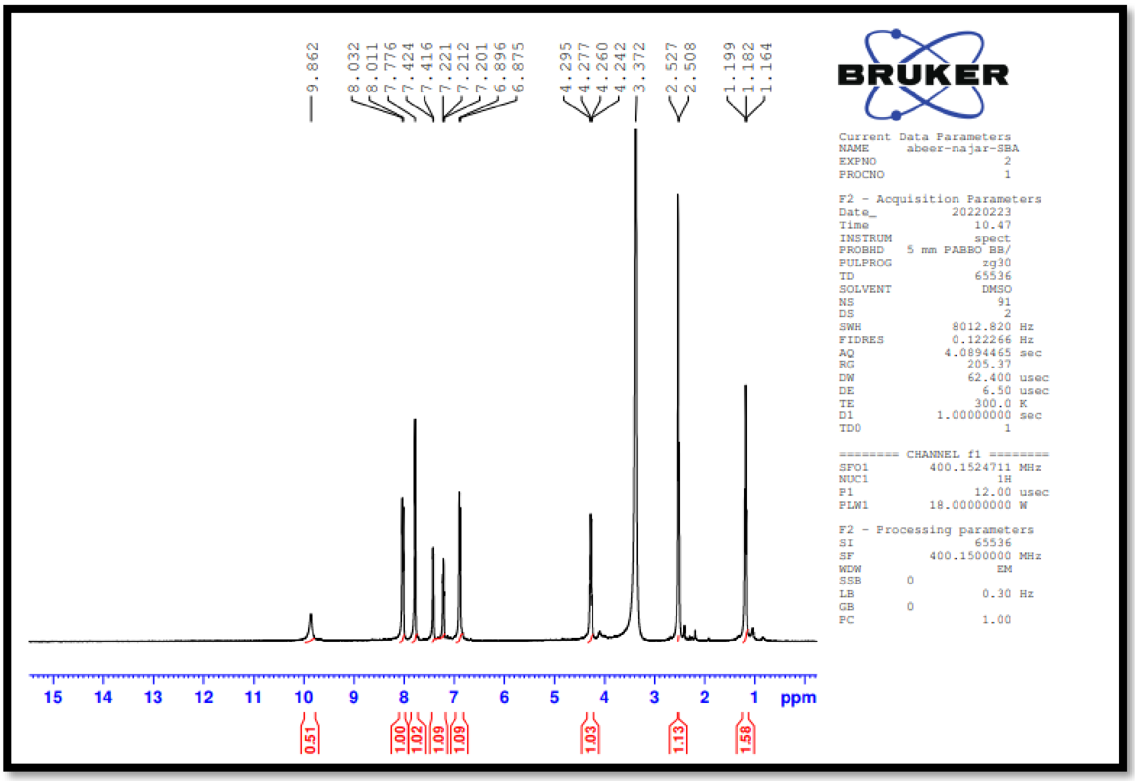

Figure S29. ^{1}H NMR (400 MHz, DMSO-d_6) spectrum of compound **3a**

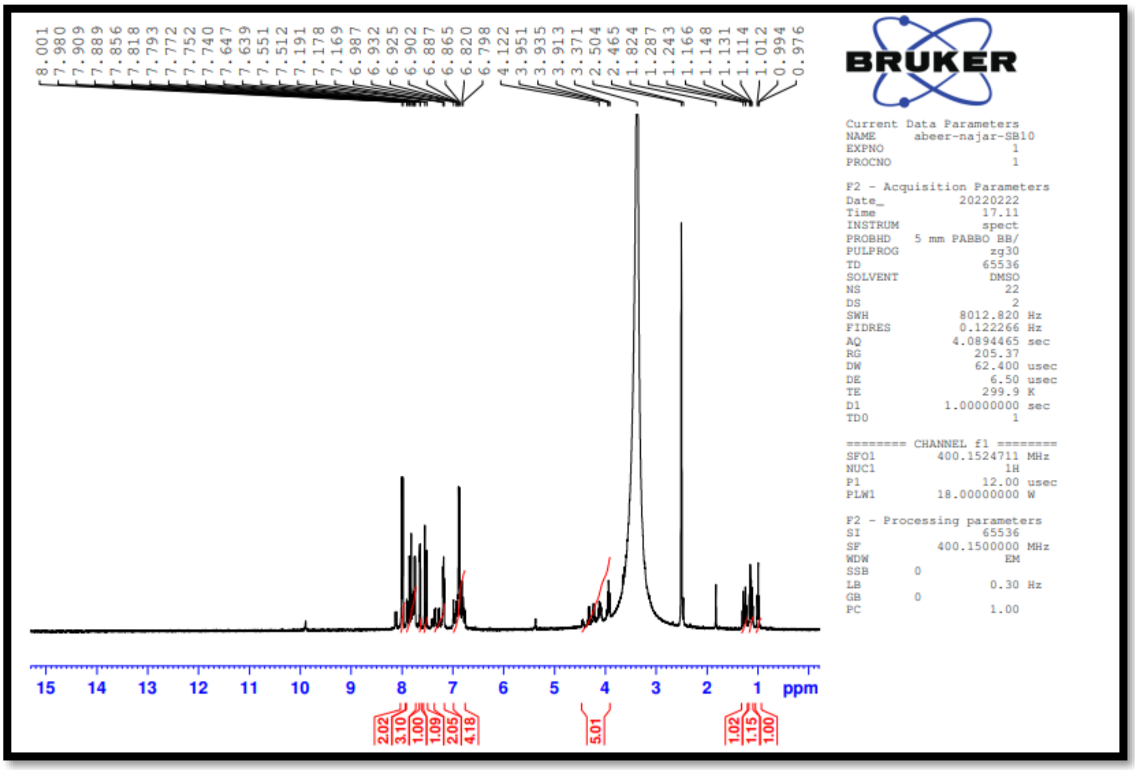

Figure S30. ^{1}H NMR (400 MHz, DMSO-d_6) spectrum of compound **4a**

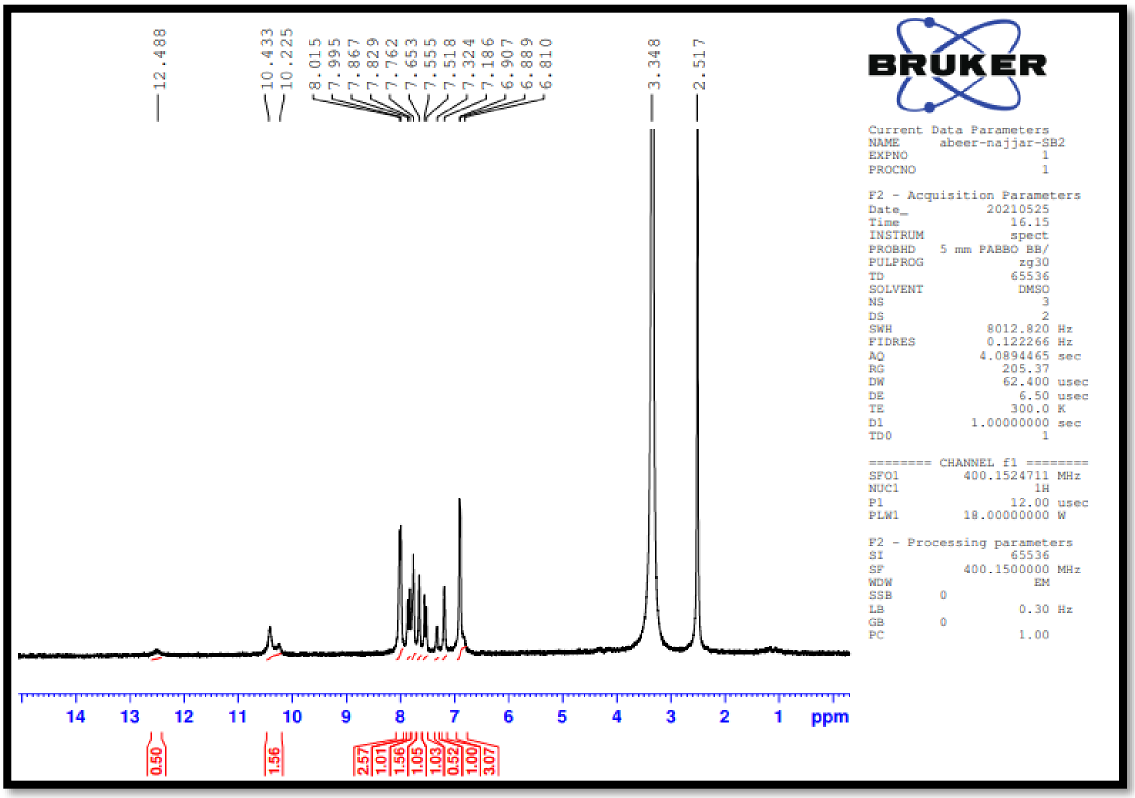

Figure S31. ^{1}H NMR (400 MHz, DMSO-d_6) spectrum of compound **5a**

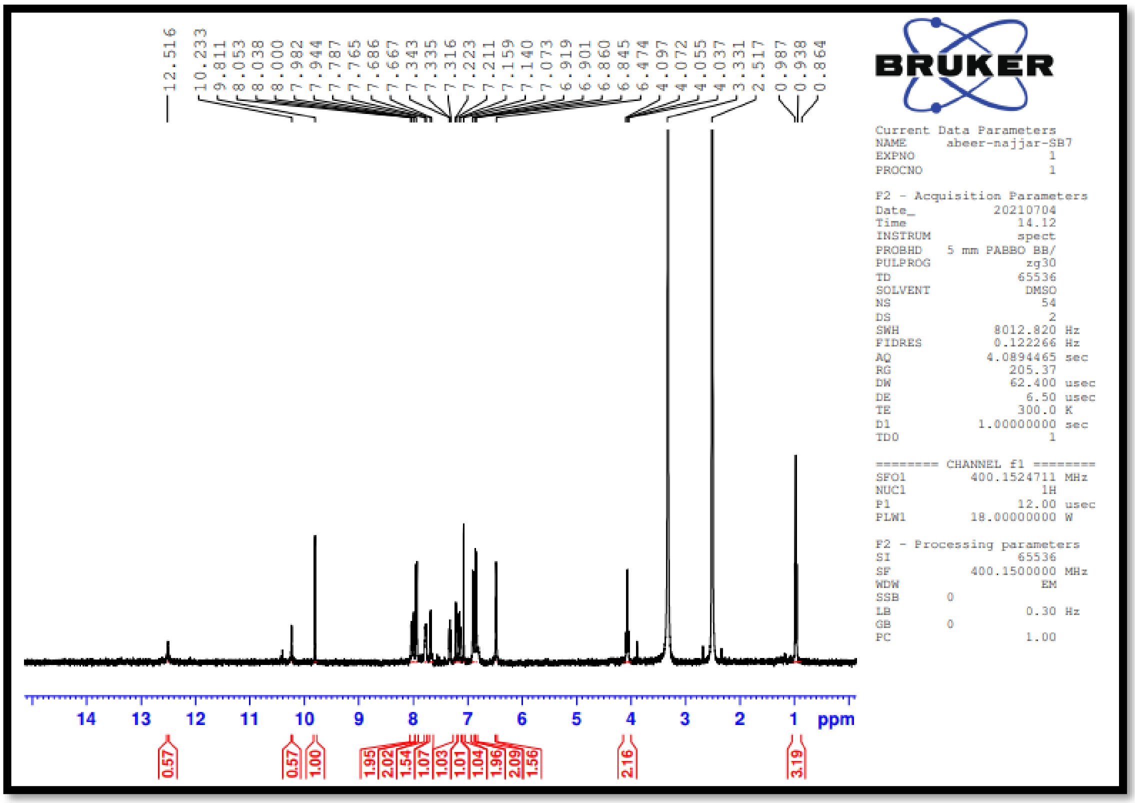

Figure S32. ^{1}H NMR (400 MHz, DMSO-d_6) spectrum of compound **6a**

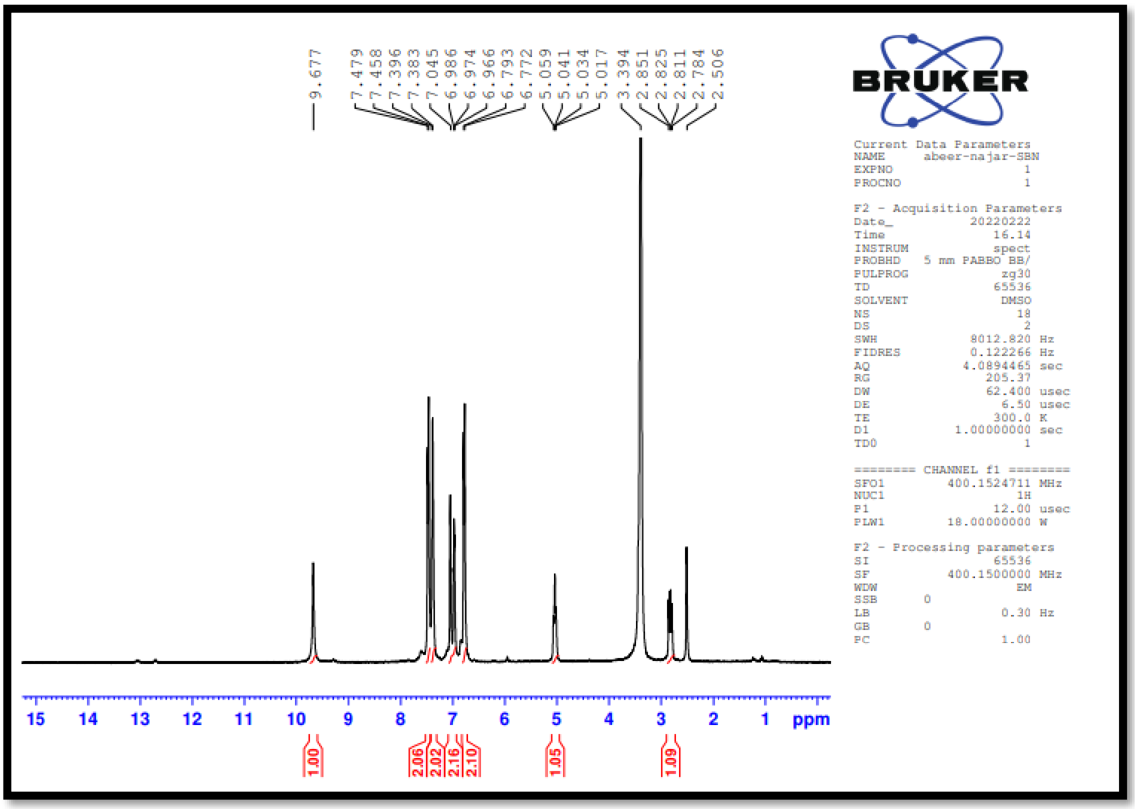

Figure S33. ^{1}H NMR (400 MHz, DMSO-d_6) spectrum of compound **7a**

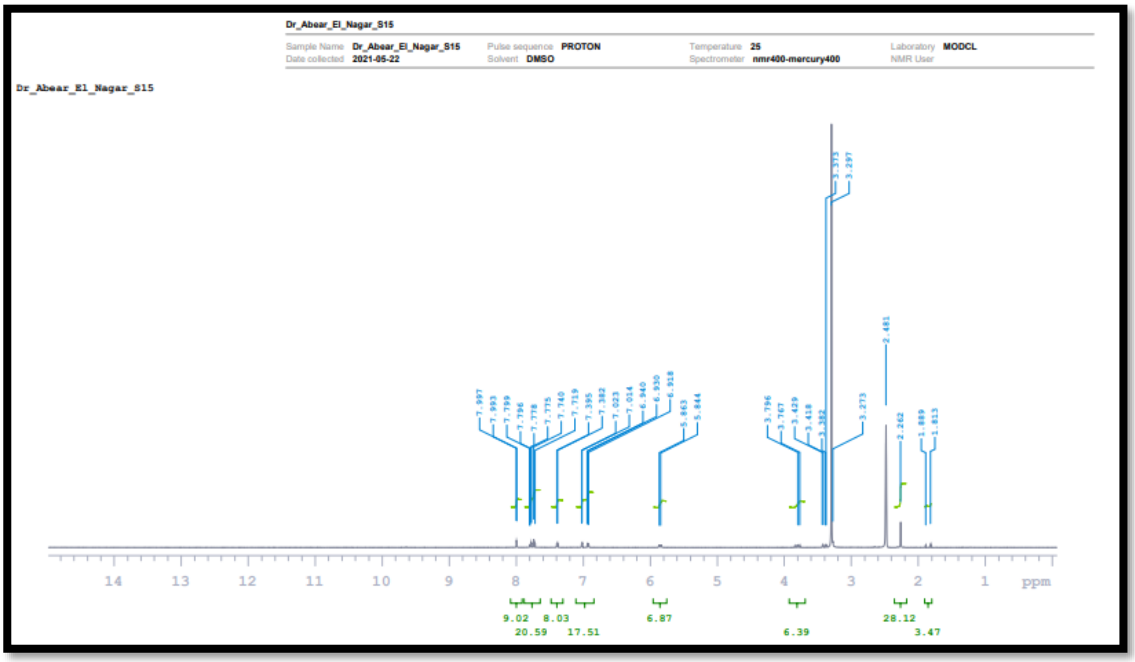

Figure S34. ^{1}H NMR (400 MHz, DMSO-d_6) spectrum of compound **8b**

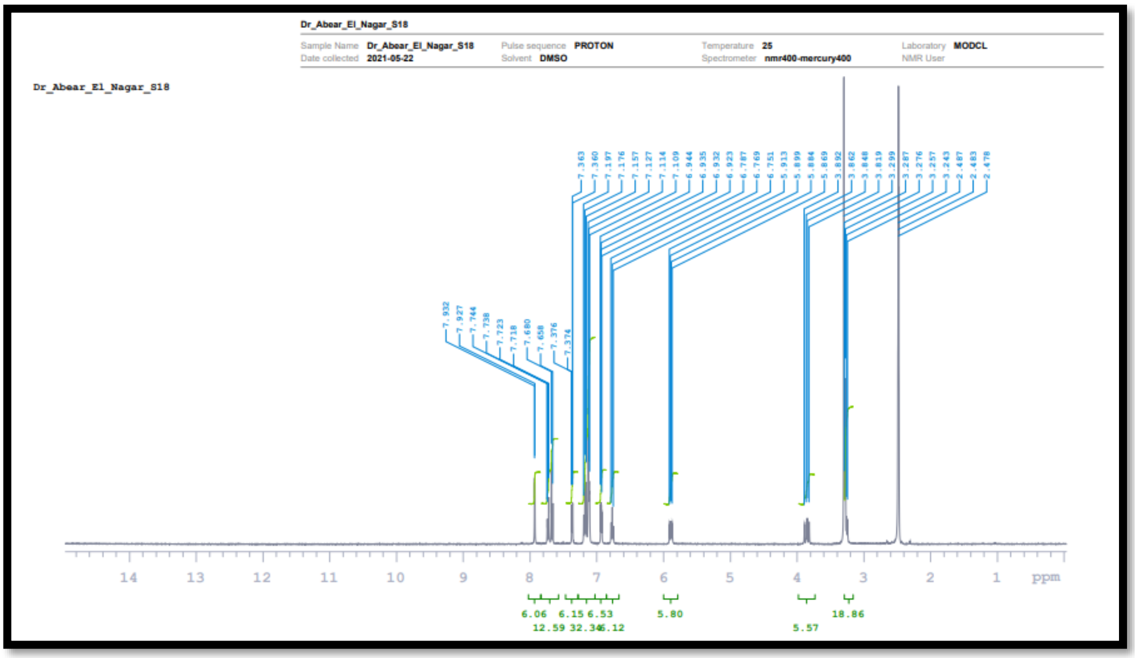

Figure S35. ^{1}H NMR (400 MHz, DMSO-d_6) spectrum of compound **9b**

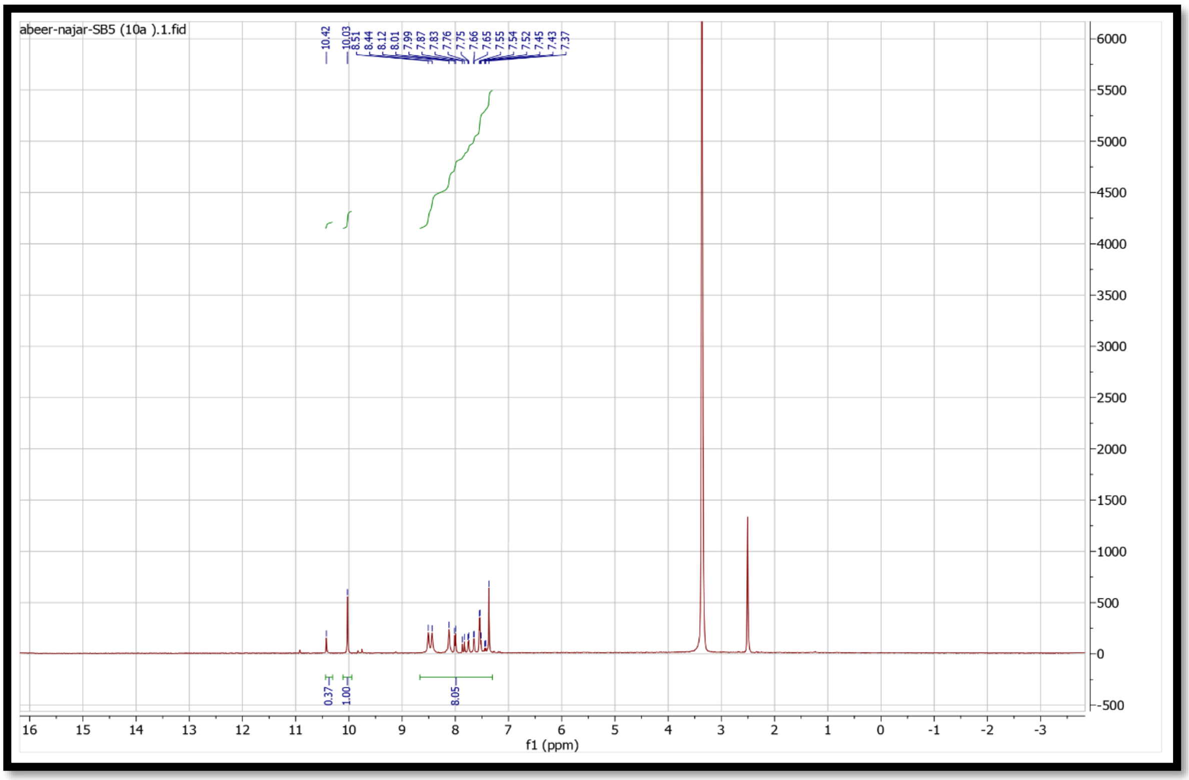

Figure S36. ^{1}H NMR (400 MHz, DMSO-d_6) spectrum of compound **10a**

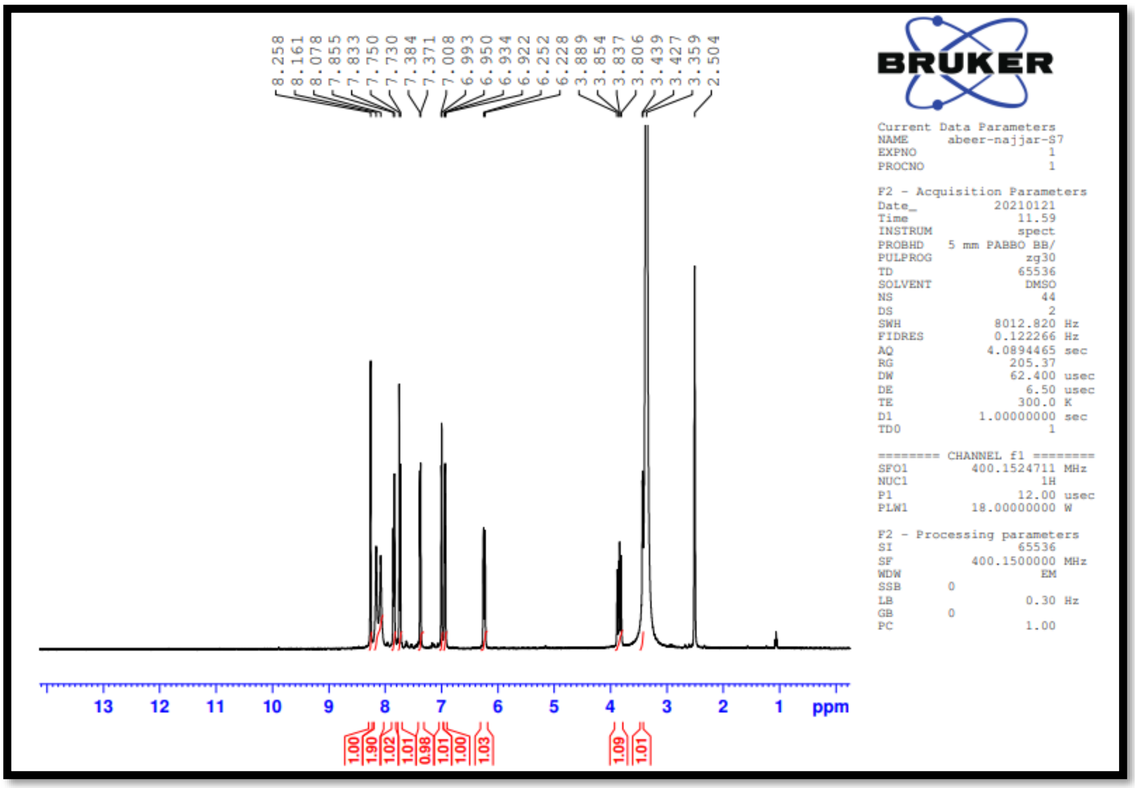

Figure S37. ^{1}H NMR (400 MHz, DMSO-d_6) spectrum of compound **10b**

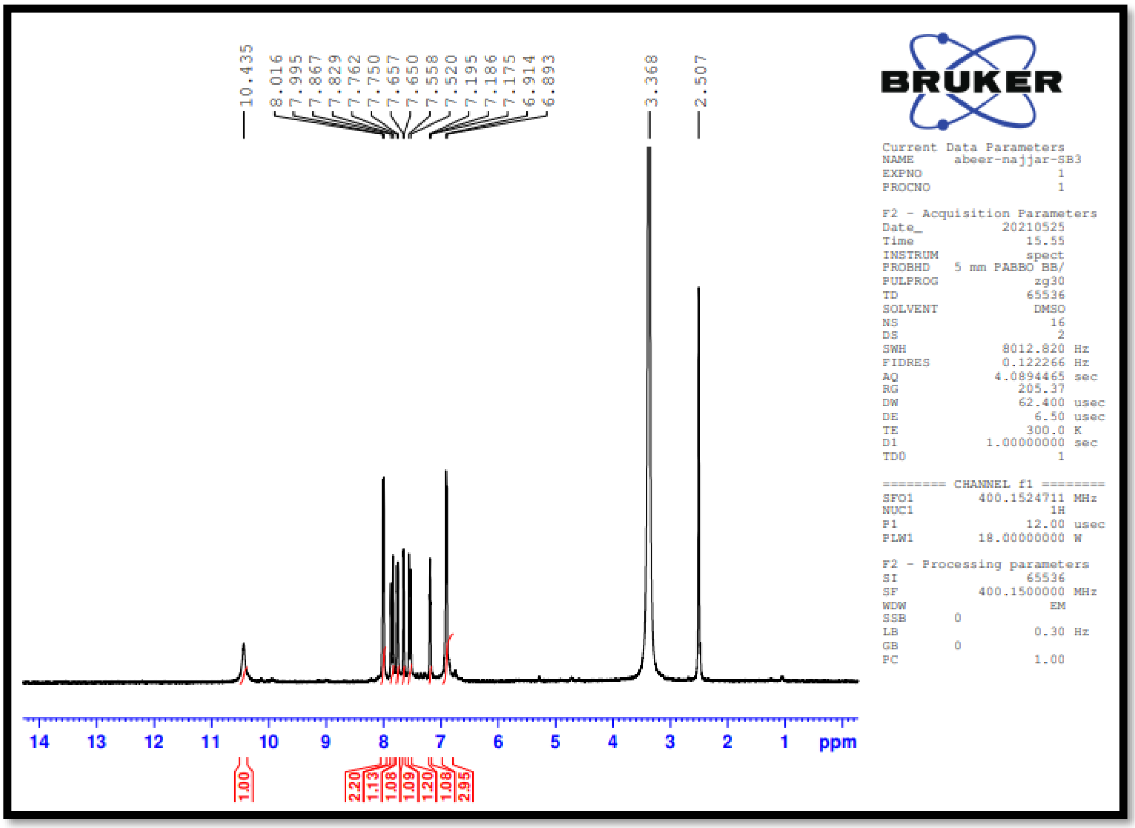

Figure S38. ^{1}H NMR (400 MHz, DMSO-d_6) spectrum of compound **11a**

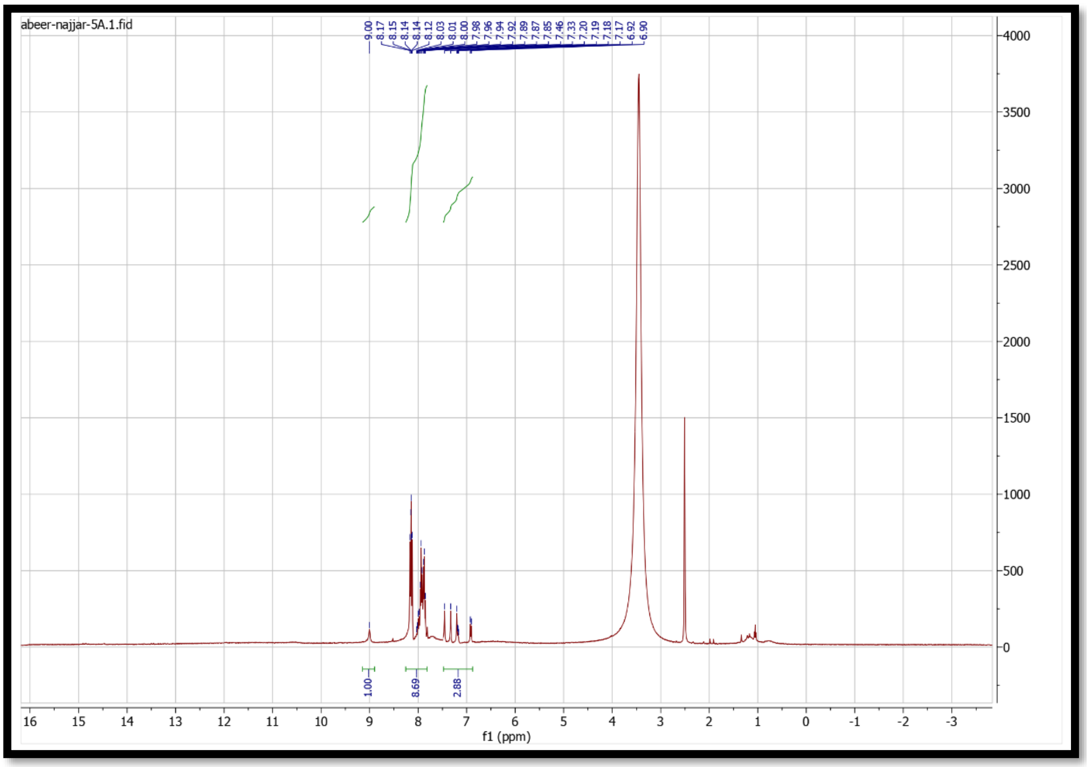

Figure S39. ^{1}H NMR (400 MHz, DMSO-d_6) spectrum of compound **12a**

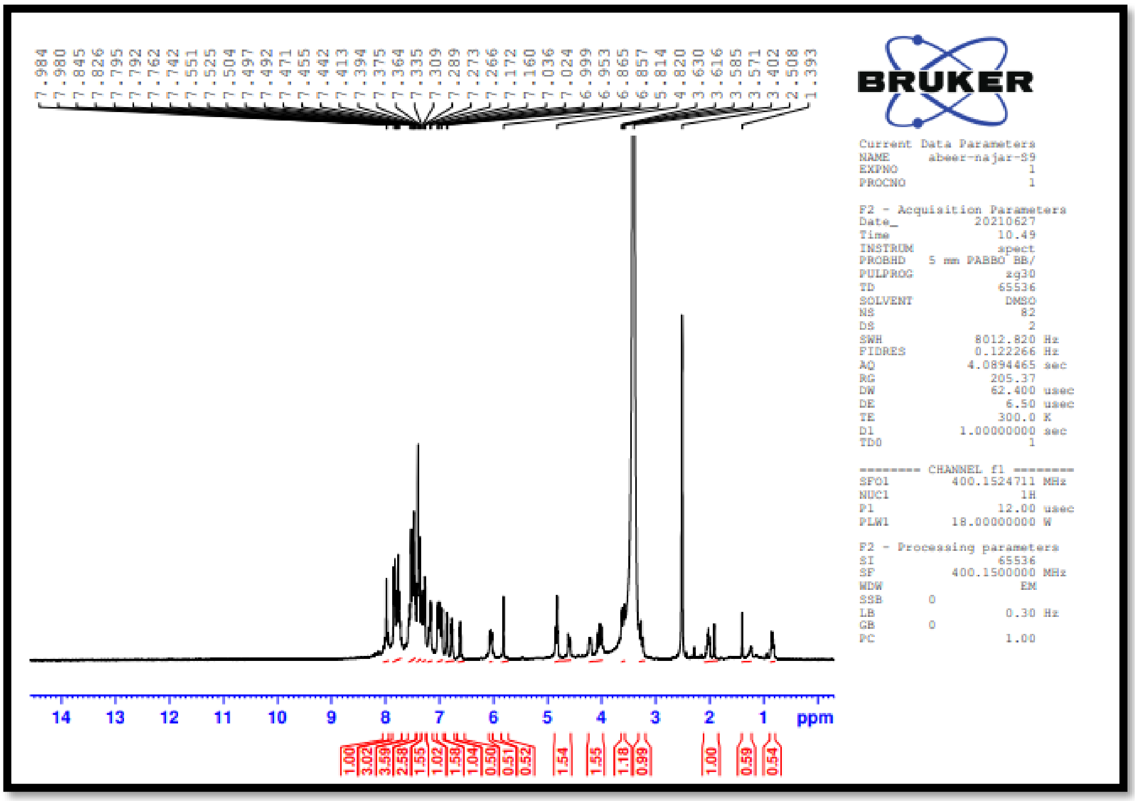

Figure S40. ^{1}H NMR (400 MHz, DMSO-d_6) spectrum of compound **12b**

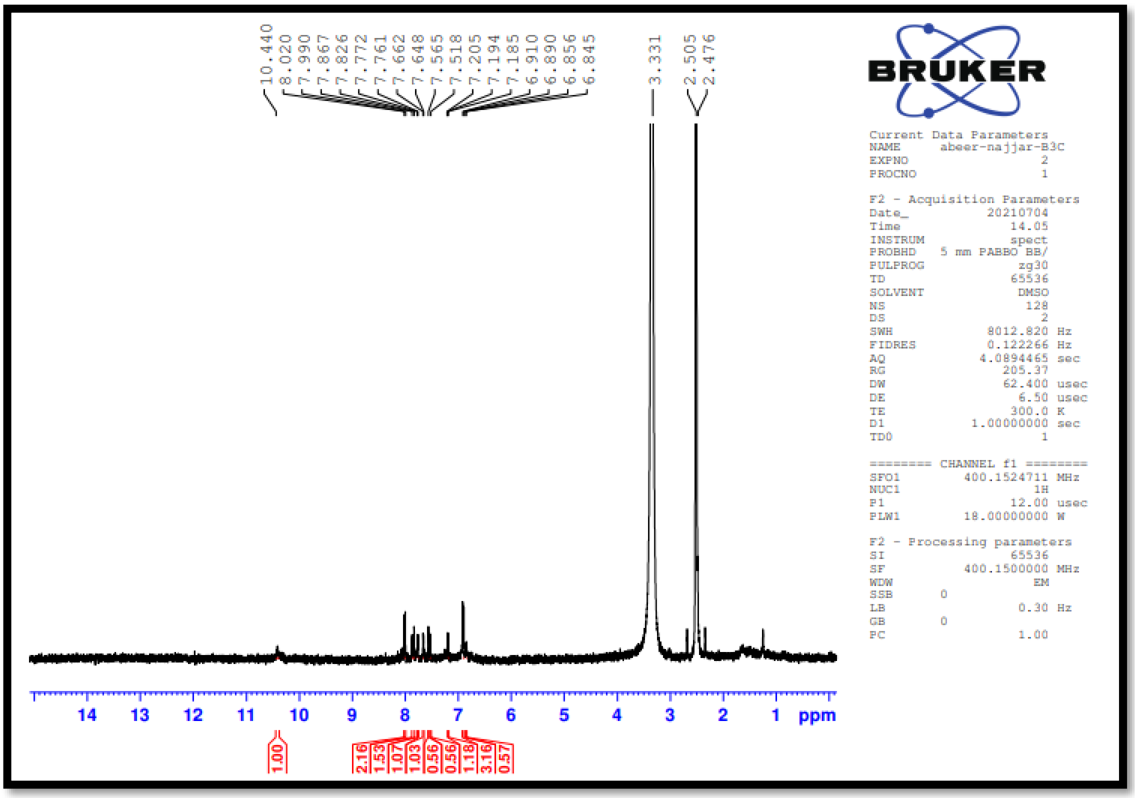

Figure S41. ^{1}H NMR (400 MHz, DMSO-d_6) spectrum of compound **13a**

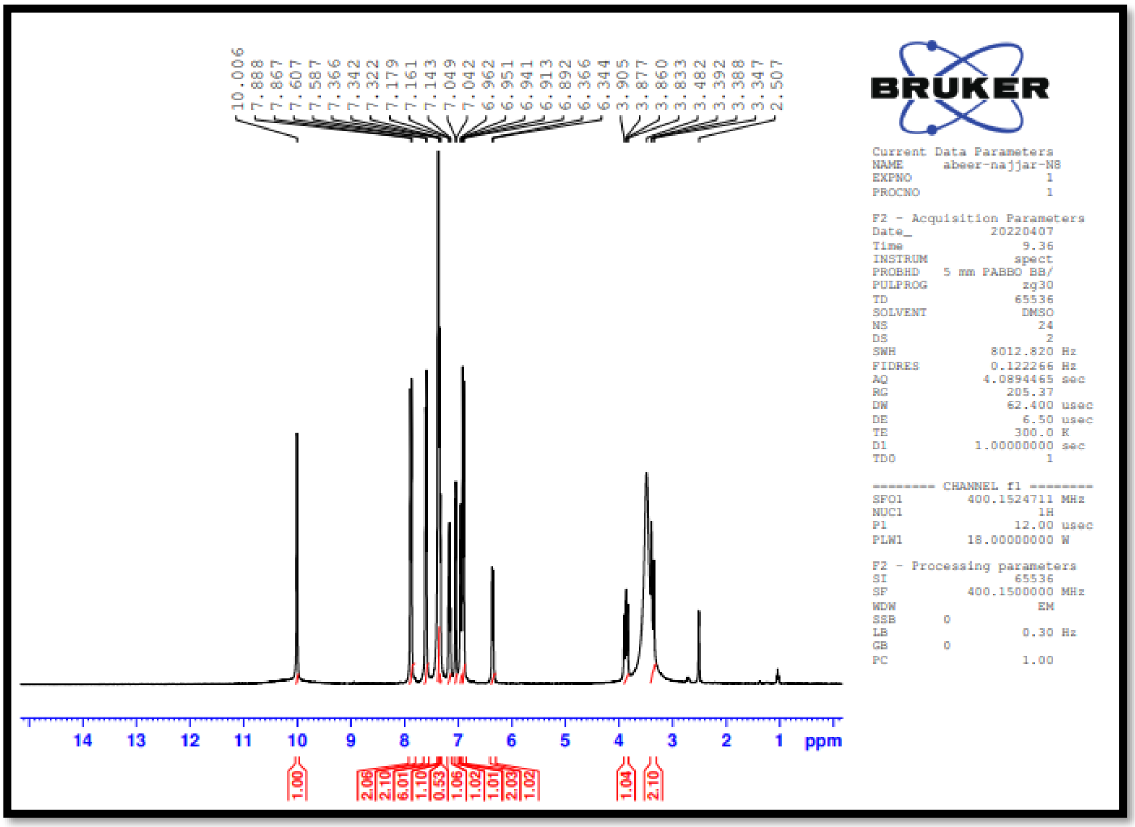

Figure S42. ^{1}H NMR (400 MHz, DMSO-d_6) spectrum of compound **14a**

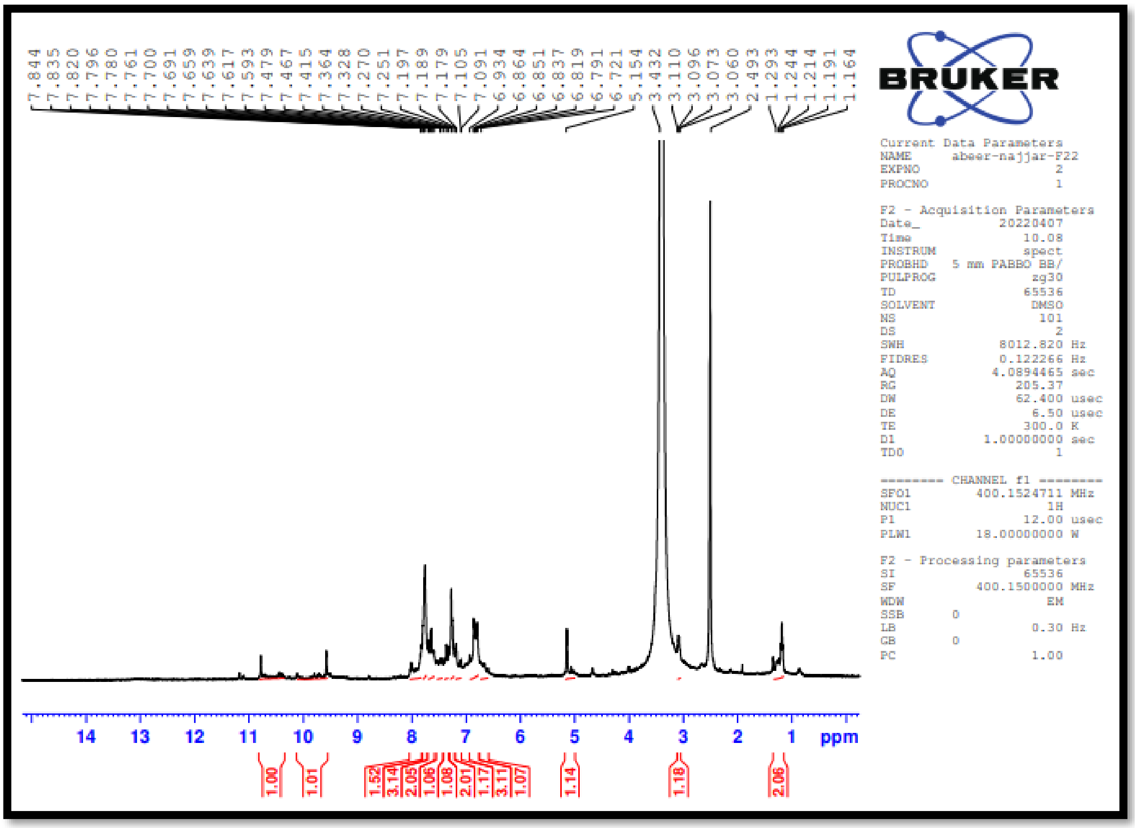

Figure S43. ^{1}H NMR (400 MHz, DMSO-d_6) spectrum of compound **16a**

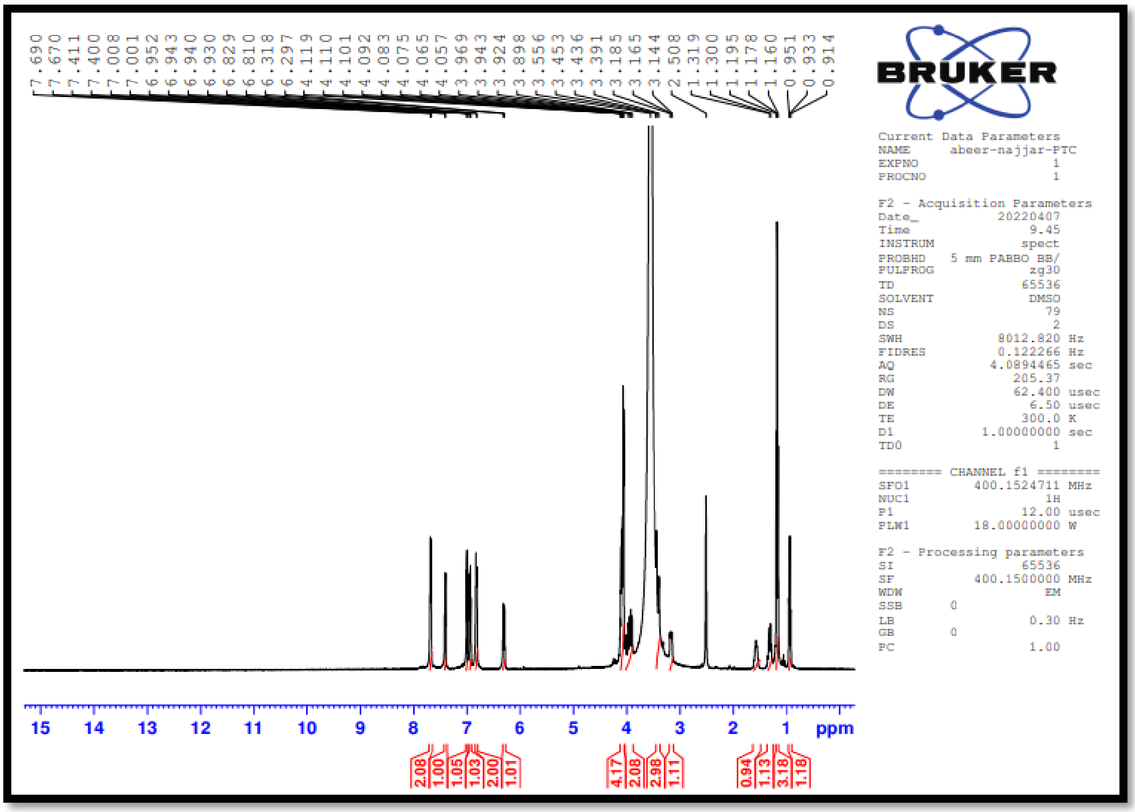

Figure S44. ^{1}H NMR (400 MHz, DMSO-d_6) spectrum of compound **17a**

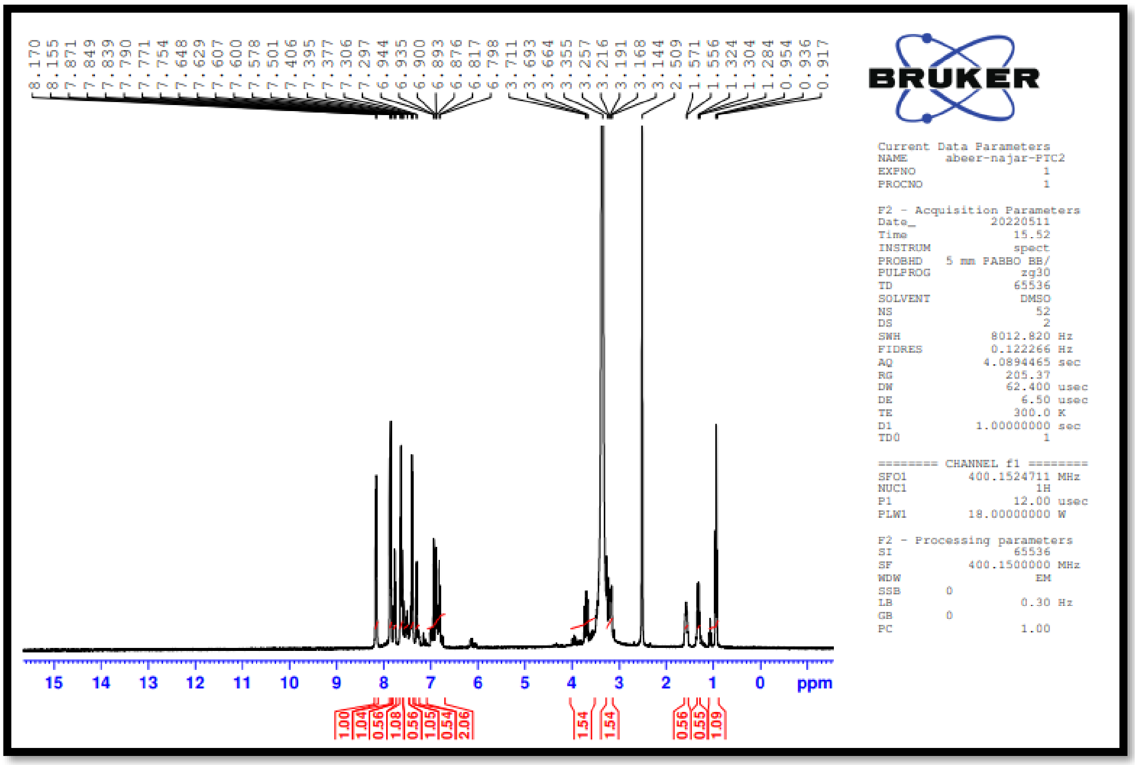

Figure S45. ^{1}H NMR (400 MHz, DMSO-d_6) spectrum of compound **18a**

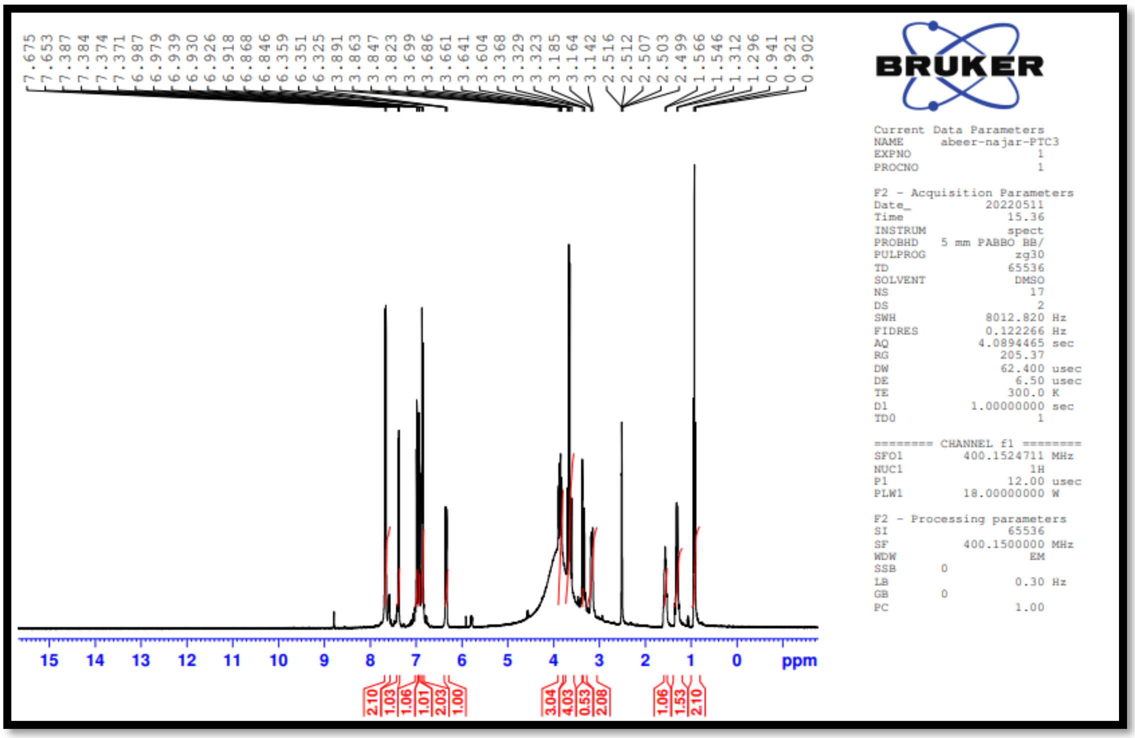

Figure S46. ^{1}H NMR (400 MHz, DMSO-d_6) spectrum of compound **19a**

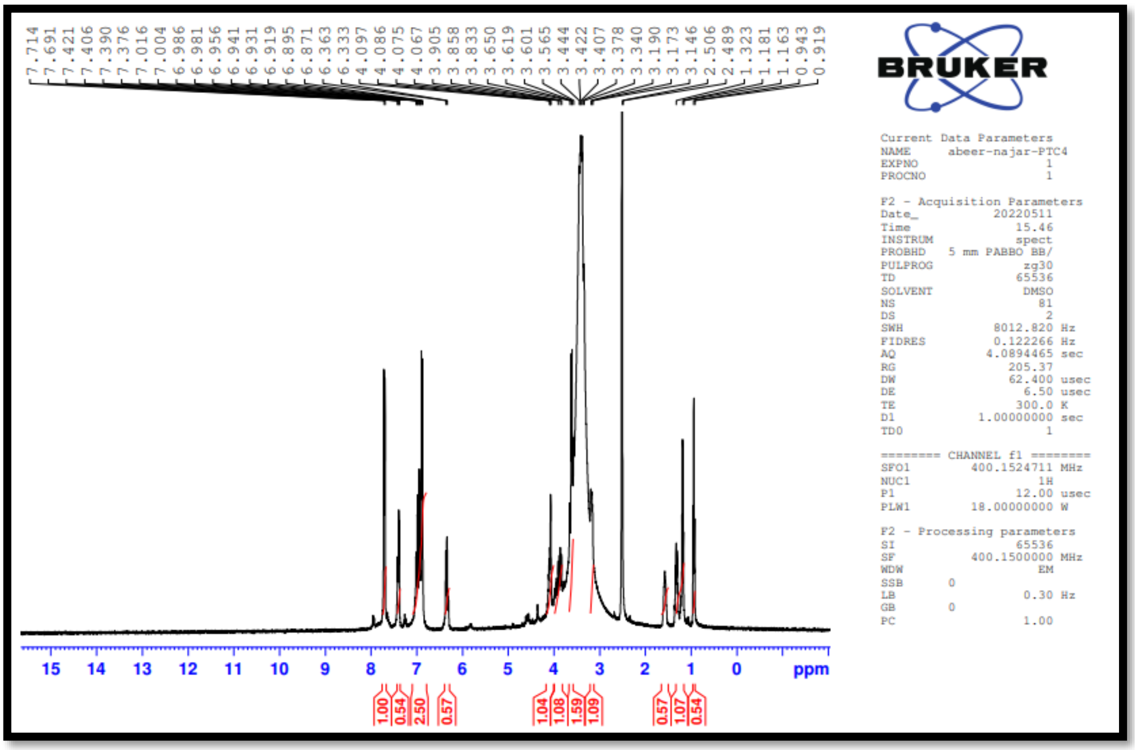

Figure S47. ^{1}H NMR (400 MHz, DMSO-d_6) spectrum of compound **20a**

Mass spectral data of the target compounds (1b,3a,10b,11a, and 18a)

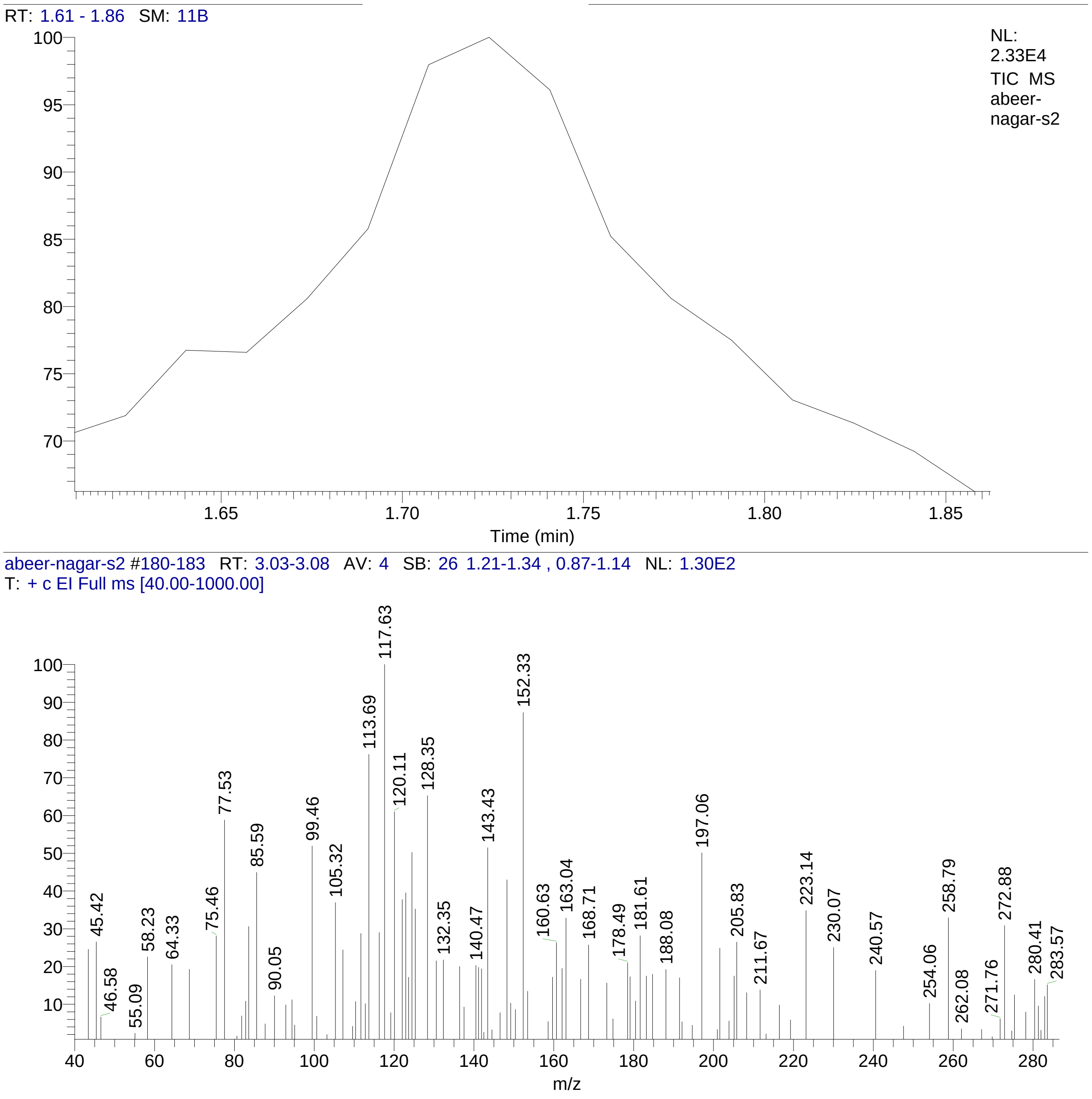

Figure S49. Mass spectrum of compound **1b**

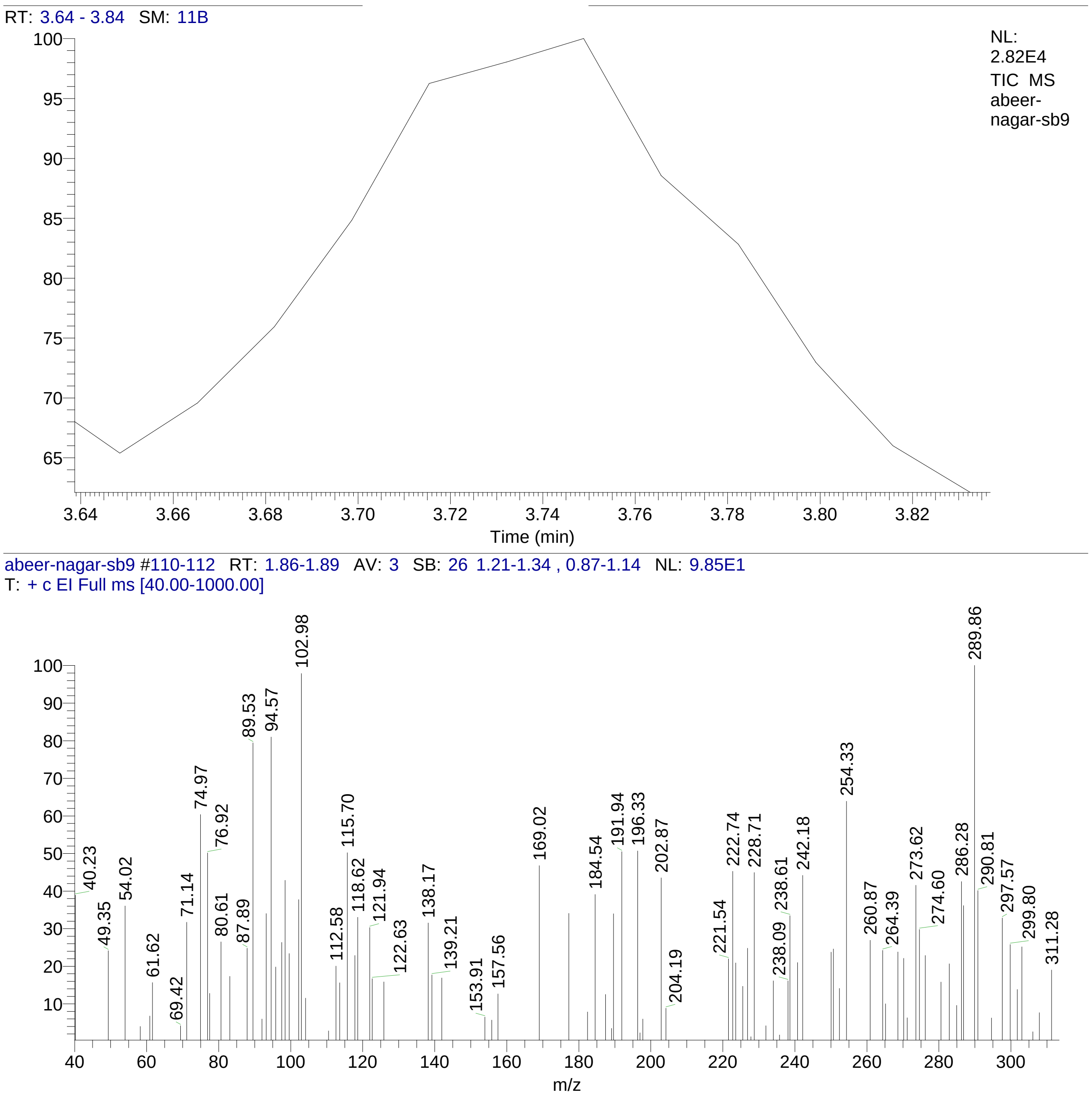

Figure S50. Mass spectrum of compound **3a**

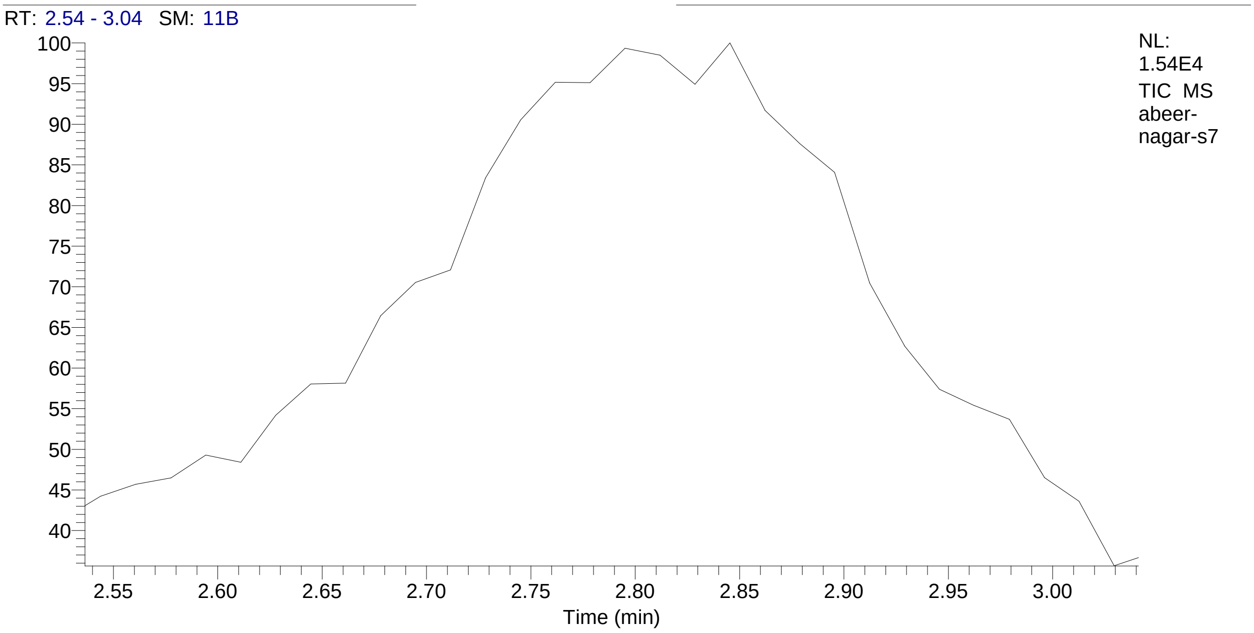

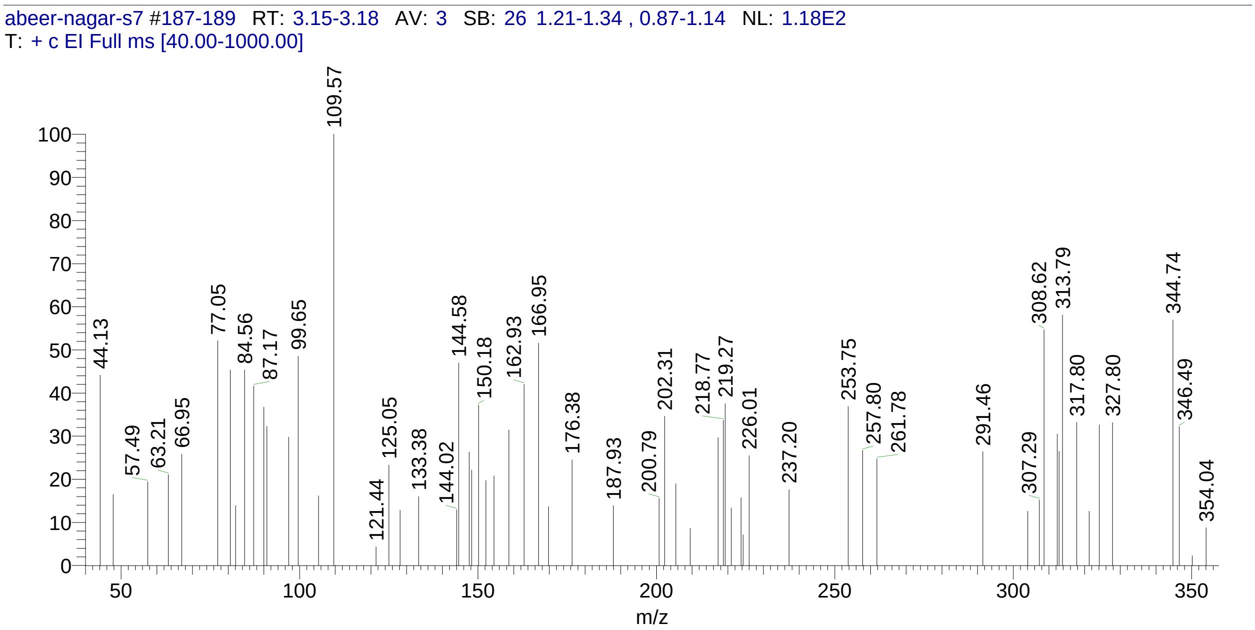

Figure S51. Mass spectrum of compound **10b**

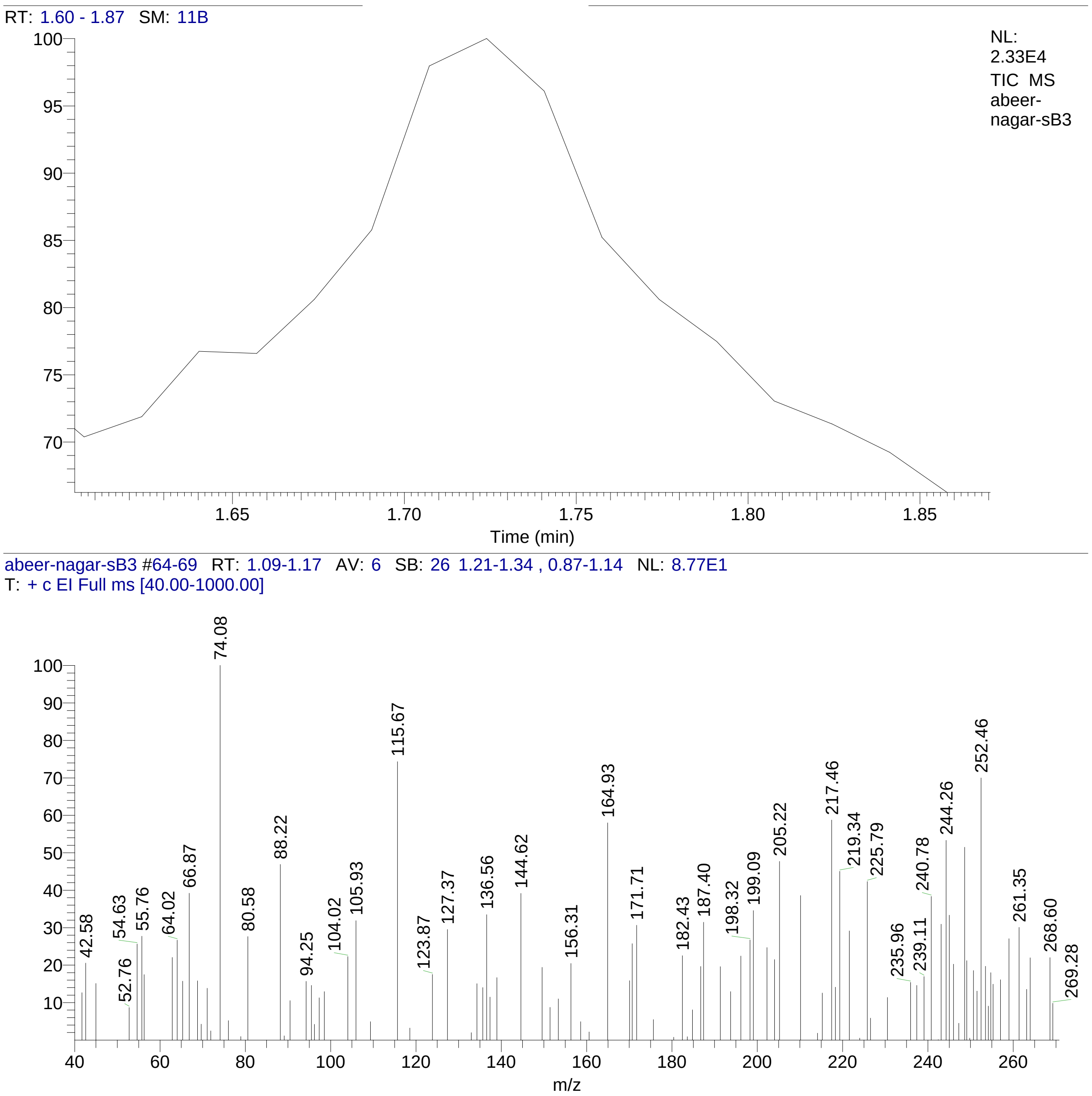

Figure S52. Mass spectrum of compound **11a**

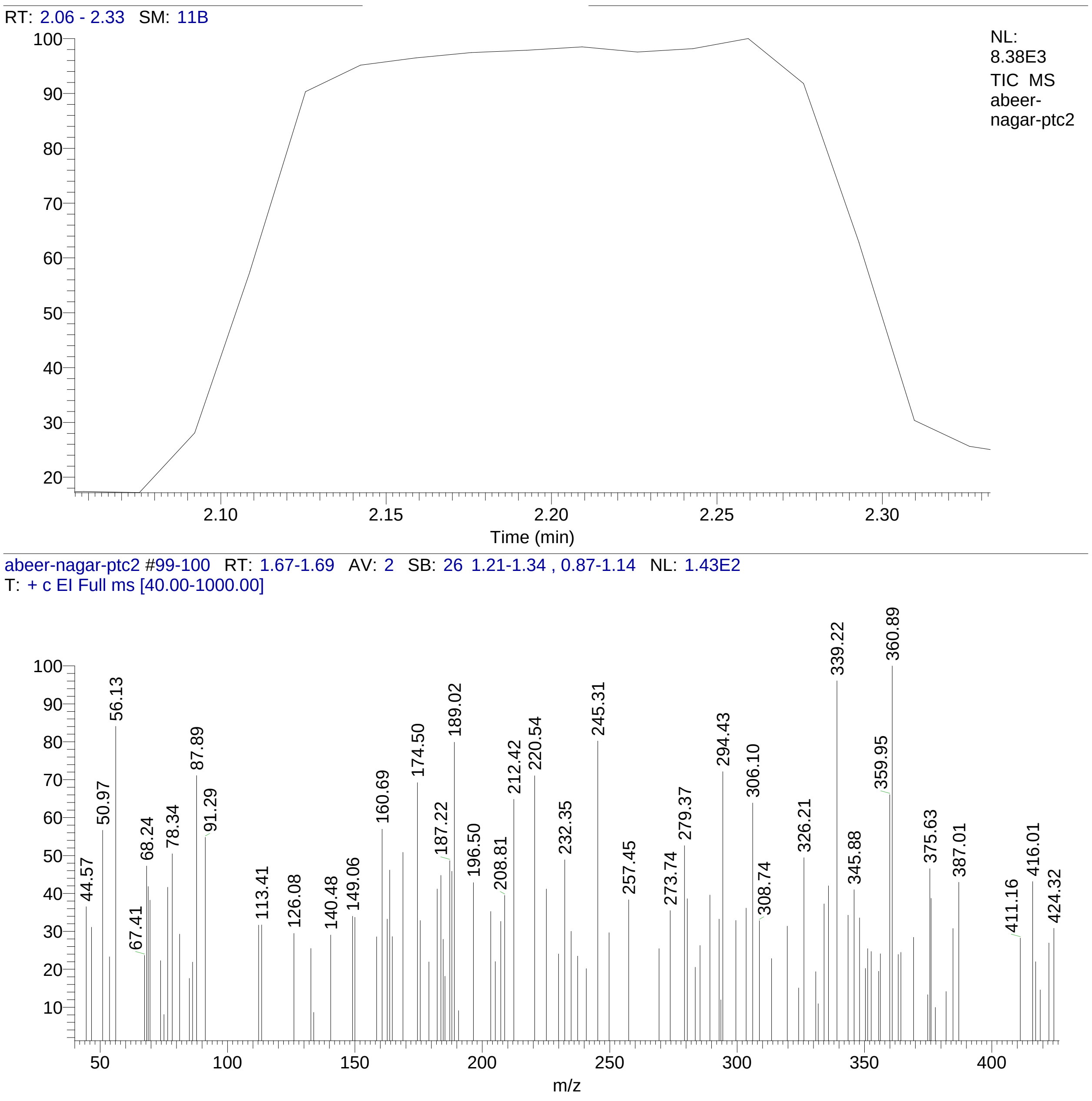

Figure S53. Mass spectrum of compound **18a**

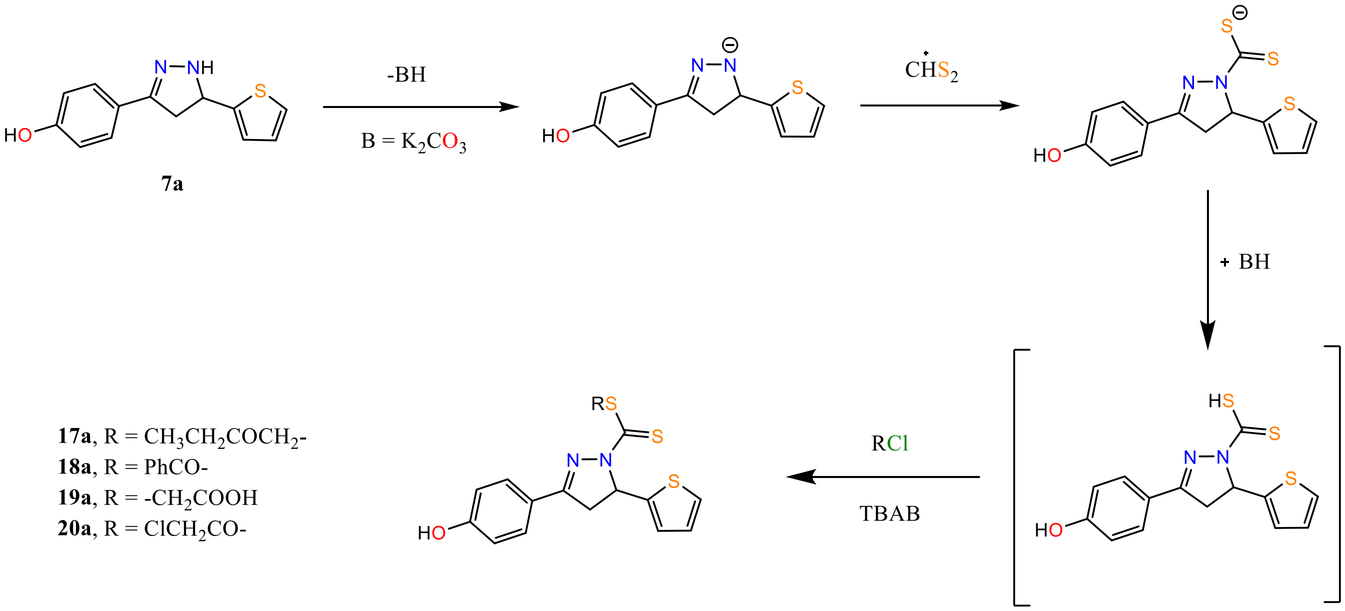

Scheme S1. Mechanism for the formation of target compounds **17a, 18a, 19a,** and **20a**.
